# Two divergent haplotypes from a highly heterozygous lychee genome suggest independent domestication events for early and late-maturing cultivars

**DOI:** 10.1038/s41588-021-00971-3

**Published:** 2022-01-03

**Authors:** Guibing Hu, Junting Feng, Xu Xiang, Jiabao Wang, Jarkko Salojärvi, Chengming Liu, Zhenxian Wu, Jisen Zhang, Xinming Liang, Zide Jiang, Wei Liu, Liangxi Ou, Jiawei Li, Guangyi Fan, Yingxiao Mai, Chengjie Chen, Xingtan Zhang, Jiakun Zheng, Yanqing Zhang, Hongxiang Peng, Lixian Yao, Ching Man Wai, Xinping Luo, Jiaxin Fu, Haibao Tang, Tianying Lan, Biao Lai, Jinhua Sun, Yongzan Wei, Huanling Li, Jiezhen Chen, Xuming Huang, Qian Yan, Xin Liu, Leah K. McHale, William Rolling, Romain Guyot, David Sankoff, Chunfang Zheng, Victor A. Albert, Ray Ming, Houbin Chen, Rui Xia, Jianguo Li

**Affiliations:** 1 State Key Laboratory for Conservation and Utilization of Subtropical Agro-Bioresources, Guangdong Laboratory for Lingnan Modern Agriculture, Key Laboratory of Biology and Germplasm Enhancement of Horticultural Crops, Ministry of Agriculture and Rural Affairs, Guangdong Litchi Engineering Research Center, College of Horticulture, South China Agricultural University, Guangzhou, China; 2 Key Laboratory of South Subtropical Fruit Biology and Genetic Resource Utilization, Institute of Fruit Tree Research, Guangdong Academy of Agricultural Sciences, Ministry of Agriculture and Rural Affairs, Guangdong Provincial Key Laboratory of Tropical and Subtropical Fruit Tree Research, Guangzhou, China; 3 Danzhou Scientific Observing and Experimental Station of Agro-Environment, Ministry of Agriculture and Rural Affairs, Environment and Plant Protection Institute, Chinese Academy of Tropical Agriculture Sciences, Haikou, China; 4 School of Biological Sciences, Nanyang Technological University, Singapore, Singapore; 5 Center for Genomics and Biotechnology, Haixia Institute of Science and Technology Fujian Agriculture and Forestry University, Fuzhou, China; 6 BGI-Shenzhen, Shenzhen, Guangdong, China; 7 Guangdong Key Laboratory of Microbial Signals and Disease Control, College of Plant Protection, South China Agricultural University, Guangzhou, China; 8 Horticultural Research Institute, Guangxi Academy of Agricultural Sciences, Nanning, China; 9 College of Natural Resources and Environment, South China Agricultural University, Guangzhou, China; 10 Department of Plant Biology, University of Illinois at Urbana-Champaign, Urbana, IL USA; 11 Institute of Tropical and Subtropical Cash Crops, Yunnan Academy of Agricultural Sciences, Baoshan, China; 12 Department of Biological Sciences, University at Buffalo, Buffalo, NY USA; 13 Key Laboratory for Tropical Fruit Biology of Ministry of Agriculture and Rural Affair, South Subtropical Crops Research Institute, Chinese Academy of Tropical Agriculture Sciences, Zhanjiang, China; 14 Department of Horticulture and Crop Sciences and Center for Applied Plant Sciences, The Ohio State University, Columbus, OH USA; 15 Center for Applied Plant Sciences, The Ohio State University, Columbus, OH USA; 16 IRD, UMR DIADE, EVODYN, Montpellier, France; 17 Department of Mathematics and Statistics, University of Ottawa, Ottawa, Ontario Canada

**Keywords:** Plant genetics, Genomics

## Abstract

Lychee is an exotic tropical fruit with a distinct flavor. The genome of cultivar ‘Feizixiao’ was assembled into 15 pseudochromosomes, totaling ~470 Mb. High heterozygosity (2.27%) resulted in two complete haplotypic assemblies. A total of 13,517 allelic genes (42.4%) were differentially expressed in diverse tissues. Analyses of 72 resequenced lychee accessions revealed two independent domestication events. The extremely early maturing cultivars preferentially aligned to one haplotype were domesticated from a wild population in Yunnan, whereas the late-maturing cultivars that mapped mostly to the second haplotype were domesticated independently from a wild population in Hainan. Early maturing cultivars were probably developed in Guangdong via hybridization between extremely early maturing cultivar and late-maturing cultivar individuals. Variable deletions of a 3.7 kb region encompassed by a pair of *CONSTANS*-like genes probably regulate fruit maturation differences among lychee cultivars. These genomic resources provide insights into the natural history of lychee domestication and will accelerate the improvement of lychee and related crops.

## Abstract (web summary)

Two divergent haplotypes from a highly heterozygous lychee genome of the cultivar ‘Feizixiao’ and resequencing of 72 lychee accessions provide insights into the genome evolution and domestication history of lychee.

## Main

**Fig. 1:**
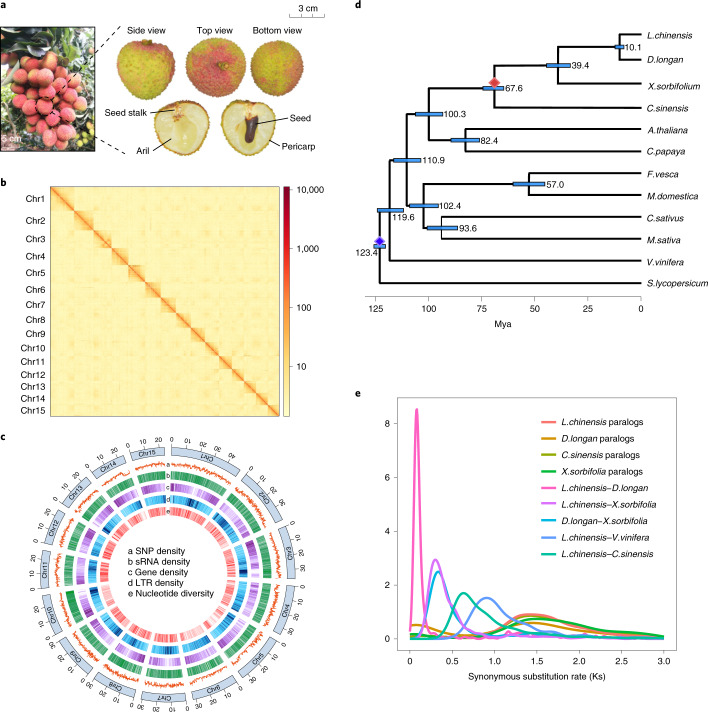
Assembly, composition and evolution of the lychee genome. **a**, Schematic presentation of lychee fruit. Scale bar, 5 cm (left), 3 cm (right). **b**, Contact map of Hi-C links among 15 pseudochromosomes. **c**, Lychee genomic features. **d**, Phylogenetic and molecular clock dating analysis of the lychee genome with 13 other species, based on single-copy orthogroup data. The red diamond indicates our fossil calibration point^26^, and the blue diamond represents *S. lycopersicum* divergence time^27^. The light blue bars at the internodes represent 95% confidence intervals for divergence times. **e**, Synonymous substitution rate (*K*_s_) density distributions of syntenic lychee paralogs and orthologs compared with other eudicot species. Source data

Lychee (*Litchi chinensis* Sonn., Sapindaceae) is an important tropical fruit tree species worldwide, and a valuable fruit for which the edible portion is an aril (Fig. 1a and Supplementary Fig. 1). Lychee is cultivated in over 20 countries, where it is an integral part of local economies. Its desirable characteristics include outstanding nutritional profile, exotic flavor and an appealing fruit color, making it one of the most attractive tropical or subtropical fruits on the international market^1^.

Lychee has been cultivated in southern China for millennia. The earliest record of lychee cultivation traces back to the second century bc. In the ancient Tang Dynasty, roughly 1,300–1,100 years ago, the Emperor set up a courier service with fast horse relays to transport fresh lychee from southern China to the imperial court because of the prodigious flavor of this spoilable fruit. Lychee trees have the longest productive lifespans among tropical and subtropical fruit trees. The oldest lychee tree, ‘Songxiang’, from Fujian, China, is over 1,250 years old, and is still fruiting today. This long cultivation history has facilitated the generation of diverse lychee germplasm. Over 400 lychee cultivars are preserved at the National Lychee Germplasm Resources in Guangzhou, China.

Lychee cultivars are classified into three groups based on the fruit maturation period: extremely early maturing cultivars (EEMC), early-to-intermediate-maturing cultivars (EMC) and late-maturing cultivars (LMC)^2,3^. EEMCs are rare and of little production value, while cultivars with better fruit quality always belong to the LMC group. About 80% of fruits are produced within a short period from early June to mid-July. The extreme perishability of lychee fruit renders it impossible to sustain a fresh, year-round supply.

Lychee originated in broad, yet isolated and distant, regions of southern China, where numerous wild lychees exist in the Hainan and Yunnan rainforests, and hilly areas of western Guangdong and eastern Guangxi. However, the exact center(s) of origin and the history of lychee domestication remains unknown. To address these questions and provide a genome-enabled breeding platform, we generated a high-quality reference genome of the highly heterozygous cultivar ‘Feizixiao’ (2*n* = 2× = 30) and resequenced 72 wild or cultivated accessions to explore the structure and evolution of the lychee genome as well as its origin and domestication history. We also investigated expression patterns of allelic genes and potential regulatory mechanisms involved in timing of flowering and fruit maturation. These results will improve our understanding of the lychee genome, accelerating genetic improvement of lychee and its relatives in Sapindaceae.

## Results

### Sequencing, assembly and annotation of the lychee genome

**Extended Data Fig. 1:**
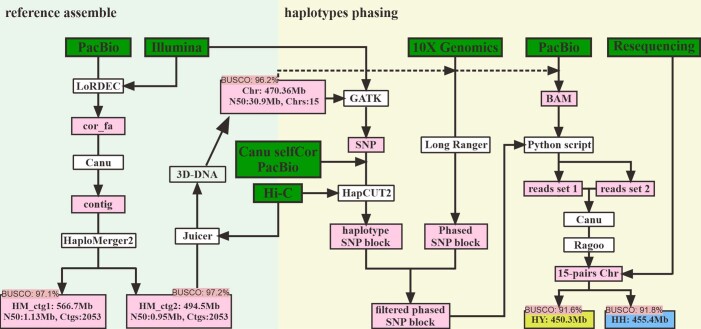
Workflow of genome assembly and haplotype phasing. PacBio subreads were corrected using Illumina PE reads with LoRDEC, followed by assembly with the Canu software. Afterward, assembled contigs were separated into two contig collections using HaploMerger2, and one of the collections (HM_ctg2) with similar genome size to our flow cytometric estimation was used for the anchoring of assembled contigs into pseudochromosomes using Juicer and 3D-DNA based on Hi-C data. Finally, a reference genome, totaling 470 M with 15 chromosomes, was obtained. **Haplotype phasing**: Illumina reads were mapped against the reference genome and the resulting alignment file was used for variant calling following the GATK4 pipeline. Subsequently, hard-filter passing and biallelic SNPs (11.2 M) were kept for haplotype phasing using HapCUT2. Meanwhile, 10X Genomics reads were used for calling phased SNP blocks. SNPs (6.4 M) combined from the above two sets of SNP blocks were then retained for PacBio reads phasing using the approach first developed for the phasing of sex chromosomes (Zhang et al., 2020). After that, phased reads were de novo assembled with Canu independently, and contigs were linked with the help of the reference genome using RaGOO software. Eventually, 15 pairs of homologous pseudo-chromosomes were obtained and divided into two haplotypes, HY and HH, according to the mapping rates of resequencing data.

We generated 58.6 Gb PacBio long reads (124× coverage) from the lychee cultivar ‘Feizixiao’ and 86.25 Gb (184× coverage) clean Illumina short reads from libraries with different insert sizes (Supplementary Table 1). Long reads corrected with shotgun reads were used for de novo assembly, resulting in a 962 Mb draft genome with a contig N50 of 752 kb (Supplementary Table 2). In contrast to genome size estimates from flow cytometry (~500 Mb)^4^ or 19-mer analysis (~460 Mb), the initial assembly was almost twice as large (Supplementary Tables 3,4). We interpreted this as the result of the high heterozygosity (from k-mers, 2.27%) of ‘Feizixiao’, such that the initial assembly contained two divergent haplotypes. We therefore separated these haplotypes using HaploMerger2 (ref. ^5^) (Supplementary Tables 5 and 6). The haplotype, similar to the flow cytometry estimate, was anchored into pseudochromosomes using the physical map generated with high-throughput chromatin conformation capture (Hi-C) technology (144× coverage). Eventually, a reference genome of 15 pseudochromosomes (470 Mb; Fig. 1b, Extended Data Fig. 1 and Supplementary Table 7) was obtained with 96.2% completeness in conserved single-copy protein-coding sequences (BUSCO v.3 and the eudicotyledons_odb10 database). For annotation, mRNA sequencing (RNA-seq) data were aligned to the reference genome, and 31,896 putative protein-coding gene models were predicted with estimated completeness of 94.8% (also using BUSCO; Fig. 1c and Supplementary Tables 8–12). These results attest to the high accuracy and completeness of our lychee genome assembly.

**Extended Data Fig. 2:**
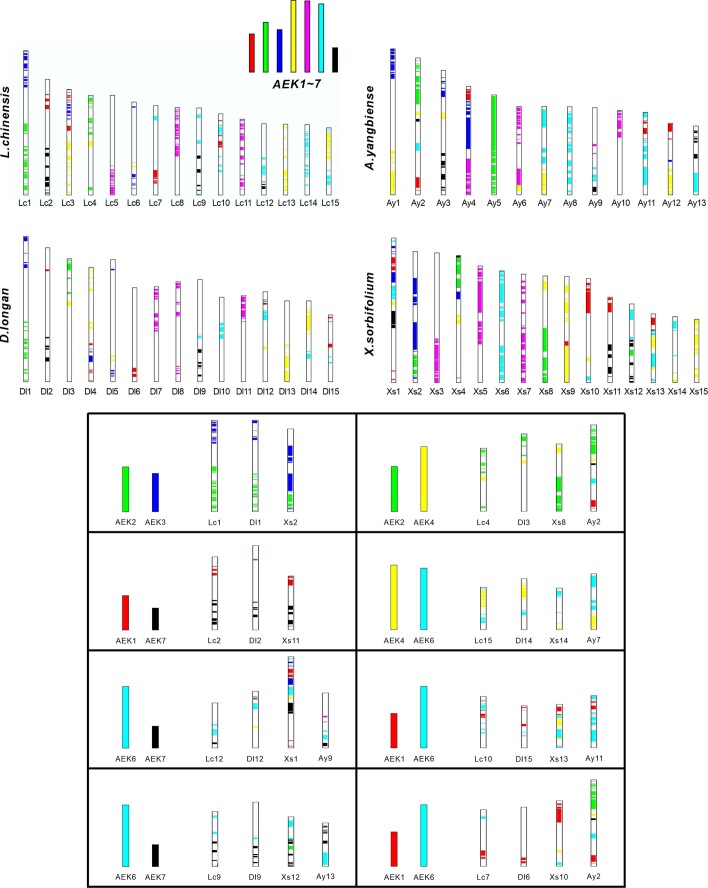
Multiple chromosomal fusions were detected among genomes of Sapindaceae species. Eight chromosomal fusions were detected across four Sapindaceae species. Each species has 15 chromosomes except *A. yangbiense*, which has 13 chromosomes. All these chromosomes descended from an ancestral core eudicot that had 21 chromosomes after the γ triplication event.

Lychee was estimated to have diverged from yellowhorn (*Xanthoceras sorbifolium*) and citrus (*Citrus sinensis*) around 39.4 (34.3–44.7) and 67.6 (64.5–72.2) million years ago (Mya), respectively (Fig. 1d). Peak *K*_s_ values of paralogous gene pairs from citrus, yellowhorn and longan (*Dimocarpus longan*) were similar to that of lychee (*K*_s_ = 1.43), indicating absence of further whole-genome duplications (WGDs) in Sapindaceae since the ancient gamma triplication event^6^ (Fig. 1e and Supplementary Fig. 2). A few fusion events and interchromosomal translocations occurred in Sapindaceae genomes compared with the ancestral eudicot karyotype (Extended Data Fig. 2, Supplementary Fig. 3 and Supplementary Note I).

### Origin and domestication of lychee

To explore lychee genetic variation and elucidate its evolutionary and domestication history, 72 representative accessions, including 38 wild individuals and 34 cultivars, were selected for whole-genome resequencing (Supplementary Table 13). A total of 80,235,643 variants were identified across accessions, among them 42,339,290 high-quality single nucleotide polymorphisms (SNPs) that were further analyzed. Lower genetic diversity, was identified in lychee compared with soybean^7^ and peach^8^. Surprisingly, since we had expected a domestication bottleneck, cultivated lychee showed greater diversity than wild populations (wild: π = 0.0083, Tajima’s *D* = 0.58; cultivated: π = 0.0107, Tajima’s *D* = 0.20) (Supplementary Table 14).

**Fig. 2:**
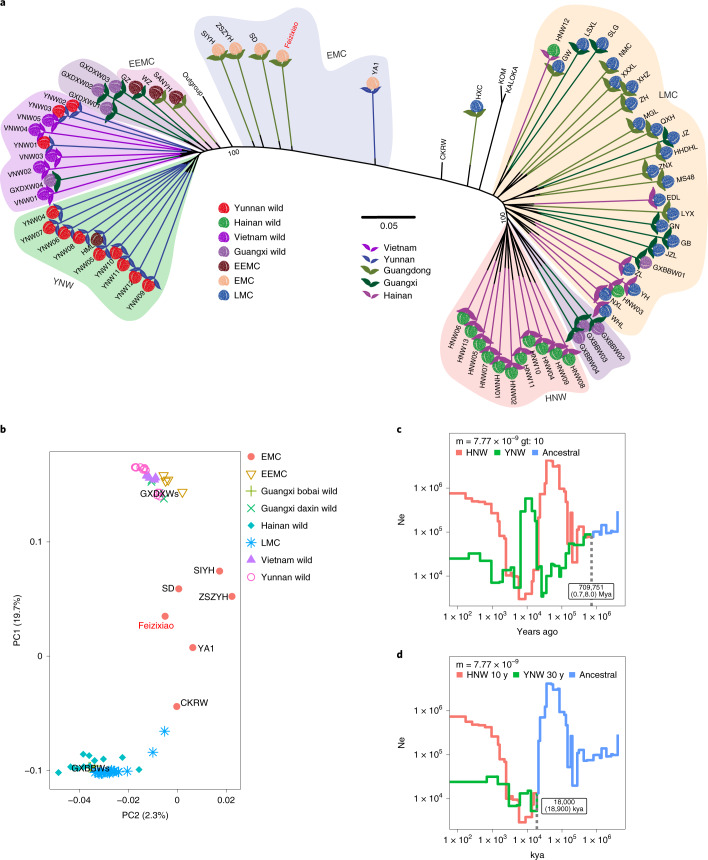
Population analyses of resequenced lychee varieties. **a**, A tree of all lychee accessions estimated based on high-quality SNPs. The black portion of each branch, representing the actual genetic distance, is extended (with colored lines) to better show the tree structure. **b**, PCA using all identified SNPs as markers closely recapitulates the tree in **a** by identifying two main clusters along PC1 (GXBBWs and LMC, versus GXDXWs) with intermediate cultivars arrayed in between (clustering with ‘Feizixiao’). **c**, The joint population history (blue line) of Yunnan wild (YNW, green line) and Hainan wild populations (HNW, red line) and their split time (dashed line) estimated using SMC++^28^. Different mutation rate-generation time combinations were used to obtain the ranges of possible split times. **d**, After correcting for inbreeding, the joint population history of Yunnan and Hainan wild populations show divergence at ~18 thousand years ago (kya) (Supplementary Note II).

Both a SNP phylogeny (Fig. 2a) and principal component (PC) analysis (PCA) (Fig. 2b) revealed that wild lychees form two distinct groups consistent with their geographic origins, with wild Yunnan accessions (YNWs) grouping with wild Vietnam accessions (VNWs), and those from Hainan (HNWs) forming another group (Fig. 2a). Guangxi wild accessions (GXW) were divided: four from Daxin County (GDXWs) clustered with YNWs and four from Bobai County (GXBBWs) grouped with HNWs (Fst; Supplementary Fig. 4 and Supplementary Table 15).

**Extended Data Fig. 3:**
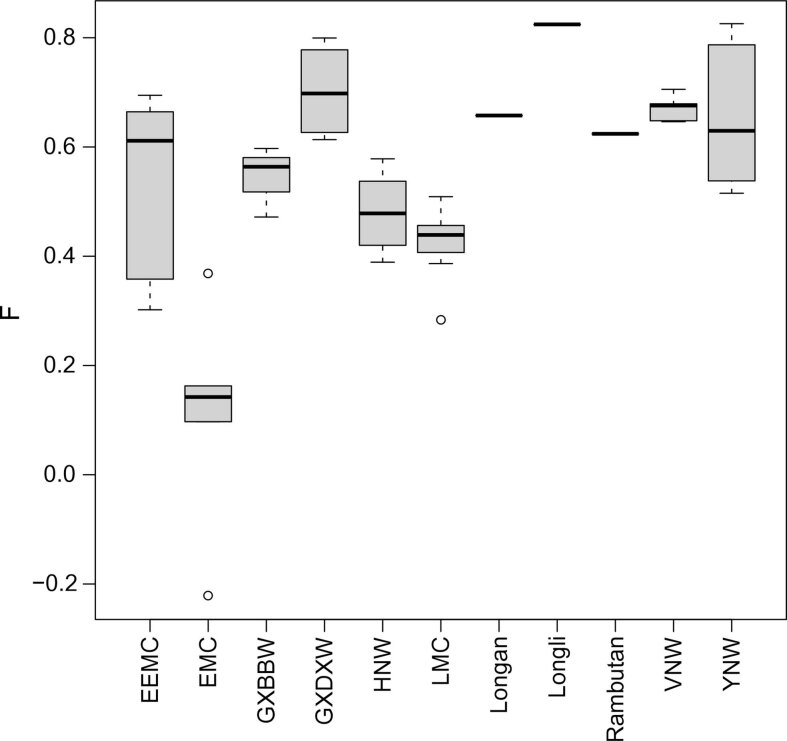
Inbreeding coefficients for different lychee subgroups. Box plots show the inbreeding coefficients from subgroups with accession numbers (left to right) are n = 4, 6, 4, 4, 13, 25, 1, 1, 1, 5 and 12, total 73 lychee accessions and 3 outgroups, respectively. Bold black line shows the median, box edges represent the 25th and 75th percentiles, whiskers represent the maximum and minimum data points within 1.5× interquartile range outside box edges, empty circles represent the outlier data.

A detailed analysis of relatedness among individuals revealed close affinities among Yunnan, Daxin and Vietnam wild populations, with several individuals showing homozygous allelic patterns corresponding to first- to third-degree relatives. In fact, two GDXWs were closely related to three YNWs and all VNWs (Supplementary Fig. 5 and Supplementary Table 16). HNWs were considerably less related, although seven individuals still showed some extent of interrelatedness. Relatedness patterns were also visible through inbreeding coefficients, with high values for Yunnan, Vietnam and Daxin populations, and lower values for the Hainan population (Extended Data Fig. 3). Similarly, linkage disequilibrium (LD) showed half of maximum *r*^2^ at about 4.6 kb for HNW, approximately 215 kb for cultivated individuals and around 91 kb for YNW, respectively (Supplementary Fig. 6). For a natural population, the LD window for YNW is relatively large and, together with low nucleotide diversity, low *F*_ST_ to Vietnam and Daxin, positive Tajima’s *D* values and high inbreeding coefficient, suggests a strong bottleneck in these populations. However, similar effects may result from greater degree of selfing in YNW, resulting in decreased effective population size (Ne).

To identify these potential bottlenecks, we modeled lychee population history employing an unfolded site frequency spectrum estimated using longli, longan and rambutan variation as ancestral states. All models displayed artefacts from extensive inbreeding in YNW, which we compensated for by adjusting generation time (Supplementary Note II). After adjustment, both YNW and HNW demonstrated Ne declines towards modern times, with current Ne estimates of ~761,000 for HNW and ~25,000 for YNW. This difference was also reflected in nucleotide diversity (π) estimates (π = 0.0069 for HNW and π = 0.0042 for YNW). Furthermore, we estimated the HNW–YNW population split time to be approximately 18,000 years ago, subject to variability due to uncertain mutation rates and generation times (Fig. 2d).

**Extended Data Fig. 4:**
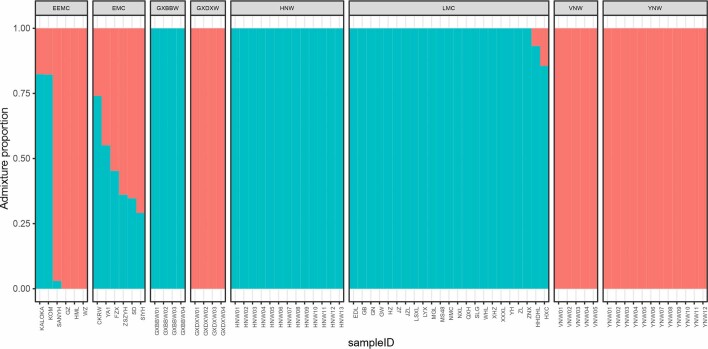
Admixture clustering shows that lychee accessions descend from two different (K = 2) ancestries. The YNW, VNW and GXDXW belong to an ancestry class (red), while the HNW and GXBBW belong to another ancestry category (blue). EEMC and LMC are grouped into the red and blue ancestries, respectively, with EMC in an intermediate state.

**Extended Data Fig. 5:**
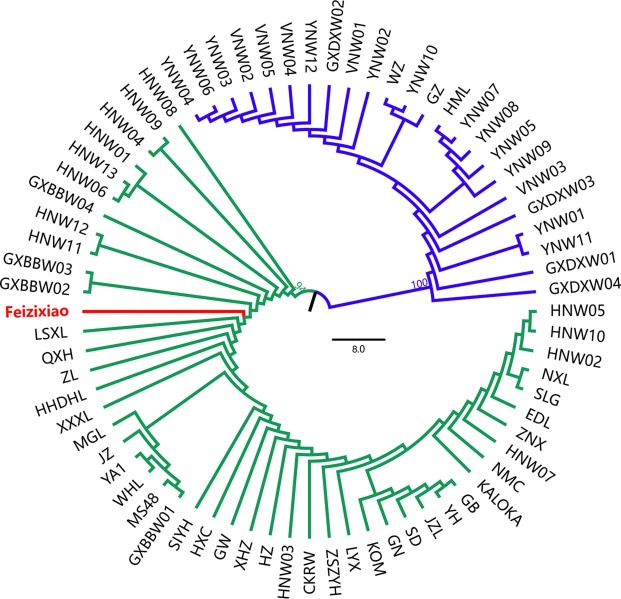
A phylogenetic tree describing genetic relationships of chloroplast genome sequences shows two distinct groups of lychee accessions. A ML tree was constructed with IQtree used auto-best model (K3Pu + F + I). Accessions from the HNW/LMC group are indicated in green, and those from the YNW/EEMC group are in blue. ‘Feizixiao’ is highlighted in red.

For cultivated lychees, in the PCA plot, EEMC cultivars clustered with YNW and LMC cultivars with HNW. EMC cultivars, including ‘Feizixiao’, were distributed intermediately, indicating admixed genetic backgrounds (Fig. 2a,b); this was confirmed by ADMIXTURE^9^ analyses (Extended Data Fig. 4) and the formal F3 admixture test^10^. The best ADMIXTURE solution identified two populations (*K* = 2), showing a clear division between EEMC/YNW and LMC/HNW populations, with EMC cultivars admixed. Hybridity was also suggested by chloroplast genome phylogenetic relationships, wherein ‘Feizixiao’ grouped with a LMC/HNW clade and other EMC accessions (Extended Data Fig. 5), implying that its maternal haplotype originated from HNW.

**Extended Data Fig. 6:**
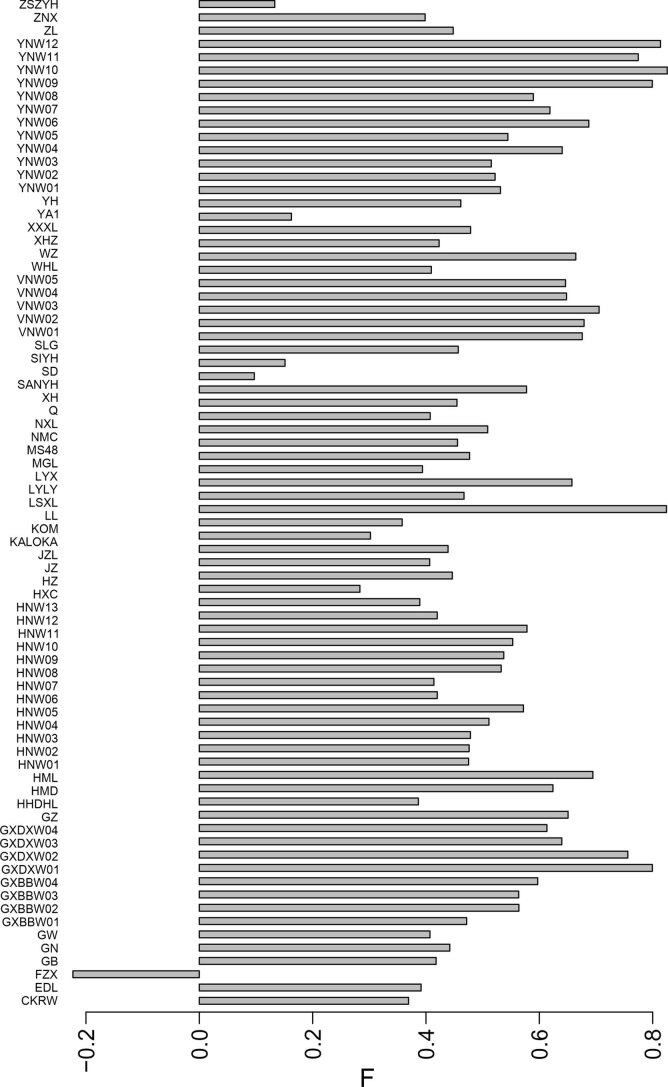
Individual inbreeding coefficients suggest that FZX may be a very recent hybrid. Compared to cultivars, the wild accessions such as YNW, VNW, GXBBW possess higher inbreeding coefficients. And the recent hybrid EMCs (ZSZYH, YA1, SD) have very low inbreeding coefficients with FZX being even negative.

Taken together, these results strongly suggest that EEMC and LMC cultivars originate from independent domestication events, from YNW and HNW, respectively. The most recent cultivar population, EMC, is probably derived from human-based hybridization between the EEMC/YNW and LMC/HNW groups, with ‘Feizixiao’ being extremely recent, as illustrated by negative inbreeding coefficient (Extended Data Fig. 6) and high heterozygosity.

### Cultivation history of lychee

**Fig. 3:**
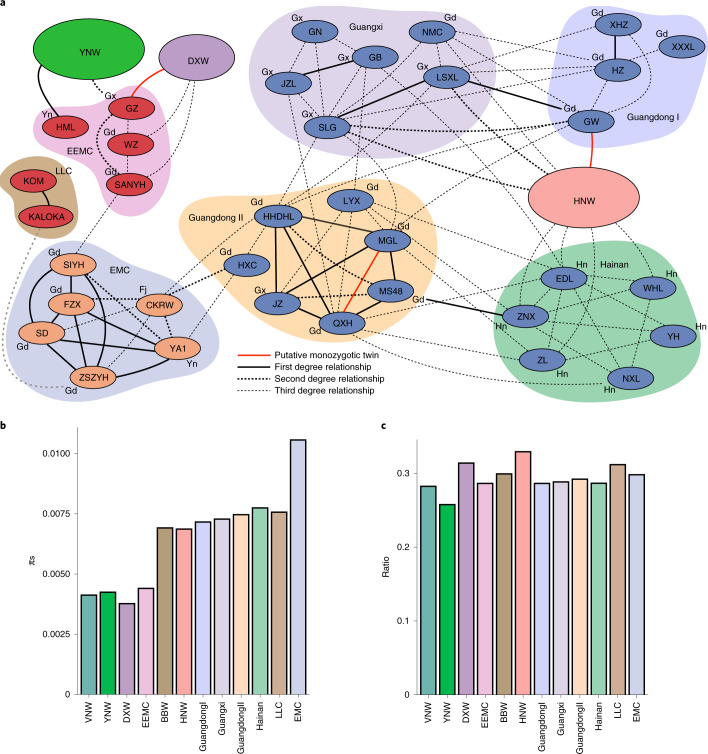
Cultivation history of lychee cultivars. **a**, Kinship relationships estimated for the different lychee cultivars and wild populations. The plot links each individual to its closest relative in terms of kinship coefficient, with different line formats illustrating the level of relationship (first, second or third degree; here individuals with highest positive score are shown linked). Estimates of the relationship level were obtained using KING software^29^ and reflect the level of shared heterozygous or homozygous haplotype blocks. DXW, Daxin wild; for the cultivars see Supplementary Table 16. The colors of the cultivar nodes show the fruit maturation type. Blue, LMC; dark red, EEMC; light brown, EMC. Geographic origins indicated next to cultivar nodes: Gd, Guangdong; Gx, Guangxi; Yn, Yunnan; Hn, Hainan. The colors encompassing cultivar nodes link different cultivation stages. **b**, The π_s_ values quantified from intergenic positions are shown for each of the populations under study, grouped according to cultivation stages shown in **a**. The differences from Guangdong II onwards are statistically significant (Supplementary Table 17). **c**, The ratios of nonsynonymous π_n_ versus π_s_ calculated for the different populations illustrate that, compared with HNW, the cultivars have less high impact mutations per neutral SNP, reflecting the breeding bottleneck. Together with the results shown in **c**, this suggests that cultivated populations may have resulted from breeding with genetically diverse populations wherein high impact mutations had been purged, possibly through inbreeding.

The long generation time and predominantly vegetative propagation of lychee cultivars has permitted the development of a cultivation history model. Interrelationship order analysis identified sample GW among LMCs, with a monozygous twin relationship with Hainan wild sample HNW13, while LSXL and SLG were second-degree related (Fig. 3a). Conservative masking of SNPs may have resulted in overestimation of shared haplotypic block lengths; hence, we examined identity-by-descent analysis, which showed relatedness of GW and HNW13 to reflect Z1-type shared heterozygous blocks, suggesting full siblings. Altogether, cultivars closest to wild populations appear to have been cultivated originally in Guangdong (‘Guangdong I’) and Guangxi (‘Guangxi’), respectively. After initial domestication, breeding materials were shared between the two locations, as evidenced by relationships between HHDHL, MGL and SLG, while eventually Guangdong became the breeding center, leading to the strongly related JZ, QXH, LYX and MS48 cultivars (‘Guangdong II’). This line was then used to establish cultivation in Hainan (‘Hainan’). Finally, two related cultivars, HHDHL and HXC, show admixture with YNW (Extended Data Fig. 4), suggesting that new breeding material was introduced at this point, possibly contributing to breeding of EMC cultivars, as shown by the second-degree relationship between HXC and CKRW.

On the other hand, EEMC cultivars GZ and HML showed first-degree relationships with YNW (Fig. 3a), suggesting they originate from a distinct breeding event. Among EMCs, ‘Feizixiao’ (FZX) probably shares the same event and subsequent breeding with all other EMC, since all were first-degree related (Fig. 3a). For possible parents, SIYH showed a third-degree relationship with SANYH (EEMC), whereas CKRW was second-degree related to HXC—an LMC. Besides CKRW, all such cultivars originate from Guangdong, suggesting this to be the initial breeding site. Therefore, EMC cultivars (for example, FZX) probably stem from hybridization between EEMC and LMC groups.

To study possible selection for hybrid vigor, we categorized the cultivars according to their relatedness and origins to compare neutral (π_s_) and high impact (π_n_) nucleotide diversities among them (Fig. 3b,c). Overall, cultivar diversities were higher than in wild populations (Fig. 3b), suggesting breeding between divergent parental populations. The diversities increase toward more recent cultivar lines, with highest levels in Guangdong II and Hainan cultivars (Fig. 3b). One possible source of this greater diversity is unsampled, divergent ancestors that were extirpated during modern agricultural expansion. In fact, since the high ratio of deleterious-to-neutral mutations decreased with further breeding (Fig. 3c, Supplementary Fig. 7a–c and Supplementary Table 17), such efforts may have used small populations purged for deleterious alleles through inbreeding. Hybridization with local wild populations from Guangxi and Guangdong may have occurred serendipitously, with heterosis progressing by selecting admixed cultivars with higher quality fruits. Finally, Hainan cultivars further interbred with wild material, as indicated by some third-degree relationships to wild individuals; indeed, similar recurrent introgression has been observed in other species^11^. Among wild populations, YNW had significantly lower π_n_ /π_s_ ratio (Fig. 3c and Supplementary Fig. 7d), suggesting deleterious mutations were purged through inbreeding. Thus, we infer that historical lychee breeding progressed by selecting distinct lines with increased heterosis, with the most recent cultivars showing greatest genetic diversity.

### Annotation of lychee haplotypes

**Extended Data Fig. 7:**
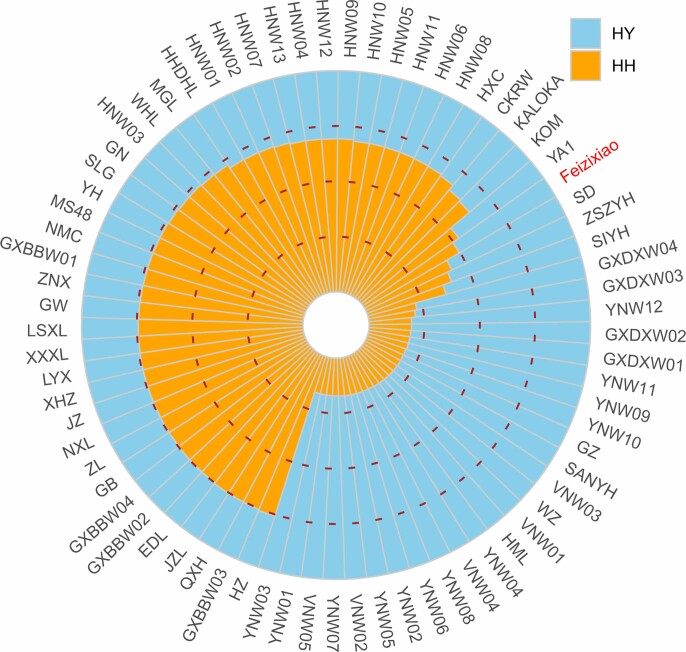
Mapping ratios to haplotypes HY and HH for different lychee accessions. The LMC/HNW group were preferentially aligned to HH, while EEMC/YNW to HY with mapping ratio of 64.0%-75.8% and 77.7%-83.1%, respectively. Three red dashed circles represent 25%, 50% and 75%, respectively.

The high heterozygosity (2.27%) of ‘Feizixiao’ lychee also enabled us to generate two haplotypes using SNP phasing (with HapCUT2 (ref. ^12^)) combined with single-cell 10x Genomics sequencing (100× coverage) to obtain 15 pairs of homologous chromosomes each (Extended Data Fig. 1, Supplementary Fig. 8 and Supplementary Tables 18–20). When resequencing data from different accessions were aligned to these 30 haplotypic chromosomes, coverage differences were observed; that is, shotgun reads of the EEMC/YNW group aligned preferentially to one chromosome from each homologous pair, whereas reads from the LMC/HNW group (Supplementary Table 21) preferentially mapped to the other. We referred to the first set of 15 chromosomes with higher EEMC/YNW mapping ratio as Haplotype Yunnan (HY), and the remaining chromosomes as Haplotype Hainan (HH). For all LMC/HNW accessions, 64.0%–75.8% of reads mapped to HH, whereas 77.7%–83.1% reads from all EEMC/YNW accessions aligned to HY (Extended Data Fig. 7). Five EMCs, including ‘Feizixiao’, had mapping coverages (36.0–59.2%) comparable with those of both HY and HH (Extended Data Fig. 7), further supporting a possible F1 hybridization between LMC/HNW and EEMC/YNW individuals, or descendent status from such hybridization event(s).

Great variation, such as structural variants (SVs) and gene copy number variants (CNVs), were found between these two haplotypes (Supplementary Fig. 9, Supplementary Tables 22 and 23 and Supplementary Note I). Compared with the reference genome, 77.6% (24,741/31,896) and 77.1% (24,593/31,896) of HY and HH genes contained SNPs or indels, respectively. Among them, 93.6% (23,166/24,741) of HY and 93.3% (22,953/24,593) of HH genes held amino acid changes from nonsynonymous SNPs or indels. Nonsense SNPs/indels between the two haplotypes accounted for ~2.6% (8,292/319,125) of all nonsynonymous SNPs and ~9.2% (2,934/31,896) of all annotated genes. Surprisingly, 83.6% (26,672/31,896) showed amino acid differences between haplotypes (Supplementary Tables 24 and 25). This difference was reflected in the mutational load of the populations, wherein YNW and Daxin individuals displayed fewer disruptive SNPs, HNW and EEMC individuals intermediate values and EMC plus admixed cultivars the most (Supplementary Fig. 10), as also observed in π_n_ values. Average heterozygosity (2.38%) from total SNPs between HY and HH was similar to the k-mer estimate (2.27%), supporting the accuracy of haplotype assignment (Supplementary Table 18).

### Differential expression of alleles in lychee

**Fig. 4:**
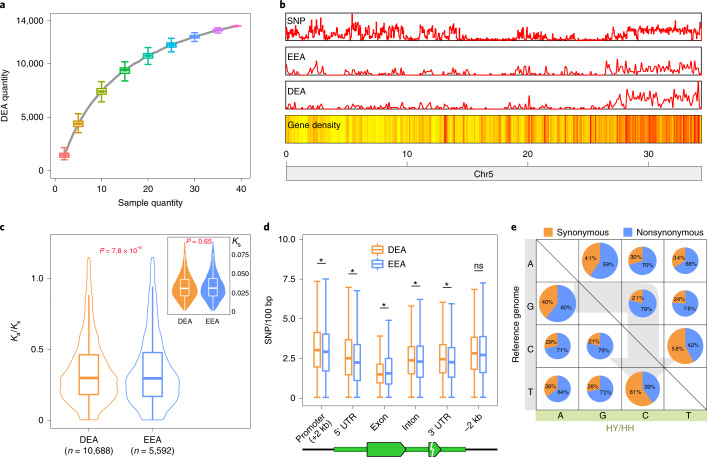
DEAs in lychee. **a**, DEA numbers increase with the quantity of RNA-seq libraries. The specified number sets were selected randomly from 39 DEA sets with 500 replicates. **b**, DEAs are unevenly distributed in the ‘Feizixiao’ genome. The gene density is represented by a yellow-to-red color scheme, with a redder color denoting a higher level of gene density. **c**, DEAs are of relatively lower *K*_a_/*K*_s_ value. Minima and maxima are present in the lower and upper bounds of the whiskers, respectively, and the width of violin are densities of *K*_s_ or *K*_a_/*K*_s_ value. *P* values were calculated with two-sided Student’s *t*-test. Numbers of genes: *n* = 10,688 (DEA); *n* = 5,592 (EEA). *P* = 0.65 for *K*_s_, *P* = 7.8 × 10^−6^ for *K*_a_/*K*_s_. **d**, SNP density in gene features. The *y* axis represents SNP numbers every 100 bp. The asterisk indicates significance with two-sided Student’s *t*-test. The *P* values for promoter, 5′ UTR, exon, intron, 3′ UTR and −2 kb (2 kb sequence downstream of 3′ UTR) are *P* = 1.5 × 10^−4^, *P* = 2.0 × 10^−15^, *P* = 2.1 × 10^−38^, *P* = 0.047, *P* = 5.4 × 10^−8^ and *P* = 0.12, respectively, and their gene numbers of DEA versus EEA are *n* = 14244 versus *n* = 8679, *n* = 10312 versus *n* = 4472, *n* = 14244 versus *n* = 8679, *n* = 12426 versus *n* = 7569, *n* = 10284 versus *n* = 4463 and *n* = 14244 versus *n* = 8679, respectively. ns, not significant. **e**, The numbers of base pair transformations and their synonymous and nonsynonymous substitution rates. In **a**, **c** and **d**, box plots show the median, box edges represent the 25th and 75th percentiles and whiskers represent the maximum and minimum data points within 1.5× interquartile range outside box edges.

Correlated or differential expression of alleles could have profound effects on growth and evolvability^13,14^. The high heterozygosity of ‘Feizixiao’ enabled us to distinguish alleles and study hybrid vigor using distinct SNPs. We found that numbers of differentially expressed alleles (DEAs) increased log-linearly with sample quantity, plateauing at ~14,000 DEAs with over 35 samples (Fig. 4a, Supplementary Fig. 11a and Supplementary Table 26). Totally, 13,517 DEAs were identified in ‘Feizixiao’ (Supplementary Table 27). These DEAs were specifically enriched in certain genomic regions, for example, many DEAs amassed at the 3′ terminus of chromosome 5 (Fig. 4b and Supplementary Fig. 11b).

To assess possible natural selection on allelic gene expression, we calculated *K*_a_ and *K*_s_ values between allelic gene pairs. Both *K*_a_ and *K*_s_ were low (<0.05) for most alleles, indicating strong identity between allelic genes (Supplementary Fig. 12). However, DEAs had significantly lower *K*_a_/*K*_s_ than equivalently expressed alleles (EEAs) (*t*-test, *P* value = 7.8 × 10^−6^), indicating that DEAs were under greater purifying selection pressure (Fig. 4c). About 5.7% (1,824 out of 31,896) of allelic genes evidently experienced purifying selection (*K*_a_/*K*_s_ <0.1), while 3.7% (1,186 out of 31,896) of allelic pairs showed possible positive selection (*K*_a_/*K*_s_ >1) (Supplementary Table 28).

Compared with EEAs, the promoters, introns and 3′ and 5′ untranslated regions (3′ UTR, 5′ UTR) of DEAs had much higher SNP densities, suggesting that their differential expression may reflect different binding affinities of transcription factors to promoter regions (Fig. 4d). SNP density in exons was significantly lower than elsewhere (for example, exons versus promoters showed a 1.47-fold difference), indicating greater purifying selection pressure on functionally constrained protein-coding regions (Fig. 4d). For these exonic SNPs, transitions were more prevalent than transversions, with most being nonsynonymous (Fig. 4e and Supplementary Tables 29 and 30).

### Flowering-related genes in lychee

**Extended Data Fig. 8:**
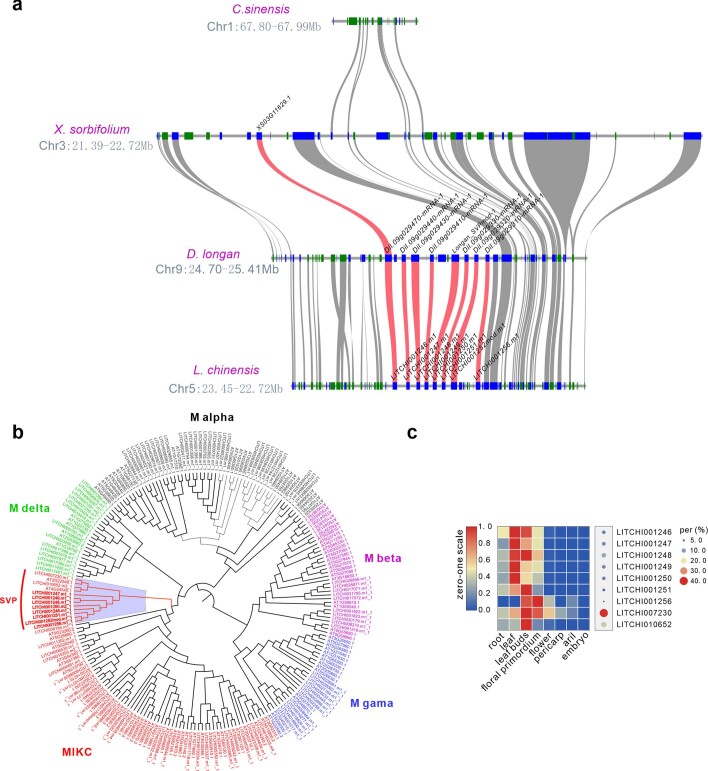
The *SVP* gene cluster identified in Sapindaceae. a. Intergenomic synteny among *C. sinensis*, *X. sorbifolium*, *D. longan* and *L. chinensis*. b. Phylogenetic tree of *MADS-box* genes from lychee and *A. thaliana*. Different classes of *MADS-box* genes are indicated with different colors. c. Expression profiles of *SVP* genes in lychee.

Flowering time, and number of days from flowering to fruit harvest, are the two key traits marking lychee fruit maturation. For most cultivars, flowering to fruiting ontogeny is similar in number of days. Therefore, flowering time is the main determinant of fruit maturation. We obtained a list of 501 lychee homologs to relevant flowering-related genes from model species, and many families of these genes have expanded in lychee (Supplementary Tables 31 and 32), suggesting the possibility of a complex network of flowering regulation. On chromosome 5, there is a closely arrayed cluster of MADS-box genes that probably arose from tandem or segmental duplications (Extended Data Fig. 8a). These genes showed greatest similarity to the *SHORT VEGETATIVE PHASE* (*SVP*) gene of Arabidopsis, which controls flowering time by negatively regulating the expression of *FT* via direct binding to the CArG motifs in its promoter^15^. Eight out of ten lychee SVP homologs are arranged in tandem (Extended Data Fig. 8b), and showed preferential expression in leaf or leaf buds similar to Arabidopsis *SVP* (Extended Data Fig. 8c and Supplementary Note I), implying a conserved role in flowering regulation. This *SVP*-like cluster also occurs in longan (*D. longan*), but is absent in yellowhorn (*X. sorbifolium*) (Extended Data Fig. 8a), indicating derivation from duplications that occurred after lychee and longan split from their common ancestor with yellowhorn.

### A eudicot-conserved Sapindaceae-expanded *VRN1* gene cluster

**Fig. 5:**
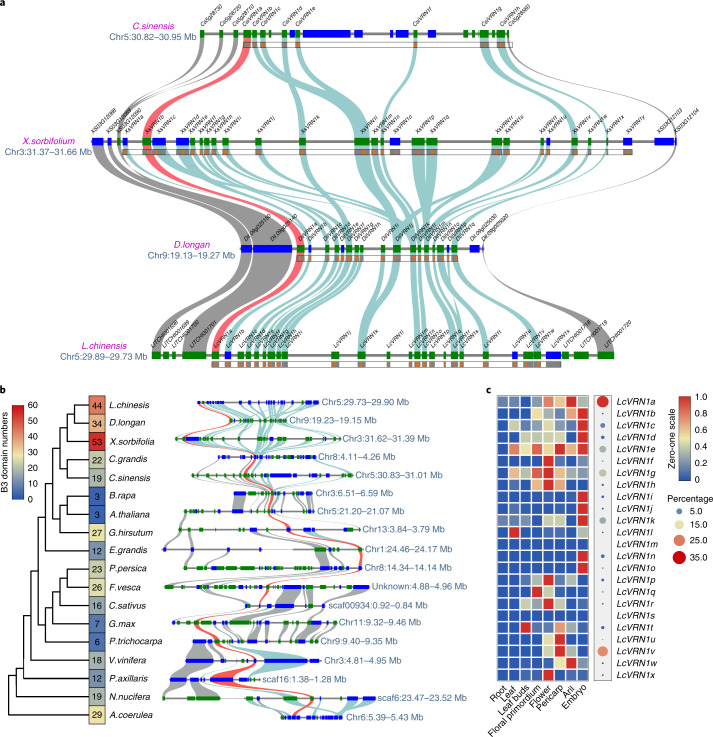
The VRN1-like gene cluster is expanded in Sapindaceae. **a**, Syntenic relationships of the *VRN1*-like gene cluster in four Sapindales species. Syntenic gene pairs are connected by curves of different colors. Red, syntenic homologs of Arabidopsis *VRN1*; cyan, *VRN1-like* genes, gray, other genes. **b**, The syntenic block containing the *VRN1*-like gene cluster in 18 core eudicot species. The numbers of B3 domains within respective clusters are indicated in the phylogenetic tree (left). Arabidopsis *VRN1* and its syntenic homologs are highlighted in red in the synteny blocks (right). **c**, Expression profiles for the 24 *VRN1*-like genes in lychee.

In addition to MADS-box genes, B3-domain-containing genes comprise another class of transcription factors tightly associated with flowering regulation^16^. Arabidopsis *VERNALIZATION 1* (*VRN1*), a member of this class, maintains vernalization via repressing *FLOWERING LOCUS C* (*FLC*) to promote flowering^17^. In the lychee genome, we identified a tandem cluster of 24 B3-domain-containing genes located within a ~170 (169.6) kb region of chromosome 5, each containing 1–3 B3 domains (Fig. 5a). We termed this the ‘VRN1 cluster’, and the genes *VRN1*-like genes. This cluster is conserved in Sapindaceae but with variable numbers of genes and corresponding B3 domains (Fig. 5a). Compared with 24 genes with 44 B3 domains in lychee, there are 26 genes with 53 B3 domains and 17 genes with 34 B3 domains in yellowhorn and longan, respectively (Fig. 5a). We also found a syntenic *VRN1*-like cluster in orange (*C. sinensis*)—a species from the same order, Sapindales, wherein the 129.25 kb region contains many fewer *VRN1*-like genes (only 7) and B3 domains (only 14) (Fig. 5a).

**Extended Data Fig. 9:**
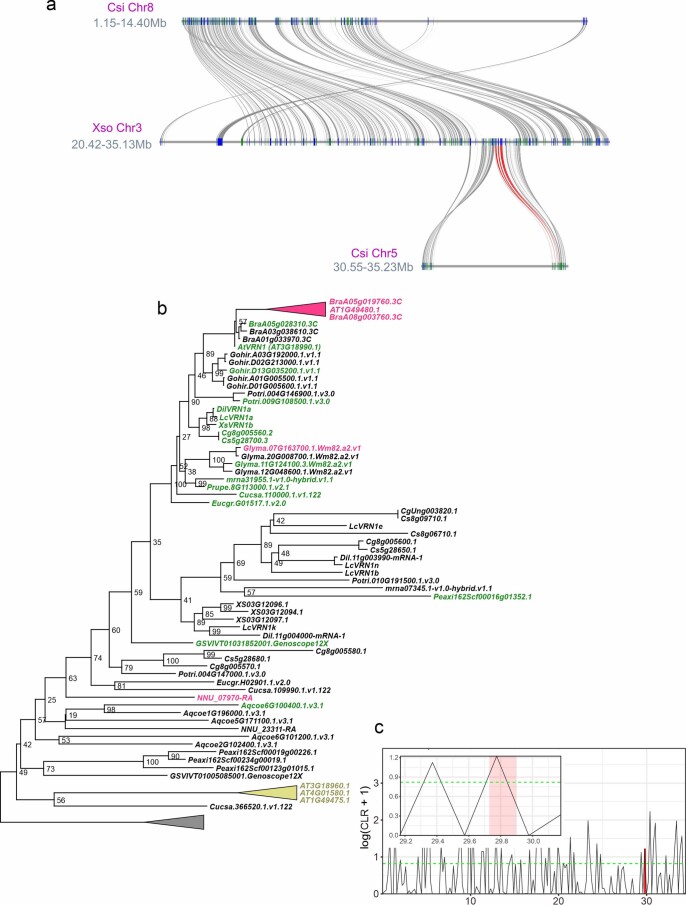
A eudicot-conserved syntenic VRN1 gene cluster is expanded in Sapindaceae species. a. Synteny between C. sinensis and X. sorbifolium. with *VRN1*-like genes connected by red curves. b. A ML tree (IQtree with JTT + F + I + G4 model) of VRN1 homologs among 18 core eudicot species. Green: VRN1 homologs; magenta triangle: REM19 or RTVRN homologs; yellowish-brown triangle: REM16 homologs; gray triangle: outgroup genes. c. VRN1 cluster located in a sweep region was identified by SweepFinder2 using 200 kb windows. Dashed line represents the 5% significance level with neutral simulations.

Intriguingly, a broader syntenic analysis revealed that the VRN1 cluster occurred in highly conserved regions in plant genomes that maintain strong syntenic relationships across almost all core eudicots (Fig. 5b). Counterparts of Arabidopsis *VRN1* were found within these syntenic blocks, as expected (Fig. 5b and Extended Data Fig. 9a,b), suggesting conserved or similar functions. Despite this high conservation, numbers of *VRN1-*like genes and B3 domains varied greatly among core eudicot species. There were only a few B3 domains within the VRN1 cluster in some plants, including Arabidopsis, tomato and soybean (Fig. 5b), while large expansions were detected in all Sapindaceae species (Fig. 5b), implying possible greater functional complexity.

Next, we examined expression of lychee *VRN1-*like genes. Overall, the 24 genes displayed different expression patterns (Fig. 5c), with *LcVRN1a*, *Lc-VRN1e*, *Lc-VRN1g* and *Lc-VRN1v* being the predominantly expressed duplicates. Many of the other *VRN1-*like genes showed little expression (Fig. 5c), suggesting functional divergence after duplication. Intriguingly, this VRN1 cluster in lychee was located in a sweep region of potential positive selection (Extended Data Fig. 9c and Supplementary Note I).

### A *CONSTANS*-like gene pair contributes to fruit maturity

**Fig. 6:**
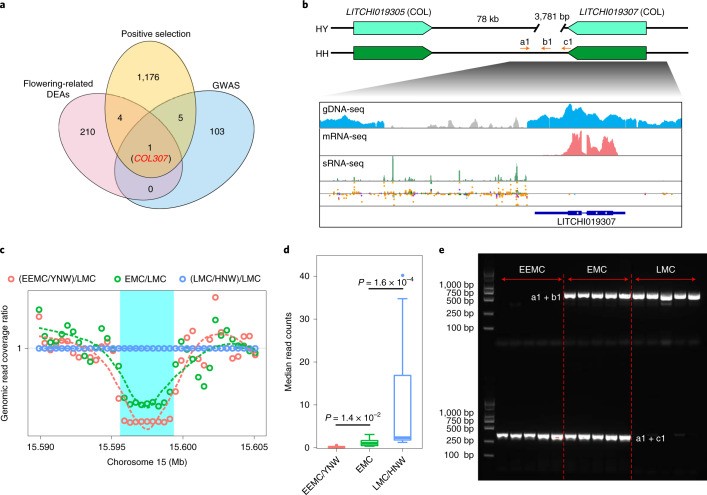
A pair of *COL* genes associated with lychee fruit maturation time. **a**, Venn diagram of positively selected genes, genes identified from the GWAS analysis and flowering-related DEA genes. **b**, Identification of a heterozygous 3.7-kb deletion downstream of the *COL307* in the ‘Feizixiao’ genome. gDNA coverage over the deletion region is in gray. sRNA data are presented by either coverage in green or color-coded dots. Orange dots denote 24-nt siRNAs. Positions of PCR primers used for genotyping are labeled a1, b1, and c1. **c**, Lower coverage of genomic sequencing reads over the 3.7 kb region in EEMC/YNW and EMC cultivars suggests a deletion (light blue box). Open circles reflect average genomic coverage in 800 bp windows across chromosome 15. **d**, The coverage over the deletion region is significantly lower in the EEMC/YNW and EMC cultivars than in the LMC/HNW cultivars. *P* values were calculated with two-sided Student’s *t*-test. Numbers of accessions: *n* = 8 (EMC), *n* = 20 (EEMC/YNW) and *n* = 36 (LMC/HNW). Box plots show the median, box edges represent the 25th and 75th percentiles and whiskers represent the maximum and minimum data points within 1.5× interquartile range outside box edges. **e**, The 3.7-kb deletion can be used as a molecular marker to distinguish accessions from the EEMC, EMC and LMC cultivar groups. The PCR amplification was performed once.

**Extended Data Fig. 10:**
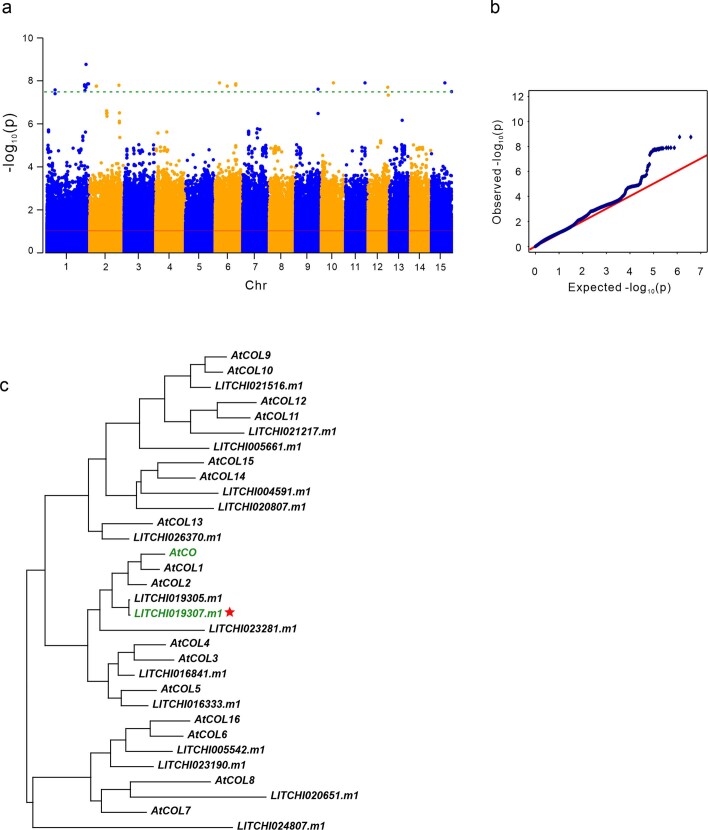
A pair of *CONSTANS* genes contributes to fruit maturation time in lychee. a. Manhattan plot of the GWAS analysis based on early maturing vs. late-maturing accessions. The green dashed line represents 5% Bonferroni-corrected significance. b. A quantile-quantile plot of the GWAS analysis. c. A ML (FastTree) phylogeny of *CONSTANS*-like genes in lychee and Arabidopsis. *COL305* and *COL307* group near the *AtCO* gene.

To further dissect the regulatory network of lychee fruit maturation, we conducted a genome-wide association study (GWAS) using our 72 accessions (Extended Data Fig. 10a,b and Supplementary Table 33). One gene was identified as flowering-related and probably positively selected (*K*_a_/*K*_s_ =1.43) during lychee domestication (Fig. 6a). This gene (*LITCHI019307*) encodes a CONSTANS-like (COL) protein—a transcription factor whose Arabidopsis homolog mediates the circadian clock and flowering control^18^. Allelic variants of this gene (subsequently referred to as *COL307*) were differentially expressed, with the HH allele showing greater expression. This pattern correlates well with the fact that maturation time of ‘Feizixiao’ lychee is intermediate between EEMC (HY) and LMC (HH) accessions. Among HY/HH *COL307* variants, a heterozygous deletion of 3,781 bp exists in the 3′ region of HY only (Fig. 6b). Both the EEMC/YNW and LMC/HNW groups are otherwise homologous in this region, with the former having the deletion in both haplotypes, the latter not. Genomic read coverage was significantly lower in the EEMC/YNW and EMC group than in the LMC/HNW group (Fig. 6c,d). The 3.7 kb deletion is probably part of a long terminal repeat (LTR) retrotransposon, which can generate profuse 24-nt siRNAs and regulate the expression of adjacent genes possibly via mediation of DNA methylation. This observation suggests that the 3.7 kb deletion may contribute to *COL307* differential expression and flowering time differences among lychee accessions. Based on this deletion, we designed three PCR primers to specifically distinguish the EEMC/YNW, EMC and LMC/HNW lychee groups (Fig. 6e), establishing the deletion as a useful molecular marker for breeding varieties with different fruit maturation times. Additionally, we found another *CO*-like gene (*COL305*) ~100 kb away from *COL307* (Fig. 6b) with high sequence similarity (Extended Data Fig. 10c and Supplementary Fig. 13) and similar expression patterns. We speculate that *COL305* may also contribute to flowering time regulation together with *COL307*.

## Discussion

It is unusual that two genomic haplotypes are traceable to entirely different ancestral populations, since recombination normally mixes such distinct variation over time. The only plausible explanation is that ‘Feizixiao’ lychee is a recent, or even F1, hybrid between cultivars homozygous for the HY and HH haplotypes, and that it has been propagated vegetatively ever since. Such propagation is a common procedure in lychee cultivation worldwide to preserve uniformity and fruit quality of elite cultivars.

We speculate that lychee might have originated in Yunnan, then spreading to Guangxi and Guangdong before finally arriving in Hainan, since lychee seeds are often too large to be animal-dispersed in the wild; instead, they are distributed mainly by river. The Xijiang River is the main channel of the Pearl River, which originates in the Maxiong Mountains in Zhanyi County in northeast Yunnan, whereafter it runs through Yunnan, Guangxi and Guangdong. This river is known as the ‘Golden Waterway’ that connects South China to Southwest China^19^. Wild lychee germplasm from Yunnan, Vietnam and Daxin County (Guangxi) versus that from Hainan and Bobai County (also Guangxi) reflected two ancestral populations, albeit with geographic linkage. Conceivably, YNW lychee spread downstream along the Xijiang River, resulting in the GXW and HNW populations. Second, Bobai County and the Leizhou Peninsula of Guangdong are adjacent. The Leizhou Peninsula and Hainan Island were once connected, but were separated during the Pleistocene^20,21^. A vast region of modern Bobai, Leizhou and Hainan was affected by explosive volcanic activity^22^, leading to large-scale environmental changes in lychee habitat that might have helped drive differential evolution of HY and HH after the YNW population spread to those areas.

Population structure and phylogenetic analyses of resequenced lychee genomes indicated independent domestication events for cultivated lychee, wherein EEMC was domesticated from YNW, while LMC was domesticated from HNW in Guangdong and Guangxi, and further refined by subsequent crosses with local inbred populations. Given admixture statistics, evidence from inbreeding, relatedness and comparative mapped reads coverage between the HY and HH genomes, EMC probably originated from hybridization between EEMC and LMC parents, with LMC probably the female and EEMC the male parent. In wild populations, we found evidence for long-lasting inbreeding in YNW, resulting in demographies inconsistent with the more outbred Hainan population. We developed an ad hoc approach to compensate for this^23^, but further methods development will be necessary to formally incorporate reproductive strategy differences into modeling.

Floral induction of lychee requires a period of relatively low temperature. In the Arabidopsis vernalization response, VRN1 cooperates with other proteins to promote flowering via the induction of epigenetic silencing of the repressor gene *FLC* (ref. ^24^). Although this chilling requirement in perennial fruit trees might be different from the vernalization process in crucifers, the well-maintained synteny of *VRN1*-like genes in core eudicots implies a potentially conserved role in flowering control. Furthermore, the expansion of the *VRN1*-like gene cluster in Sapindaceae points toward more diversified functions of these genes in flowering (or other processes) within this family. The Arabidopsis CO protein is a key player in the photoperiod pathway, which controls flowering in response to seasonal day length changes^25^. A polymorphic 3.7 kb deletion near a lychee *CO*-like gene (*COL307*) is strongly associated with maturation times of different lychee varieties, providing an ideal target for molecular breeding and genomic selection for more diversified fruit maturity.

## Methods

### Genome sequencing

#### Sampling

The lychee cultivar ‘Feizixiao’ is a prevalent lychee cultivar in China and the main cultivar traded on the world market. Therefore, we chose ‘Feizixiao’ for full-genome sequencing.

#### Illumina short-reads sequencing

DNA was extracted from leaf tissue using a Qiagen DNeasy Plant Mini Kit, and a 270-bp paired-end library was generated using an NEBNext Ultra DNA Library Prep Kit. Sequencing was performed using Illumina HiSeq2500 and Hiseq4000 platforms. In total, 30.65 Gb (~66×) of data were obtained, and the reads were trimmed using Trimmomatic^30^ (v.0.36) with default parameters.

#### PacBio library construction and sequencing

More than 6 µg of sheared DNA was subjected to size-selection by the BluePippin system and ~20 kb Sequel SMRT bell libraries were prepared according to the protocol provided by the Pacific Biosciences Company (PacBio). Four single-molecule real-time (SMRT) cells were run on a PacBio RSII system using P6-C4 chemistry, with ~58.6 Gb (~124×) long-read data generated.

#### Hi-C library construction and sequencing

A Hi-C library was constructed from young leaves of ‘Feizixiao’ by the BioMarker Technologies Company (Beijing, China) as described elsewhere^31^. A total of 232 million (~144×) 150 bp paired-end reads were produced on the Illumina HiSeq ×10 platform.

### Genome assembly

#### Reference genome assembly

A flow chart of our genome assembly approach is shown in Extended Data Fig. 1. Briefly, clean PacBio subreads were corrected by LoRDEC^32^ (v.0.7) with short reads. Subsequently, corrected long reads were set as input for Canu^33^ (v.1.6) with the parameter ‘correctedErrorRate=0.035’. The initial assembly obtained was twice the size of the anticipated genome size, suggesting that it contained two haplotypes. We next used HaploMerger2 (ref. ^34^) (release 20180603) to extract two haplotypes with parameters ‘minOverlap=99999999’ in B3 stages, otherwise using default settings. The one haplotypic assembly (**HM_ctg2**) closer to the expected genome size was anchored into 15 pesudochromosomes (reference genome, marked as Ref) using Juicer^35^ (v.1.6.2) and 3D-DNA^36^ in combination with the Hi-C reads.

#### Hi-C heatmap

Right and left reads of Hi-C data were mapped separately to the reference genome with parameters ‘-A1 -B4 -E50 -L0’ using BWA^37^ mem (v.0.7.17), and the Hi-C contact matrix was calculated and plotted with HiCExplorer^38^ (v.2.1.1).

#### Haplotype phasing

To obtain accurate haplotypes, short reads were mapped against the reference genome using BWA^37^ mem, and the resulting alignment file was duplication-marked and sorted using Samtools^39^. The sorted alignment file was then used for variant calling using the Genome Analysis ToolKit (GATK) (v.4.1.4.1) pipeline^40^. The variants passing the hard-filter were kept with criteria ‘QD < 2.0 || MQ < 26.0 || FS > 100.0 || SOR > 5.0 || MQRankSum < −7.5 || ReadPosRankSum < −8.0’, giving rise to the ‘Feizixiao’ VCF file. Only biallelic SNPs were selected for haplotype phasing using HapCUT2 (ref. ^41^) (v.1.1), combined with Hi-C and corrected PacBio reads. We extracted the MVP blocks and filtered out both unphased SNPs and unknown genotypes. Eventually, blocks of haplotypic SNPs were retained, covering 11.2 million phased SNPs.

Meanwhile, 10x Genomics reads were mapped to reference genome sequences, and phased SNPs blocks were calling using Long Ranger (v.2.2.2)^42^. We used phased haplotypic SNPs from 10x Genomics to correct those SNPs from HapCUT2 (v.1.1)^41^ for blocks containing >1,000 phased SNPs identified in 10x Genomics. The corrected haplotypic SNPs were used for PacBio reads phasing using a method first described for sex chromosome phasing^43^. Phased reads were assembled de novo using Canu (v.1.6)^33^. Contigs were linked with the help of reference genome sequences using RaGOO (v.1.1)^44^ software.

Finally, 15 pairs of homologous pseudochromosomes were obtained. Afterwards, Illumina population resequencing reads from different lychee accessions were aligned to the 15 pseudochromosome pairs, with those dominated by reads from the YNW/EEMC/GXW cultivar group assigned to the HY haplotype, with the remaining haplotype being termed HH.

### Genome annotation

#### Gene model annotation

Gene models were annotated using the MAKER^45^ genome annotation pipeline (v.2.31.8), which integrates both ab initio gene predictions generated by AUGUSTUS^46^ (v.3.3.2) and SNAP^47^ (v.2013-11-29) and homology evidence including plant protein sequences in the SwissProt database^48^ (release 2018_12) as well as a de novo transcriptome assembly generated from lychee RNA-seq data using Trinity^49^ (v.2.8.3). For improved results, we ran the MAKER pipeline iteratively (three times in total) as recommended^45^. The gene models were further refined using PASA^50^ (v.2.3.3). For the gene models from the two haplotypes, those models corresponding to the longest transcripts in the reference were converted to their two haplotypic sequences using GMAP (v.2017-11-15)^51^ with parameter ‘*n* = 2’. Haplotypic gene models were considered redundant if the overlap between two gene models was over 60%, and these were subsequently removed from the final gene model list.

#### Completeness of gene annotation

BUSCO^52^ (v.3.0.1) was used for evaluation of annotation completeness with the ‘eudicotyledons_odb10’ database.

#### LTR annotation

LTRs were predicted using LTR_FINDER^53^ (v.1.07) with parameters ‘-D 15000 -d 1000 -L 7000 -l 100 -p 20 -C -M 0.9’ and with LTRharvest^54^ (v.1.5.10) using parameters ‘-similar 90 -vic 10 -seed 20 -seqids yes -minlenltr 100 -maxlenltr 7000 -mintsd 4 -maxtsd 6’. Results from the above LTR annotation steps were integrated using LTR_retriever^55^ (v.2.6), and LAI^56^ was also evaluated.

### Evolutionary analyses

#### Gene family identification

Genome assemblies and GFF3 files for 12 species, including *D. longan*^57^, *X. sorbifolium*^58^, *C. sinensis*^59^, *Arabidopsis thaliana*^60^, *Carica papaya*^61^, *Fragaria vesca*^62^, *Malus domestica*^63^, *Cucumis sativus*^64^, *Medicago sativa*^65^, *Vitis vinifera*^6^, *Solanum lycopersicum*^66^ and *Acer yangbiense*^67^, were downloaded from public databases (Supplementary Table 34). Protein-coding sequences from 13 species were exacted using TBtools^68^, and only the longest transcripts were retained. Gene family clusters and single-copy ortholog sequences within the protein set were identified using Orthofinder2 (ref. ^69^) (v.2.3.3).

#### Evaluation of divergence time

Twenty single-copy orthologous protein sequences were used to construct the phylogenetic tree using MCMCtree^70^ (v.4.9i) with two calibration points (Fig. 1c, red diamond represents Rutaceae fossil record^26^; the blue diamond represents *S. lycopersicum*^66^ divergence time^27^). We adopted the Bayesian relaxed molecular clock approach to estimate species divergence time using the independent molecular clock and Empirical+F models with default ‘wag.dat’ amino acid substitution rate in the program MCMCtree^70^, with other parameters set to defaults.

#### Calculation of *K*_a_ and *K*_s_

Paralogous and orthologous gene pairs were identified in syntenic blocks using the MCScanX^71^ software, while *K*_a_ and *K*_s_ were calculated in TBtools^68^ using coding and protein sequences. We then filtered out zero values and plotted the data with the ggplot2 (ref. ^72^) package.

#### Circos

Gene density was calculated directly from the ‘Feizixiao’ GFF3 file in 100-kb windows. SNP density and nucleotide diversity were calculated only for SNPs extracted from the ‘Feizixiao’ VCF file. Similarly, sRNA and LTR densities were calculated from small RNA annotation^73^ and LTR annotation files, respectively. All the above ‘Feizixiao’ genome features were viewed in Circos plotted using TBtools^68^.

#### Dot-plot

Protein sequences from five species, *L. chinensis, D. longan*^57^*, X. sorbifolium*^58^*, C. sinensis*^59^ and *A. yangbiense*^67^, were extracted using TBtools^68^ and viewed in JCVI^74^ (v.0.8.4) with a parameter setting of ‘–cscore = .99’.

### Resequencing

#### Illumina short-reads sequencing

DNAs from leaf tissue of 72 lychee accessions were used for the construction of 450-bp paired-end libraries at BGI (Shenzhen, China), and were then sequenced using the Illumina HiSeq2000 platform. In total, 405.4 Gb (average 13×) of data were obtained.

#### Mapping ratio to HY and HH

After initial quality control using FastQC^75^ and adapter trimming using Trimmomatic^30^ (v.0.36, with parameters ‘LEADING:20 TRAILING:20 SLIDINGWINDOW:4:15 MINLEN:36’), the reads were mapped to the reference genome using Bowtie2 (ref. ^76^) (v.2.3.5) with parameters ‘–no-mixed–no-unal’. Reads were then counted using Samtools^39^ at the whole-genome level and compared between the HY and HH haplotypes.

#### Alignment and SNP-calling

Resequencing data for the 72 accessions were mapped to the reference genome using BWA^37^ mem (v.0.7.17). The mapped reads were then sorted according to genomic coordinates using Samtools^39^. Sequence data generated from different Illumina lanes were combined using ‘samtools merge’. After merging, duplicates were removed using Picard^77^ (v.2.5.0), and then HaplotypeCaller from the GATK^40^ (v.3.8) was used to call individual-specific gvcf files. Finally, the GenotypeGVCFs was used for joint calling of SNPs. After quality control of the SNPs using bcftools^78^, the SNPs were hard filtered using GATK VariantFiltration (DP < 300 || DP > 3000 || QD < 2.0 || FS > 60.0 || MQ < 40.0 || MQRankSum < −12.5 || ReadPosRankSum < −8.0), and only biallelic SNPs were selected for further analysis.

#### Admixture

We carried out analysis of admixture using the ADMIXTURE^9^ software (v.1.3.0). Before analysis, SNPs in LD were dropped out in Plink^79^ using a sliding window of 50 SNPs with 10 SNP step size, SNPs with *R*^2^ >0.1 were filtered out, these settings were the same as recommended in the ADMIXTURE user manual (–indep-pairwise 50 10 0.1). The optimal number of populations (*K*) was selected using tenfold cross-validation.

#### SNP tree

The data filtered for ADMIXTURE analysis were also used for estimating the population SNP tree with SNPhylo^80^ (v.20180901). The SNPs were filtered by >10% minor allele frequency (-m 0.1) and missing rate <40% (-M 0.4) and *R*^2^ <0.4 (-l 0.4). The resulting fasta file was used as input to RAxML^81^ (v.8.2.12), where the tree was run using the GTR-GAMMA model and 1,000 bootstrap replicates, and thereafter rooted by the outgroup species.

#### Overall population statistics

Population statistics were calculated using Plink^79^ (v,1.90p). PCA was calculated using SNPs where genotypes were called for over 80% of the individuals. Identity-by-state analysis was carried out in Plink using the ‘–genome full’ option, and inbreeding coefficient with ‘–het’.

### Population history

#### Ancestral state estimation and unfolded site frequency spectrum

The ancestral states were obtained by aligning the outgroup species longli, longan and rambutan against the haploid lychee reference assembly using BWA mem (v.0.7.17). The ancestral allele was called using a majority vote in ANGSD^82^ (v.0.933) with -doFasta 2 -doCounts 1 options. The ancestral states were set as reference in the .vcf file using bcftools fixref, and the ancestral state was also incorporated in the .vcf file information field with bcftools annotate.

#### SMC++

The .vcf file was converted into SMC++ (ref. ^28^) (v.1.15.3) format using vcf2smc; the repeat regions annotated with RepeatModeler and the SNPs with no ancestral state prediction were filtered out. An unfolded composite likelihood using HNW13 and HNW03 individuals as distinguished samples was estimated for the Hainan population; for the Yunnan population, YNW01 and YNW05 individuals were used. These four individuals had the highest coverage among the populations under inspection. The model parameters for SMC++ (ref. ^28^) (v.1.15.3) were obtained with threefold cross-validation after fixing the estimated time interval to 10 to 200,000 generations and by varying the mutation rate estimates, as described by Salojarvi et al.^83^. The population split time was estimated with smc++ split. The predicted demography was produced using smc++ plot and then visualized in R^84^ (v.3.6.0) using the ggplot2 (ref. ^72^) package.

#### Stairway plots

For input data, the .vcf file containing SNPs with ancestral allele calls was filtered for Yunnan and Hainan subpopulations and repeat regions were removed using vcftools. The derived site frequency spectrum was obtained with ANGSD^82^ (v.0.933) and used as input for Stairway plot^85^ (v.2.1) with 200 bootstrap estimates.

#### Pairwise sequentially Markovian coalescent model

The pairwise sequentially Markovian coalescent model^86^ was estimated from read mappings where the repeat regions were masked out. Standard parameter settings (N25 -t15 -r5 -p 4 + 25*2 + 4 + 6) were used in estimating the population history.

#### Split time estimation with Fastsimcoal2

Population split time and the presence of subsequent bottlenecks was assessed with Fastsimcoal^87^ (v.2.6.0.3). The two-dimensional derived allele site frequency spectrum was obtained with easySFS (https://github.com/isaacovercast/easySFS), using a .vcf file filtered for repeat regions and sites with no ancestral allele call. A projection to 22 samples was found optimal. In fastsimcoal^87^ (v.2.6.0.3), three models were compared: (1) a model with constant population sizes and a single split event fitted to data; (2) a model with constant population sizes, with bottlenecks in each of the populations and split time estimated from data; and (3) a model with population growth rates, bottlenecks and split time estimated from data. For each of the models, 100 parameter files were simulated. For each parameter file, 1,000,000 simulations were run; monomorphic sites were not used. Maximum composite likelihood estimation of parameters was carried out with 40 expectation-conditional maximization iterations. The best model (3) was selected using Akaike information criterion.

#### Effect of inbreeding on population trajectories

To correct the differences in population trajectories due to higher level of inbreeding Yunnan population, the generation time was adjusted to three times the generation time in the Hainan population (upplementary Note II). To avoid overfitting, matching was made in pairwise sequentially Markovian coalescent plots and the effect was then confirmed in Stairway plots and SMC++ analyses. To estimate new split times, independent SMC++ models for Yunnan and Hainan populations were fitted and the generation times were adjusted according to the method described in Supplementary Note II. The split time was then estimated manually from the matched Ne trajectories.

#### Kinship inference

The KING^29^ software (kinship-based inference for GWAS, v.2.2.4) was used to estimate kinship among individuals. Relatedness was estimated using the ‘–related’ option and default settings. The method calculates an estimator of the kinship coefficient that is independent of sample composition or population structure based on the difference between shared heterozygosity and shared homozygosity^29^. The order of relationship was obtained using the ranges recommended in KING user manual, and compared with identity-by-state values calculated with plink. The detected relationships were then illustrated using Apple Keynote.

#### Linkage disequilibrium

For LD analysis, the .vcf file was filtered by dropping out the outgroup species and by selecting SNPs present in all individuals and having minor allele count of greater than four. LD was calculated for a 500 kb window, and all *R*^2^ values >0.01 were reported. In R, a linear model was applied to the data using the ‘lm’ function with log_10_ transformed distance versus *R*^2^ value data, and the point where *R*^2^ dropped to 50% of the initial value was reported as the measure of decay. LD levels in wild and cultivated population groups were calculated by PopLDdecay^88^ (3.40) software using the correlation coefficient (*r*^2^) of alleles.

#### Introgression statistics

F3 statistics were calculated using the Admixtools^10^ package (v.4.1). The Plink formatted file was first converted into eigenstrat format using the ‘convertf’ function, and then qp3Pop was used to estimate the F3 introgression statistics. The wild individuals were grouped into populations according to sampling location, whereas the cultivars were analyzed separately. Visualization and false discovery rate adjustment of the Z-score statistics were done using R, as in Salojarvi et al.^83^.

#### F_st_ estimates

F_st_ estimation was carried out using vcftools^89^ (v.0.1.15) by grouping the wild populations according to sampling site, and cultivars according to maturation time. F_st_ was estimated in windows of 100 kb, and the average of the Weir and Cockerham weighted F_st_ values^90^ was calculated. Nucleotide diversity was estimated for each of the populations, and for all individuals together, using vcftools in 100-kb windows with a 10-kb step size.

#### π_n_ and π_s_ estimation

We developed a custom pipeline for assessing the π_n_ and π_s_ statistics from whole-genome sequencing data. First, for each population to be assessed, the .vcf file containing all individuals was filtered to contain only the members of the population, and ANGSD^82^ (v.0.933) was used to calculate sitewise nucleotide diversity. Then, the impacts of SNPs were predicted with SnpEff^91^ (v.4.3t).

For π_n_ estimation, all predicted gene models were scanned and only high-quality predictions containing a methionine as the first amino acid and where the total sum length of the exons was divisible by three were selected. The positions with nonsynonymous changes inside high-quality gene models were then selected based on ‘high’ or ‘moderate’ SNP effect annotation using SnpEff^91^ (v.4.3t). The nucleotide diversities in the identified positions were then summed together using a custom script in R with a parallel implementation for higher speed. In the presence of missing data, the mean nucleotide diversity estimate is heavily affected by the number of called SNP positions. To obtain this number, GATK^40^ (v.3.8) GenotypeGVCFs was run by also calling monomorphic positions, and the resulting .vcf files were filtered using the Phred quality thresholds used for filtering the .vcf file containing only variant positions in vcftools. For the π_n_ estimate the number of called positions inside high-quality gene model regions was then calculated. We operationally assumed that every third position in the exons caused a synonymous mutation and therefore multiplied the number of nucleotide calls inside high-quality gene models by two-thirds.

For π_s_ estimation, all regions with predicted gene models were filtered out from the ANGSD^82^ (v.0.933) file containing sitewise nucleotide diversities. To obtain the number of called positions, GATK^40^ (v.3.8) GenotypeGVCFs was run by also calling monomorphic alleles and the resulting .vcf file was filtered using the Phred quality thresholds used for filtering the .vcf file containing only variant positions. Subsequently all regions with predicted gene models were filtered out. The sums of diversities outside of gene models were then calculated using a script in R with parallel implementation for higher speed. and the sums were then divided by the number of called positions.

To verify that the values obtained from the pipeline are correct, they were compared with results from several species reported in Chen et al.^92^. All values are comparable with other plant species as well as the π_n_/π_s_ ratio. The draft pipeline containing the R codes is given in Zenodo^93^.

In addition to nucleotide diversity calculation, also the actual numbers of SNPs with different functional impact were quantified. The impacts of SNPs were first predicted using SnpEff^91^ (v.4.3t), and processed with custom made bash and R scripts. The significance of the difference between the counts for different populations was assessed in R using ANOVA and TukeyHSD post hoc analysis; ANOVA was used since the normality assumption of the residuals could not be rejected (*P* > 0.1, Shapiro test).

#### Genome-Wide Association Study

For GWAS analysis, 62 accessions had fruit maturation period recorded, which were encoded ‘1’ for extremely early maturing and early maturing accessions and ‘0’ for late-maturing accessions. Comparing with a general linear model (glm), we accepted a mixed linear model (mlm) with structure result (Q matrix, resulting from admixture analysis), and kinship together with PCA results to conduct association analysis using TASSEL^94^ (v.5.2.52).

#### Chloroplast genome assembly and genetic distance tree construction

A reference lychee chloroplast genome from previous publication^95^ was downloaded from the National Center for Biotechnology Information (NCBI). Population resequencing reads from different lychee accessions were mapped against the reference chloroplast genome using Bowtie2 (ref. ^76^) (v.2.3.5), and only those with both pair-end reads mapping were retained. We randomly selected approximately ×150 reads in depth to the reference chloroplast genome to construct new chloroplast genomes using MIRA^96^ (v.4.0.2) and MITObim^97^ (v.1.9.1) software with default parameters. The chloroplast genomes of different lychee accessions were retrieved from the last iterations and aligned using MAFFT^98^ (v.7.429). All gaps were removed using trimAl^99^ (v.1.4.rev22), and a maximum likelihood phylogenetic tree was constructed with complete chloroplast genome sequences using IQ-TREE^100^ (v.1.6.10).

Remaining method descriptions are included to Supplementary Note III.

### Reporting Summary

Further information on research design is available in the Nature Research Reporting Summary linked to this article.

## Online content

Any methods, additional references, Nature Research reporting summaries, source data, extended data, supplementary information, acknowledgements, peer review information; details of author contributions and competing interests; and statements of data and code availability are available at 10.1038/s41588-021-00971-3.

## Supplementary information

Supplementary Information Supplementary Notes I–III, Note Figs. 1–3 and Figs. 1–13.

Reporting Summary

Supplementary Table 1 Supplementary Tables 1–36 are included in this file.

Peer Review Information

## Data Availability

All raw sequencing data of DNA resequencing, 10x Genomic, Hi-C and RNA-seq are available at the NCBI database with a project ID of PRJNA747875. The monoploid reference and two haplotype assemblies are also deposited in NCBI with accession nos. JAHYJY000000000, JAIUGD00000000, and JAIUGE000000000, respectively. The assembly and annotation of monoploid and haplotype genomes were also uploaded to the Mendeley database (https://data.mendeley.com/datasets/kggzfwpdr9/1). VCF files that contain all clean SNPs were also uploaded to the Mendeley database (https://data.mendeley.com/datasets/v37bv5jt6g/1). Accession number or websites for public genomic data or sequencing data are listed in Supplementary Table 34. Source data are provided with this paper.

## Source data

Source Data Fig. 1 Unprocessed gel for Fig. 6e.

## Code availability

The pipeline of π_n_ and π_s_ estimation are homemade R scripts deposited in Zenodo^93^ (10.5281/zenodo.5136527).

## References

[1] Li C (2013). De novo assembly and characterization of fruit transcriptome in *Litchi chinensis* Sonn and analysis of differentially regulated genes in fruit in response to shading. BMC Genomics.

[2] Liu C, Mei M (2005). Classification of lychee cultivars with RAPD analysis. Acta Hortic..

[3] Liu W (2015). Identifying Litchi (*Litchi chinensis* Sonn.) cultivars and their genetic relationships using single nucleotide polymorphism (SNP) markers. PLoS ONE.

[4] VanBuren R (2011). Longli is not a hybrid of longan and lychee as revealed by genome size analysis and trichome morphology. Trop. Plant Biol..

[5] Huang S, Kang M, Xu A (2017). HaploMerger2: rebuilding both haploid sub-assemblies from high-heterozygosity diploid genome assembly. Bioinformatics.

[6] Jaillon O (2007). The grapevine genome sequence suggests ancestral hexaploidization in major angiosperm phyla. Nature.

[7] Lam H-M (2010). Resequencing of 31 wild and cultivated soybean genomes identifies patterns of genetic diversity and selection. Nat. Genet..

[8] Cao K (2014). Comparative population genomics reveals the domestication history of the peach, *Prunus persica*, and human influences on perennial fruit crops. Genome Biol..

[9] Alexander DH, Lange K (2011). Enhancements to the ADMIXTURE algorithm for individual ancestry estimation. BMC Bioinf..

[10] Patterson N (2012). Ancient admixture in human history. Genetics.

[11] Julca I (2020). Genomic evidence for recurrent genetic admixture during the domestication of Mediterranean olive trees (*Olea europaea* L.). BMC Biol..

[12] Edge P, Bafna V, Bansal V (2017). HapCUT2: robust and accurate haplotype assembly for diverse sequencing technologies. Genome Res..

[13] Combes M-C, Dereeper A, Severac D, Bertrand B, Lashermes P (2013). Contribution of subgenomes to the transcriptome and their intertwined regulation in the allopolyploid *Coffea arabica* grown at contrasted temperatures. N. Phytol..

[14] Payne JL, Wagner A (2019). The causes of evolvability and their evolution. Nat. Rev. Genet..

[15] Lee JH (2007). Role of SVP in the control of flowering time by ambient temperature in Arabidopsis. Genes Dev..

[16] Swaminathan K, Peterson K, Jack T (2008). The plant B3 superfamily. Trends Plant Sci..

[17] Levy YY, Mesnage S, Mylne JS, Gendall AR, Dean C (2002). Multiple roles of Arabidopsis VRN1 in vernalization and flowering time control. Science.

[18] Suárez-López P (2001). CONSTANS mediates between the circadian clock and the control of flowering in Arabidopsis. Nature.

[19] Lin WL (2008). Exploring on the source of Pearl River. Front. Lit..

[20] Qian, S. *Volcanic activity and magma evolution in the north of the Hainan Island*. PhD Thesis, Institute of Geology. China Earthquake Administration. (2003).

[21] Chen L, Zhang YF, Li TJ, Yang WF, Chen J (2014). Sedimentary environment and its evolution of Qiongzhou Strait and nearby seas since last ten thousand years. Earth Sci. J. China Univ. Geosci..

[22] Fan QC, Sun Q, Sui JL (2004). Periods of volcanic activity and magma evolution of Holocene in North Hainan Island. Acta Petrol. Sin..

[23] Nordborg M, Donnelly P (1997). The coalescent process with selfing. Genetics.

[24] Bäurle I, Dean C (2006). The timing of developmental transitions in plants. Cell.

[25] Andrés F, Coupland G (2012). The genetic basis of flowering responses to seasonal cues. Nat. Rev. Genet..

[26] Li H-T (2019). Origin of angiosperms and the puzzle of the Jurassic gap. Nat. Plants.

[27] Zhang L (2020). The water lily genome and the early evolution of flowering plants. Nature.

[28] Terhorst J, Kamm JA, Song YS (2017). Robust and scalable inference of population history from hundreds of unphased whole genomes. Nat. Genet..

[29] Manichaikul A (2010). Robust relationship inference in genome-wide association studies. Bioinformatics.

[30] Bolger AM, Lohse M, Usadel B (2014). Trimmomatic: a flexible trimmer for Illumina sequence data. Bioinformatics.

[31] Xie T (2015). De novo plant genome assembly based on chromatin interactions: a case study of *Arabidopsis thaliana*. Mol. Plant.

[32] Salmela L, Rivals E (2014). LoRDEC: accurate and efficient long read error correction. Bioinformatics.

[33] Koren S (2017). Canu: scalable and accurate long-read assembly via adaptive k-mer weighting and repeat separation. Genome Res..

[34] Huang S, Kang M, Xu A (2017). HaploMerger2: rebuilding both haploid sub-assemblies from high-heterozygosity diploid genome assembly. Bioinformatics.

[35] Durand NC (2016). Juicer provides a one-click system for analyzing loop-resolution Hi-C experiments. Cell Syst..

[36] Dudchenko O (2017). De novo assembly of the *Aedes aegypti* genome using Hi-C yields chromosome-length scaffolds. Science.

[37] Li H, Durbin R (2009). Fast and accurate short read alignment with Burrows–Wheeler transform. Bioinformatics.

[38] Wolff J (2018). Galaxy HiCExplorer: a web server for reproducible Hi-C data analysis, quality control and visualization. Nucleic Acids Res..

[39] Li H (2009). The sequence alignment/map format and SAMtools. Bioinformatics.

[40] Poplin, R. et al. Scaling accurate genetic variant discovery to tens of thousands of samples. Preprint at *bioRxiv*10.1101/201178 (2018).

[41] Edge P, Bafna V, Bansal V (2017). HapCUT2: robust and accurate haplotype assembly for diverse sequencing technologies. Genome Res..

[42] Marks P (2019). Resolving the full spectrum of human genome variation using linked-reads. Genome Res..

[43] Zhang X (2020). Genomes of the banyan tree and pollinator wasp provide insights into fig–wasp coevolution. Cell.

[44] Alonge M (2019). RaGOO: fast and accurate reference-guided scaffolding of draft genomes. Genome Biol..

[45] Holt C, Yandell M (2011). MAKER2: an annotation pipeline and genome-database management tool for second-generation genome projects. BMC Bioinf..

[46] Stanke M (2006). AUGUSTUS: ab initio prediction of alternative transcripts. Nucleic Acids Res..

[47] Korf I (2004). Gene finding in novel genomes. BMC Bioinf..

[48] Bairoch A, Apweiler R (2000). The SWISS-PROT protein sequence database and its supplement TrEMBL in 2000. Nucleic Acids Res..

[49] Grabherr MG (2011). Full-length transcriptome assembly from RNA-Seq data without a reference genome. Nat. Biotechnol..

[50] Haas BJ (2008). Automated eukaryotic gene structure annotation using EVidenceModeler and the program to assemble spliced alignments. Genome Biol..

[51] Wu TD, Watanabe CK (2005). GMAP: a genomic mapping and alignment program for mRNA and EST sequences. Bioinformatics.

[52] Simão FA, Waterhouse RM, Ioannidis P, Kriventseva EV, Zdobnov EM (2015). BUSCO: assessing genome assembly and annotation completeness with single-copy orthologs. Bioinformatics.

[53] Xu Z, Wang H (2007). LTR_FINDER: an efficient tool for the prediction of full-length LTR retrotransposons. Nucleic Acids Res..

[54] Ellinghaus D, Kurtz S, Willhoeft U (2008). LTRharvest, an efficient and flexible software for de novo detection of LTR retrotransposons. BMC Bioinf..

[55] Ou S, Jiang N (2018). LTR_retriever: a highly accurate and sensitive program for identification of long terminal repeat retrotransposons. Plant Physiol..

[56] Ou S, Chen J, Jiang N (2018). Assessing genome assembly quality using the LTR assembly index (LAI). Nucleic Acids Res..

[57] Lin Y (2017). Genome-wide sequencing of longan (*Dimocarpus longan* Lour.) provides insights into molecular basis of its polyphenol-rich characteristics. Gigascience.

[58] Bi, Q. et al. Pseudomolecule-level assembly of the Chinese oil tree yellowhorn (*Xanthoceras sorbifolium*) genome. *Gigascience* **8**, giz070 (2019). 10.1093/gigascience/giz070 PMC6593361 31241154

[59] Xu Q (2013). The draft genome of sweet orange (*Citrus sinensis*). Nat. Genet..

[60] Initiative TAG (2000). Analysis of the genome sequence of the flowering plant *Arabidopsis thaliana*. Nature.

[61] Ming R (2008). The draft genome of the transgenic tropical fruit tree papaya (*Carica papaya* Linnaeus). Nature.

[62] Edger, P. P. Single-molecule sequencing and optical mapping yields an improved genome of woodland strawberry (*Fragaria vesca*) with chromosome-scale contiguity. *Gigascience* **7**, gix124 (2017). 10.1093/gigascience/gix124 PMC5801600 29253147

[63] Velasco R (2010). The genome of the domesticated apple (*Malus* × *domestica* Borkh.). Nat. Genet..

[64] Li, Q. A chromosome-scale genome assembly of cucumber (Cucumis sativus L.). *Gigascience* **8**, giz072 (2019). 10.1093/gigascience/giz072 PMC6582320 31216035

[65] Tang, H. et al. An improved genome release (version Mt4.0) for the model legume *Medicago truncatula*. *BMC Genomics* **15**, 312 (2014). 10.1186/1471-2164-15-312 PMC4234490 24767513

[66] Hosmani, P. S. et al. An improved de novo assembly and annotation of the tomato reference genome using single-molecule sequencing, Hi-C proximity ligation and optical maps. Preprint at *bioRxiv*10.1101/767764 (2019).

[67] Yang, J. et al. De novo genome assembly of the endangered *Acer yangbiense*, a plant species with extremely small populations endemic to Yunnan Province, China. *Gigascience* **8**, giz085 (2019). 10.1093/gigascience/giz085 PMC6629541 31307060

[68] Chen C (2020). TBtools: an integrative toolkit developed for interactive analyses of big biological data. Mol. Plant.

[69] Emms, D.M. & Kelly, S. OrthoFinder: phylogenetic orthology inference for comparative genomics. *Genome Biol.* **20**, 238 (2019) 10.1186/s13059-019-1832-y PMC6857279 31727128

[70] Yang Z (2007). PAML 4: phylogenetic analysis by maximum likelihood. Mol. Biol. Evol..

[71] Wang Y (2012). MCScanX: a toolkit for detection and evolutionary analysis of gene synteny and collinearity. Nucleic Acids Res..

[72] Wickham, H. *ggplot2: Elegant Graphics for Data Analysis*. (Springer-Verlag, 2016).

[73] Chen, C. et al. sRNAanno—a database repository of uniformly annotated small RNAs in plants. *Hortic. Res.* **8**, 45 (2021) 10.1038/s41438-021-00480-8 PMC7917102 33642576

[74] Tang, H., Krishnakumar, V. & Li, J. jcvi: JCVI utility libraries 10.5281/zenodo.31631 (2015).

[75] Andrews, S. FastQC: a quality control tool for high throughput sequence data. (Barbraham Bioinformatics, 2010).

[76] Langmead B, Salzberg SL (2012). Fast gapped-read alignment with Bowtie 2. Nat. Methods.

[77] Picard toolkit (Broad Institute, GitHub repository*,* 2019).

[78] Narasimhan V (2016). BCFtools/RoH: a hidden Markov model approach for detecting autozygosity from next-generation sequencing data. Bioinformatics.

[79] Chang CC (2015). Second-generation PLINK: rising to the challenge of larger and richer datasets. Gigascience.

[80] Lee T-H, Guo H, Wang X, Kim C, Paterson AH (2014). SNPhylo: a pipeline to construct a phylogenetic tree from huge SNP data. BMC Genomics.

[81] Stamatakis A (2014). RAxML version 8: a tool for phylogenetic analysis and post-analysis of large phylogenies. Bioinformatics.

[82] Korneliussen TS, Albrechtsen A, Nielsen R (2014). ANGSD: analysis of next generation sequencing data. BMC Bioinf..

[83] Salojärvi J (2017). Genome sequencing and population genomic analyses provide insights into the adaptive landscape of silver birch. Nat. Genet..

[84] R Core Team. R: A language and environment for statistical computing. (R Foundation for Statistical Computing, 2017).

[85] Liu X, Fu Y-X (2020). Stairway Plot 2: demographic history inference with folded SNP frequency spectra. Genome Biol..

[86] Li H, Durbin R (2011). Inference of human population history from individual whole-genome sequences. Nature.

[87] Excoffier L, Dupanloup I, Huerta-Sánchez E, Sousa VC, Foll M (2013). Robust demographic inference from genomic and SNP data. PLoS Genet..

[88] Zhang C, Dong S-S, Xu J-Y, He W-M, Yang T-L (2018). PopLDdecay: a fast and effective tool for linkage disequilibrium decay analysis based on variant call format files. Bioinformatics.

[89] Danecek P (2011). The variant call format and VCFtools. Bioinformatics.

[90] Weir BS, Cockerham CC (1984). Estimating F-statistics for the analysis of population structure. Evolution.

[91] Cingolani P (2012). A program for annotating and predicting the effects of single nucleotide polymorphisms, SnpEff. Fly (Austin).

[92] Chen J, Glémin S, Lascoux M (2017). Genetic diversity and the efficacy of purifying selection across plant and animal species. Mol. Biol. Evol..

[93] Salojärvi, J. jsalojar/PiNSiR: first release of PiNSiR 10.5281/zenodo.5136527 (2021).

[94] Bradbury PJ (2007). TASSEL: software for association mapping of complex traits in diverse samples. Bioinformatics.

[95] Rabah, S. et al. Plastome sequencing of ten nonmodel crop species uncovers a large insertion of mitochondrial DNA in cashew. *Plant Genome* **10**10.3835/plantgenome2017.03.0020 (2017). 10.3835/plantgenome2017.03.0020 29293812

[96] Chevreux B (2004). Using the miraEST assembler for reliable and automated mRNA transcript assembly and SNP detection in sequenced ESTs. Genome Res..

[97] Hahn C, Bachmann L, Chevreux B (2013). Reconstructing mitochondrial genomes directly from genomic next-generation sequencing reads—a baiting and iterative mapping approach. Nucleic Acids Res..

[98] Katoh K, Misawa K, Kuma K, Miyata T (2002). MAFFT: a novel method for rapid multiple sequence alignment based on fast Fourier transform. Nucleic Acids Res..

[99] Capella-Gutiérrez S, Silla-Martínez JM, Gabaldón T (2009). trimAl: a tool for automated alignment trimming in large-scale phylogenetic analyses. Bioinformatics.

[100] Chernomor O, von Haeseler A, Minh BQ (2016). Terrace aware data structure for phylogenomic inference from supermatrices. Syst. Biol..

