# Supplementary material for: Two divergent haplotypes from a highly heterozygous lychee genome suggest independent domestication events for early and late-maturing cultivars
Source: Nat Genet. 2022 Jan 3;54(1):73–83. doi: 10.1038/s41588-021-00971-3 (PMC8755541; doi:10.1038/s41588-021-00971-3)
Supplement: Supplementary file 1 — Supplementary Notes I–III, Note Figs. 1–3 and Figs. 1–13. [file 41588_2021_971_MOESM1_ESM.pdf]

nature portfolio

https://doi.org/10.1038/s41588-021-00971-3

Supplementary information

Two divergent haplotypes from a highly heterozygous lychee genome suggest independent domestication events for early and late-maturing cultivars

In the format provided by the authors and unedited

Two divergent haplotypes from a highly heterozygous lychee genome suggest independent domestication events for early and late-maturing cultivars

Supplementary Note I

Evolution of the lychee genome

All core eudicots descend from the gamma triplication event that occurred in a seven-chromosome ancestor approximately 120 Mya[1,2]. The grape genome retains 19 of the resulting 21 chromosomes that immediately followed the gamma event, with very few translocations and fusions accounting for the reduction in its haploid chromosome count [3]. The three Sapindaceae species lychee, longan, and yellowhorn each have 15 haploid chromosomes (n = 15), which requires at least six chromosomal fusion events to reduce the ancestral chromosome count of 21 to 15. We could clearly identify eight fusion events common to the three Sapindaceae genomes from the ancestral eudicot karyotype (AEK, **Extended Data Fig. 2**) [4]. Although lychee has the same number of chromosomes as yellowhorn, there were inter-chromosomal translocations or fusions that occurred along their respective genomes; e.g., chromosomes 3 and 12 of lychee were derived from translocations of chromosomes 1 and 15 of yellowhorn, and lychee chromosome 13 was derived from the fusion of yellowhorn chromosome 9 with its partial chromosome 13 (**Supplementary Fig. 3**).

Structure variants, gene copy number variants and chromosome rearrangements of lychee haplotypes

The HY and HH haplotypes have similar gene density profiles, while long terminal repeat retrotransposons (LTR) and nucleotide diversities show obvious distinctions (**Supplementary Fig. 8**). Other genomic differences, such as structural variants (SVs), gene copy number variants (CNVs) and chromosome rearrangements were also inspected between these two haplotypes. A large number of SVs was detected, accounting for ~11% of the haplotypic genomes and overlapping with as many as 3800 coding genes (**Supplementary table 22**). As for CNVs, about 8.45% genes displayed copy-number variation (**Supplementary table 23**). Besides that, ~700 genes were located in different genomic regions, likely due to chromosomal

rearrangements that occurred between two haplotypes (**Supplementary Fig. 9**).

Differential expression of alleles in lychee

Although almost half of the alleles showed differential expression, the quantity of DEAs in a single sample is limited with, on average, ~1700 DEAs per sample. The number of DEAs with expression bias toward either of the haplotypes is comparable (**Supplementary Table 31**), suggesting that allelic expression variation is time- or tissue-specific and that haplotype inclination of DEAs towards HY or HH is maintained at comparable levels. The only exception was the embryo transcriptome, within which HY-dominant DEAs (2939 alleles) were much more abundant than HH-dominant DEAs (1709, **Supplementary Table 35**). Gene ontology analysis revealed that organic acid binding activity and many enzymatic processes were enriched for the HY-dominant DEAs in lychee embryos, including transferase, esterase, monooxygenase and transaminase activities, suggesting that these HY-prone DEAs may contribute to the complex metabolic pathways leading to secondary metabolites (**Supplementary Table 36**).

SVP genes in lychee

Similar to the Arabidopsis *SVP* gene which is specifically expressed in leaf to repress the expression of the FT gene[5], these lychee *SVP* homologs were also mainly expressed in leaf or leaf buds, implying a conserved role in flowering regulation (**Extended Data Fig. 8c**). An *SVP* gene cluster (containing six genes) was also found in the genome of peach (*Prunus persica*), and deletion of all or part of four of the six *SVP* genes results in the *evergrowing* (*evg*) mutant, which does not show growth cessation and terminal bud formation in response to dormancy-inducing conditions such as low temperature or desiccation[6]. Given the fact that lychee and longan are subtropical/tropical fruit trees, while yellowhorn mainly grows in temperate regions, this different arrangement of *SVP* genes might contribute to the adaption of lychee and longan to subtropical and tropical climates.

Sapindaceae-expanded *VRN1* gene cluster

In terms of the tissue specificity of their expression, most of these *VRN1*-like genes were expressed at high levels in flower-buds and fruit tissues (pericarp, aril and embryo), implying roles in flower and fruit development. Considerable expression was also observed in leaf and floral primordia - characteristic plant parts that appear when buds start the process of floral induction, implying that these *VRN1-like* genes are probably involved in flowering induction as in Arabidopsis. The VRN1 cluster is located within a selective sweep region (**Extended Data Fig. 9c**), and three of these *VRN1-like* genes (*VRN1g*, *m*, *w*) display Ka/Ks values >1, suggesting possible positive selection in the cluster. The *VRN1g* and *VRN1v* genes were strongly expressed in flower and fruit pericarp, respectively, suggesting either subfunctionalization or neofunctionalization.

References:

1. Jiao, Y. *et al.* A genome triplication associated with early diversification of the core eudicots. *Genome Biol.* **13**, R3 (2012).
2. Chanderbali, A. S., Berger, B. A., Howarth, D. G., Soltis, D. E. & Soltis, P. S. Evolution of floral diversity: genomics, genes and gamma. *Philos. Trans. R. Soc. B Biol. Sci.* **372**, 20150509 (2017).
3. Jaillon, O. *et al.* The grapevine genome sequence suggests ancestral hexaploidization in major angiosperm phyla. *Nature* **449**, 463–467 (2007).
4. Murat, F., Armero, A., Pont, C., Klopp, C. & Salse, J. Reconstructing the genome of the most recent common ancestor of flowering plants. *Nat. Genet.* **49**, 490–496 (2017).
5. Lee, J. H. *et al.* Role of SVP in the control of flowering time by ambient temperature in Arabidopsis. *Genes Dev.* **21**, 397–402 (2007).
6. Bielenberg, D. G. *et al.* Sequencing and annotation of the evergrowing locus in peach [Prunus persica (L.) Batsch] reveals a cluster of six MADS-box transcription factors as candidate genes for regulation of terminal bud formation. *Tree Genet. Genomes* **4**, 495–507 (2008).

Supplementary Note II

The population demographies estimated with pairwise sequentially Markovian coalescent (PSMC), Stairway plot 2, SMC++ and Fastsimcoal2 all showed split times on the order of millions of years ago (**Supplementary Note Fig. 1**).

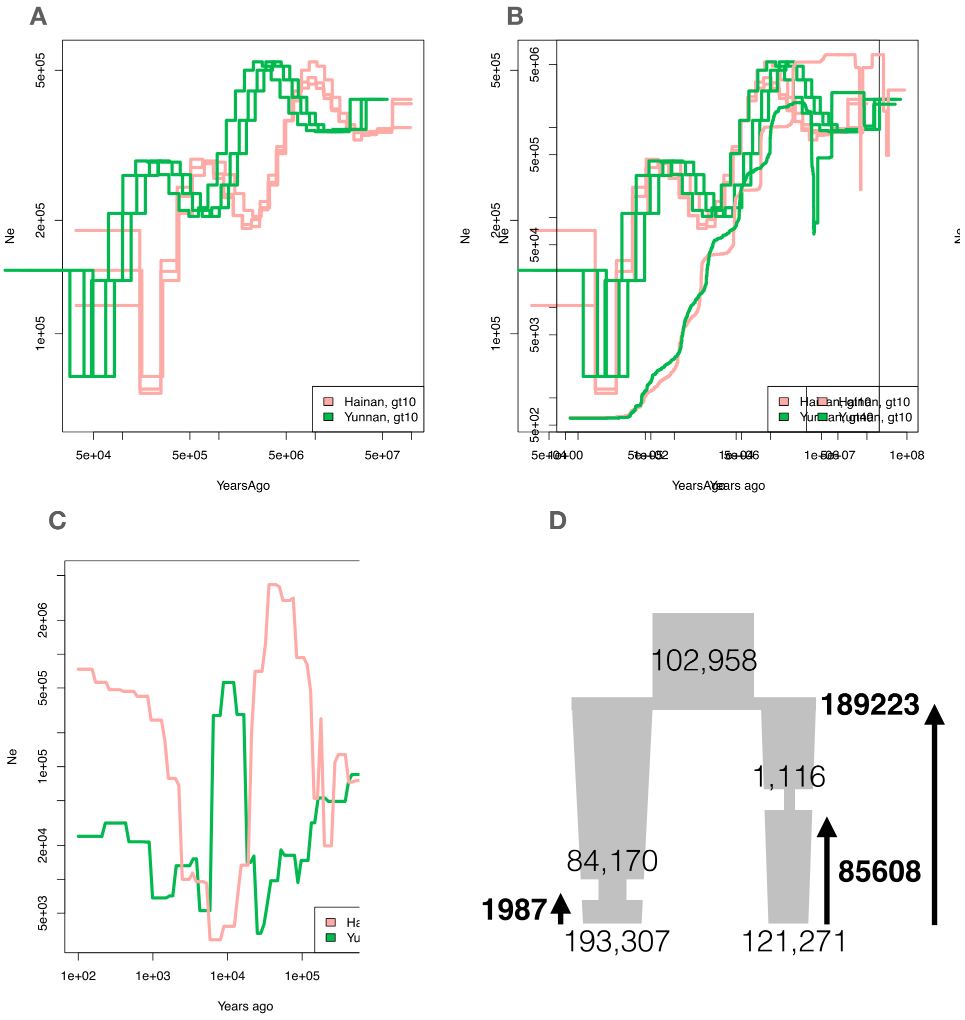

Supplementary Note Fig. 1. Population histories of lychee Yunnan (green) and Hainan (pink) wild populations estimated with pairwise sequentially Markovian Coalescent (A), Stairway plot2 (B), SMC++ (C) models and divergence times (in generations) estimated with Fastsimcoal2 (D).

This is a remarkably long period, as usually speciation events rather than within-species population splits occur on those time scales. Gene flow between populations might account for such patterns after a deep split time, but as the F_{ST} values (**Supplementary table 15**) suggest, there has been very little gene flow between the Hainan and Yunnan lychee populations. However, it is worth noticing that all demographic models show similar shapes of N_e trajectories, but with a consistent "shift" towards modern times in Yunnan compared to Hainan population

(**Supplementary Note Fig. 1**), implying more recent coalescence throughout the genome in the Yunnan population.

In coalescent modeling, highly heterozygous regions coalesce further back in time, and therefore this "shift" could have resulted from events that accelerate coalescence by reducing the genome-level heterozygosity, such as high levels of inbreeding, and selfing in particular, as was observed in the Yunnan population (**Supplementary Note Fig. 2**). To test for the effect of inbreeding on the applied coalescent models we simulated the loss of heterozygosity due to selfing in one Hainan individual by sampling allele distributions in heterozygous positions assuming Mendelian inheritance - that is, in the 1st generation, the probability of retaining heterozygosity in the selfed progeny was 0.5 (and in 2nd generation 0.5^2=0.25). If homozygous positions were simulated for the progeny, then either one of the parental alleles was selected with equal probability. Implemented in this manner, the model does not simulate selfing as it would occur in first generation progeny, where recombination would generate highly homozygous blocks while keeping the remaining heterozygous regions intact, but it rather simulates the loss of heterozygosity due to inbreeding throughout the population history, assuming a constant level of inbreeding at any given coalescent genome fragment. The net effect of the model then corresponds to 50% or 25% loss of heterozygosity due to selfing throughout the genome. One round of selfing reduced the nucleotide diversity in the Hainan individual from the initial π=0.0078 to 0.0039 in the first generation and to 0.00195 in the second generation, as expected.

In PSMC, the simulated selfing indeed resulted in a progressive shift of the demographic curves towards modern times (**Supplementary Note Fig. 2A**). We therefore conclude that inbreeding, and possibly selfing, is the most parsimonious explanation for the observed discrepancy of demographies between Yunnan and Hainan populations. Many of the current demographic models such as SMC++ take poorly into account the effect of inbreeding, and a case where sub-populations have different levels of inbreeding is particularly challenging. Our results point out that further model development is needed to take into account these different demographic scenarios.

In terms of coalescent theory, taking selfing into account is straightforward, since an old result proves coalescent selfing to be identical to coalescent random mating[1] with the coalescent times rescaled by a factor corresponding to the proportion of selfing, $N_e=1/(1+F)*N=(2-s)/2 * N$, where F denotes the inbreeding coefficient and *s* the proportion of selfed individuals. In other words, coalescence is indeed accelerated by selfing, since the rescaling factor is always <1. Hence, we adjusted generation times between the two populations to align the N_e trajectories with the following logic. Since nucleotide diversity π and mutation rate m are linearly dependent ($\pi = 4mN_e$), in the simulated trajectory, we adjusted the generation time to 2x the generation time used in the original PSMC modeling of the Hainan individual to take into account

one generation of selfing, and 4x the original generation time to adjust for two generations of selfing, respectively. This resulted in matching of the selfed trajectories with the original one, albeit with lower estimated effective population sizes (**Supplementary Note Fig. 2B**). For this simulated data, we also note that since the nucleotide diversity was reduced by 50%, and thus the observed heterozygosity was dropped to half, the resulting inbreeding coefficient is $F=1-H_{obs}/H_{exp}=0.5$, where H_{obs} are the observed and H_{exp} the expected heterozygosities, respectively. Therefore, to obtain scaling of N_e levels to the original non-selfed population, a re-scaling of the observed N_e values would be needed, that is, $N=(1+F)* N_e$. For 1st generation selfed individual this would mean adjustment by $N=1.5* N_e$ and for 2nd generation by $N=1.5^2* N_e$. Such rescaling indeed produces a relatively good fit **(Supplementary Note Fig. 2C)**, further demonstrating the applicability of the coalescent theory.

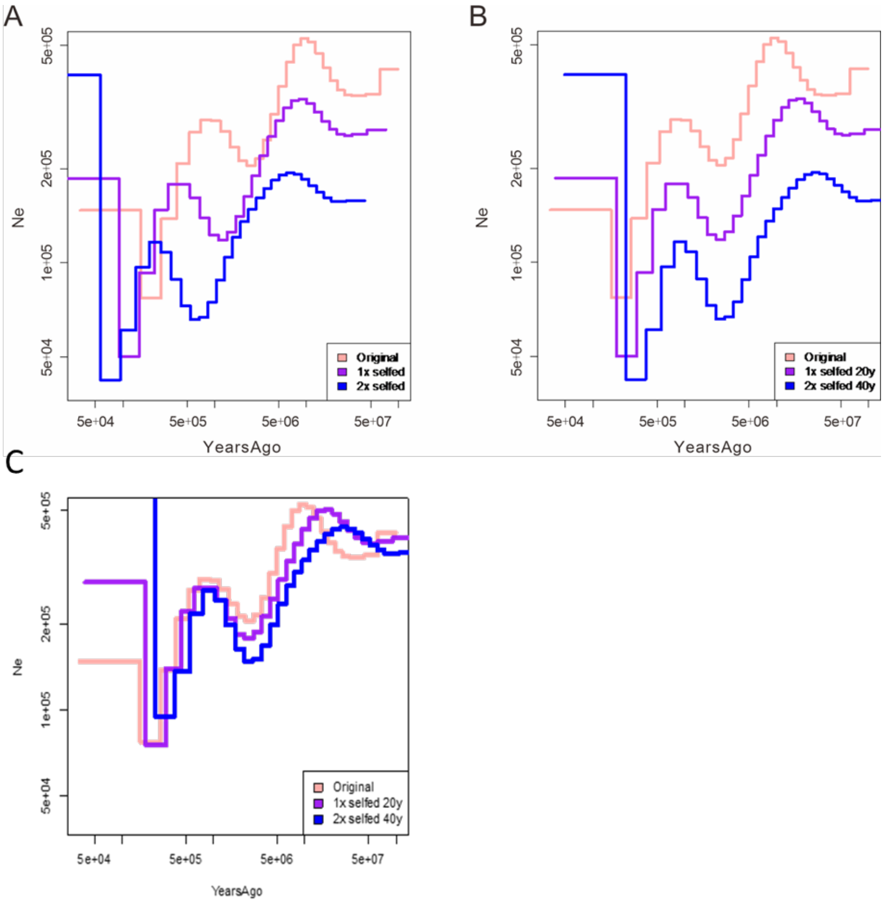

Supplementary Note Fig. *2.* ***A:*** *Population demographies estimated for a Hainan wild individual (HNW13; pink) and simulated selfed individuals after one generation (purple) and two generations (blue) of selfing.* ***B:*** *Assuming a linear relationship between selfing and generation time and adjusting accordingly, the different trajectories show concordant patterns.* ***C:*** *Further use of the relationship between inbreeding coefficient and observed heterozygosity will rescale the effective population sizes to the original levels before simulated inbreeding.*

For the actual lychee population data, the PSMC trajectories showed best match when the generation time of YNW was 3x the generation time input for HNW, reflecting 62.5% loss of heterozygosity due to inbreeding. In subsequent analyses this ratio was observed to be consistent across the different models (**Supplementary Note Fig. 3**). Note that the model does not claim that inbreeding would not occur in Hainan populations, only that inbreeding is more common in Yunnan.

Inbreeding may also have profound impacts on other analyses; for example, the short branch lengths seen in the SNP-based phylogenetic tree within the Yunnan population, and the distance between Yunnan and the outgroup (**Figure 2A, in main text**), could be due to the purging of SNPs upon selfing.

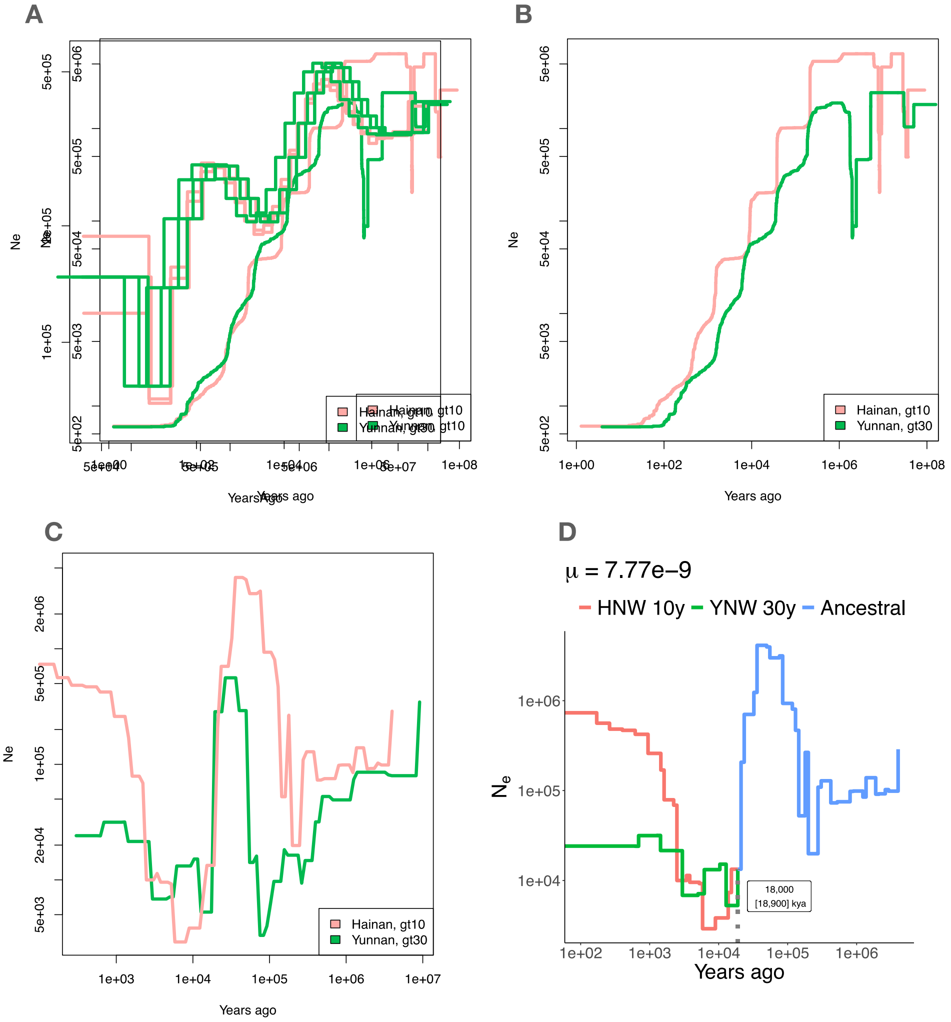

Supplementary Note Fig. 3.*Population histories of lychee Yunnan (green) and Hainan (pink) wild populations estimated with pairwise sequentially Markovian Coalescent (A), Stairway plot2 (B), SMC++ (C) models using a generation time of 3x Hainan generation time for the Yunnan population. After the adjustment, SMC++ modeling suggests a divergence time of 18,000 years ago, with considerably large confidence intervals (18-900kya). This is because generation time for lychee is largely unknown and could vary between 10-500 years; in a similar manner the*

mutation rate is highly variable, here for demonstration purposes, we fixed it to the mutation rate per generation estimated for peach[2].

References:

1. Nordborg, M. & Donnelly, P. The coalescent process with selfing. *Genetics* **146**, 1185–1195 (1997).
2. Wang, L. *et al.* Mutation rate analysis via parent–progeny sequencing of the perennial peach. II. No evidence for recombination-associated mutation. *Proc. R. Soc. B Biol. Sci.* **283**, 20161785 (2016).

Supplementary Note III

Differential expression analysis of allelic genes.

Identification of allelic gene pairs between HY and HH. Protein sequences of the two haplotypes were retrieved using TBtools[1]. Proteins from HY were used to perform BLASTP (2.7.1+)[2] against proteins from HH, and the best five hits were retained. Allelic gene pairs were selected according to the following rules: (1) the HY gene and one of the five hit genes from HH must be from a pair of homologous chromosomes; (2) they must have the same or close source from the reference genome (defined by GMAP[3] conversion). Syntenic gene pairs defined above were double-checked manually.

Identification of differential expression allelic genes (DEA). Thirty-nine RNA-seq libraries were collected including flowers, fruits, leaves, stems and roots (**Supplementary Table 20**). First, HY and HH haplotype sequences were merged and denoted as HY-HH. Then RNA-seq reads were trimmed using Trimmomatic[4] (v0.36) and mapped to HY-HH using the STAR[5] (2.5.3a) software using parameters '--alignIntronMax 20000 --alignMatesGapMax 25000 --outFilterMultimapNmax 1'. FPKM and counts were calculated based on uniquely mapping reads using StringTie[6] (v1.3.3b). Due to the absence of replication in most RNA-seq experiments, the identification of DEA adopted four standards.

(1) $FPKM_{HY} \geq 1$ or $FPKM_{HH} \geq 1$;

(2) $FPKM_{HY}/FPKM_{HH} \geq 2$ or $FPKM_{HY}/FPKM_{HH} \leq 0.5$;

(3) $Count_{HY} \geq 10$ or $Count_{HH} \geq 10$;

(4) *DEA was detected in at least 2 samples.*

Calculation of Ka and Ks between HY and HH. All genes syntenic between HY and HH were regarded as homologous gene pairs, and Ka and Ks were calculated in TBtools[1] with default parameters. The genes with Ka/Ks ≥ 1 were considered as potentially under positive selection pressure, while those with Ka/Ks ≤ 0.1 was considered to be under purifying selection pressure.

Identification of structural variants (SVs) and copy number variants (CNVs). The HY genome sequences were mapped against HH using the whole genome alignment program MUMmer 4[7]. Structural variants then were identified using the Web-based SV analytics tool Assemblytics[8]. Gene clusters defined in OrthoFinder2 (v2.3.3)[9] were compared between HY and HH, and those clusters with different gene quantities between the two haplotypes were summarized.

SNP analysis. Fifteen MVP blocks in the aforementioned haplotype phasing were split into HY and HH, which were then annotated using the ANNOVAR[10] (2019-10-24) software. SNPs located in coding sequences were selected, and transitions, transversions, synonymous and nonsynonymous changes were counted, respectively.

Identification of flowering related genes.

Lychee flowering related genes. A candidate flowering time gene list from FLOR-ID[11], MADS-box genes from PlantTFDB[12], and some additional genes were collected manually. In total, 481 Arabidopsis flowering related genes were gathered. BLASTP[2] (2.7.1+) was used to identify lychee flowering related genes with an E-value cutoff of 1e-5.

To identify tandem gene clusters, the flowering related genes identified above were compared with the gene families identified using OrthoFinder2[9] (v2.3.3). No more than three genes between any two consecutive genes with the same annotation were regarded as a cluster.

Phylogenetic tree of CONSTANS-like genes. The *CONSTANS*-like proteins of lychee and Arabidopsis were aligned using MUSCLE[13] (v3.8.31), and all gaps were removed using trimAl[14] (v1.4.rev22), and a maximum likelihood phylogenetic tree was obtained using FastTree[15] (v2.1.10) with default parameters.

PCR confirmed the 3.7kb deletion. DNA was extracted from leaf tissue. One forward, "a1", and two reverse primers, "b1", "c1", were designed with the following sequences - a1: ATCTCAAACGAGTCCTTACTTAGGTT, b1: ATTGCATATTTACTACCACCAACATC, and c1: TAGTTGCAACACTTTTCAGTGATGCT. PCR was performed in 30-μL volumes containing 0.7 μL of gDNA from different varieties, 1.5 μL of specific primers, 11.3 μL of distilled, deionized water, and 15 μL of 2 × Taq Master Mix (Dye Plus) (Vazyme) using a T100™ Thermal Cycler (Bio-Rad) PCR amplification system. The reaction conditions for thermal cycling were 95°C for 3 min followed by 30 cycles of 95°C for 15 s, 55°C for 15 s, and 72°C for 40 s. Thereafter, PCR products were visualized by 1% agarose gel electrophoresis with a 2kb DNA ladder.

Syntenic relationships of the *VRN1* gene cluster. Genome sequences of 18 core eudicot species were obtained from public databases (**Supplementary Table 36**). Synteny blocks between each pair of species were identified using MCScanX[16] with protein sequences. Genome sequences within this cluster region were searched against Pfam[17] to predict the number of B3 domains. Synteny blocks between the remaining 16 species and *V. vinifera*[18] were also identified, the same method was used to count the number of B3 domains was used. Conserved syntenic relationships for the *VRN1* gene cluster were viewed using JCVI[19] (v0.8.4) python packages. The gene models in this syntenic cluster region of *L. chinensis, D. longan*[20]*, X. sorbifolium*[21] and *C. sinensis*[22] were corrected manually using RNA-seq data.

The homologous genes of *AtVRN1* among 18 core eudicot species were also retrieved using BLASTP[2], then aligned using MUSCLE[13] (v3.8.31). All gaps were removed using trimAl[14] (v1.4.rev22), and a maximum likelihood phylogenetic tree was constructed with 1000 replicates using IQ-TREE[23] (v1.6.10) with '-bnni' parameters.

Sweep region analysis. Prior to analysis the data was filtered in Plink2 with --mind 0.5 --maf 0.05 --geno 0.1 --hwe 1e-5 --var-min-qual 30 options, outgroup species were removed, and only biallelic sites were selected. Allele frequencies were calculated in Plink2 and used for forming the global allele frequency spectrum in

Sweepfinder2. Subsequently SweepFinder2[24] was used to identify sweep regions in the lychee genome with 200 kb windows.

To obtain a critical value from neutral evolution we followed the procedure in Huber et al[25]. Briefly, we used Fastsimcoal to fit a model to two-dimensional site-frequency spectrum where late-maturing cultivars as well as Bobai county individuals were assigned to greater Hainan population, whereas Vietnam, Daxin, and extremely early maturing cultivars were assigned to Yunnan population. The best model from 100 random initializations was selected as the model describing neutral evolution. The demography was then implemented in MSMS simulator[26] using mutation rate from peach[27]; recombination rate was calculated assuming 2 cross-overs per chromosome (5.8e-8 crossovers / bp), and chromosome length of 34Mb; this is slightly higher than experimental crossover rates in Arabidopsis[28] or peach[27]. Since Sweepfinder2 statistic is somewhat sensitive to recombination rate this represents a higher bound for sweep statistics from neutral evolution. To obtain the critical value, we simulated 1000 1Mb blocks and estimated allele frequencies from this set. The Sweepfinder 2 was then run to calculate the statistic in 200kb blocks similar to the genome analyses. The first and last statistic from each run were dropped out since the window overlapped with simulated chromosome ends. The critical value was then obtained as the 95% quantile from the simulated statistics. Sweepfinder2 statistics larger than the critical value were then considered as true positives.

References:

1. Chen, C. *et al.* TBtools: An Integrative Toolkit Developed for Interactive Analyses of Big Biological Data. *Mol. Plant* **13**, 1194–1202 (2020).
2. Camacho, C. *et al.* BLAST+: architecture and applications. *BMC Bioinformatics* **10**, 421 (2009).
3. Wu, T. D. & Watanabe, C. K. GMAP: a genomic mapping and alignment program for mRNA and EST sequences. *Bioinformatics* **21**, 1859–1875 (2005).
4. Bolger, A. M., Lohse, M. & Usadel, B. Trimmomatic: a flexible trimmer for Illumina sequence data. *Bioinformatics* **30**, 2114–2120 (2014).
5. Dobin, A. *et al.* STAR: ultrafast universal RNA-seq aligner. *Bioinformatics* **29**, 15–21 (2013).
6. Pertea, M., Kim, D., Pertea, G. M., Leek, J. T. & Salzberg, S. L. Transcript-level expression analysis of RNA-seq experiments with HISAT, StringTie and Ballgown. *Nat. Protoc.* **11**, 1650–1667 (2016).
7. Marçais, G. *et al.* MUMmer4: A fast and versatile genome alignment system. *PLOS Comput. Biol.* **14**, e1005944 (2018).
8. Nattestad, M. & Schatz, M. C. Assemblytics: a web analytics tool for the detection of variants from an assembly. *Bioinformatics* **32**, 3021–3023 (2016).
9. D.M., E. & S., K. OrthoFinder2: fast and accurate phylogenomic orthology analysis from gene sequences. *bioRxiv* 466201 (2018) doi:10.1101/466201.
10. Wang, K., Li, M. & Hakonarson, H. ANNOVAR: functional annotation of genetic variants from high-throughput sequencing data. *Nucleic Acids Res.* **38**, e164–e164 (2010).

11. Bouché, F., Lobet, G., Tocquin, P. & Périlleux, C. FLOR-ID: an interactive database of flowering-time gene networks in Arabidopsis thaliana. *Nucleic Acids Res.* **44**, D1167–D1171 (2015).
12. Jin, J. *et al.* PlantTFDB 4.0: toward a central hub for transcription factors and regulatory interactions in plants. *Nucleic Acids Res.* **45**, D1040–D1045 (2016).
13. Edgar, R. C. MUSCLE: a multiple sequence alignment method with reduced time and space complexity. *BMC Bioinformatics* **5**, 113 (2004).
14. Capella-Gutiérrez, S., Silla-Martínez, J. M. & Gabaldón, T. trimAl: a tool for automated alignment trimming in large-scale phylogenetic analyses. *Bioinformatics* **25**, 1972–1973 (2009).
15. Price, M. N., Dehal, P. S. & Arkin, A. P. FastTree 2 – Approximately Maximum-Likelihood Trees for Large Alignments. *PLoS One* **5**, e9490 (2010).
16. Wang, Y. *et al.* MCScanX: a toolkit for detection and evolutionary analysis of gene synteny and collinearity. *Nucleic Acids Res.* **40**, e49–e49 (2012).
17. Finn, R. D. *et al.* Pfam: the protein families database. *Nucleic Acids Res.* **42**, D222–D230 (2014).
18. Jaillon, O. *et al.* The grapevine genome sequence suggests ancestral hexaploidization in major angiosperm phyla. *Nature* **449**, 463–467 (2007).
19. Tang, H., Krishnakumar, V. & Li, J. jcvi: JCVI utility libraries. (2015) doi:10.5281/zenodo.31631.
20. Lin, Y. *et al.* Genome-wide sequencing of longan (Dimocarpus longan Lour.) provides insights into molecular basis of its polyphenol-rich characteristics. *Gigascience* **6**, (2017).
21. Bi, Q. *et al.* Pseudomolecule-level assembly of the Chinese oil tree yellowhorn (Xanthoceras sorbifolium) genome. *Gigascience* **8**, (2019).
22. Xu, Q. *et al.* The draft genome of sweet orange (Citrus sinensis). *Nat. Genet.* **45**, 59–66 (2013).
23. Chernomor, O., von Haeseler, A. & Minh, B. Q. Terrace Aware Data Structure for Phylogenomic Inference from Supermatrices. *Syst. Biol.* **65**, 997–1008 (2016).
24. Degiorgio, M., Huber, C., Hubisz, M., Hellmann, I. & Nielsen, R. SWEEPFINDER2: Increased sensitivity, robustness, and flexibility. *Bioinformatics* **32**, (2015).
25. Huber, C. D., DeGiorgio, M., Hellmann, I. & Nielsen, R. Detecting recent selective sweeps while controlling for mutation rate and background selection. *Mol. Ecol.* **25**, 142–156 (2016).
26. Ewing, G. & Hermisson, J. MSMS: A coalescent simulation program including recombination, demographic structure and selection at a single locus. *Bioinformatics* **26**, 2064–2065 (2010).
27. Wang, L. *et al.* Mutation rate analysis via parent–progeny sequencing of the perennial peach. II. No evidence for recombination-associated mutation. *Proc. R. Soc. B Biol. Sci.* **283**, 20161785 (2016).
28. Rowan, B. A. *et al.* An Ultra High-Density Arabidopsis thaliana Crossover. *Genetics* **213**, 771–787 (2019).

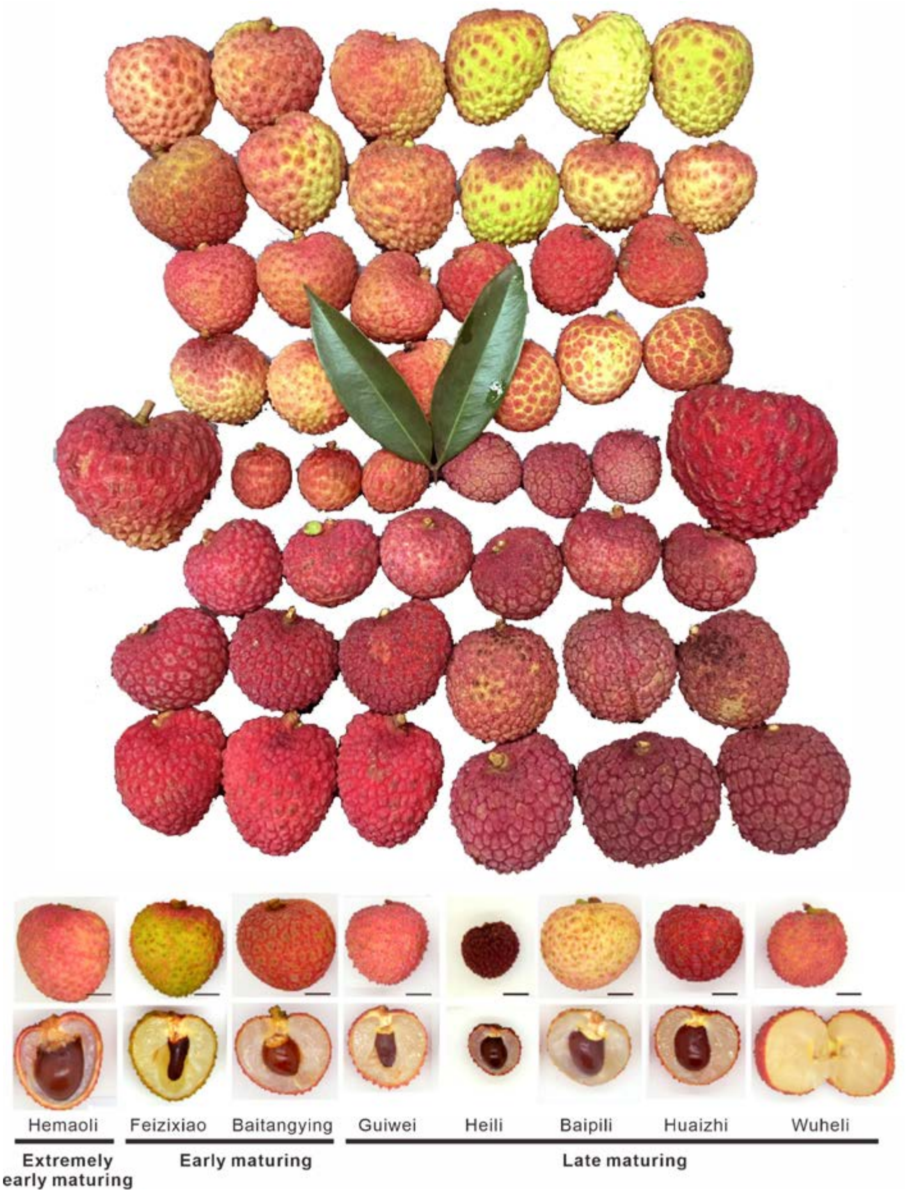

Supplementary Fig. 1. Representative fruits from diverse lychee accessions (scale bar, 1 cm).

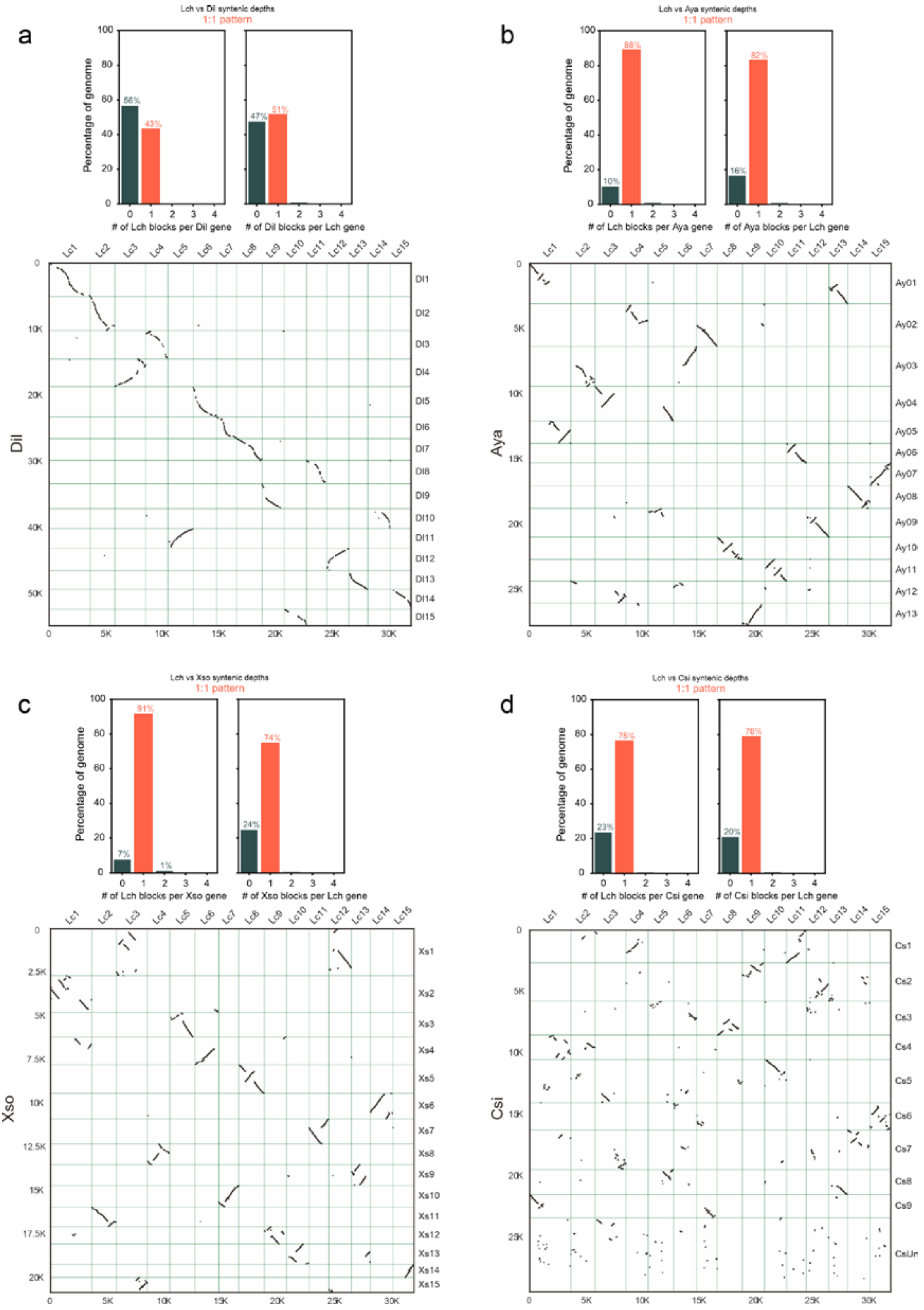

Supplementary Fig. 2. Syntenic gene relationships between four species and lychee. Most of the genes are of 1 to 1 syntenic relationship among the four Sapindaceae species. Dil: D. *Longan*, *longan*, Aya: *A. yangbiense*, maple, Xso: *X. sorbifolium*, yellowhorn, Csi: *C. sinensis*, orange and Lch: *L. chinensis*, lychee.

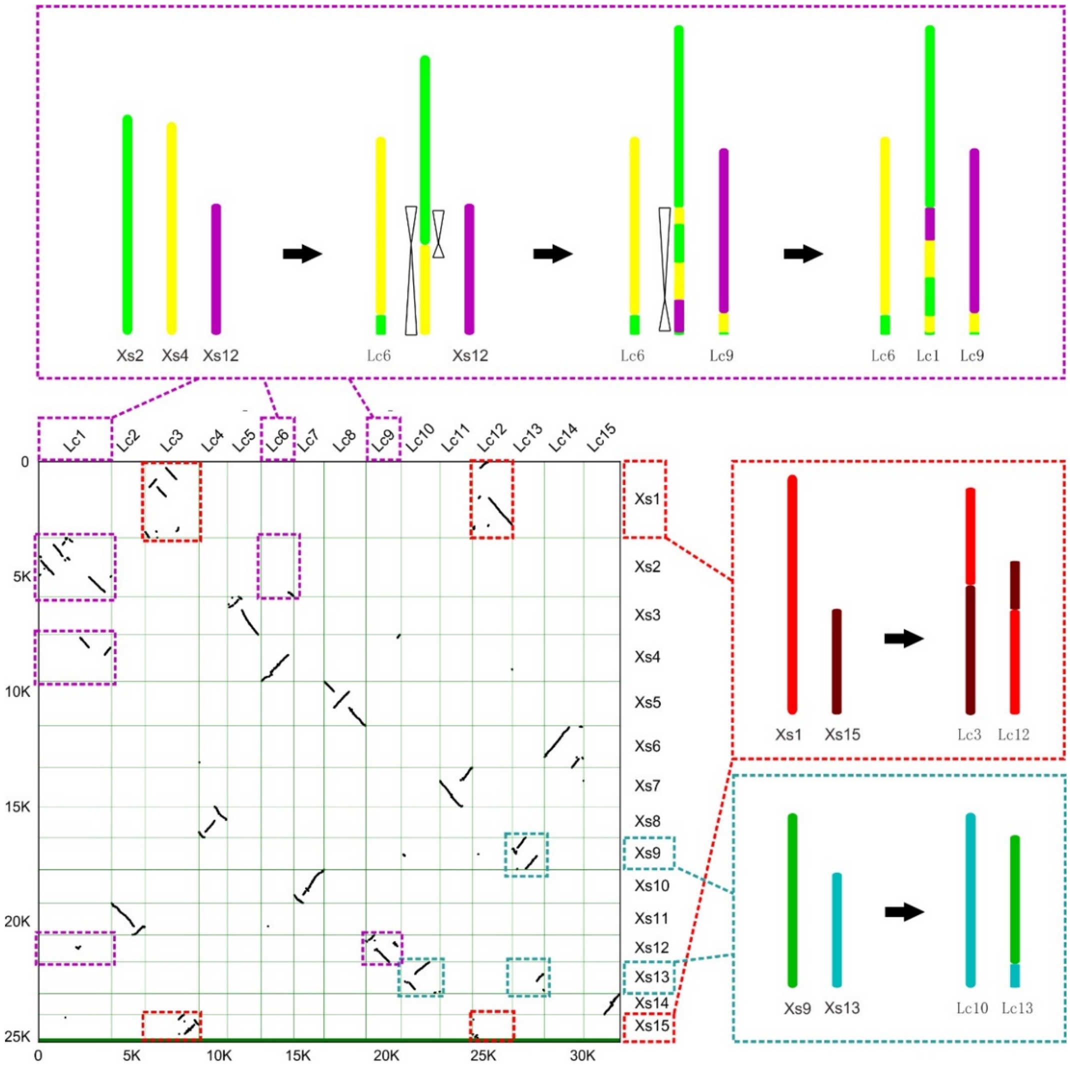

Supplementary Fig. 3: Deduced karyotype changes between yellowhorn and lychee. Several translocations and inversions were detected between yellowhorn and lychee.

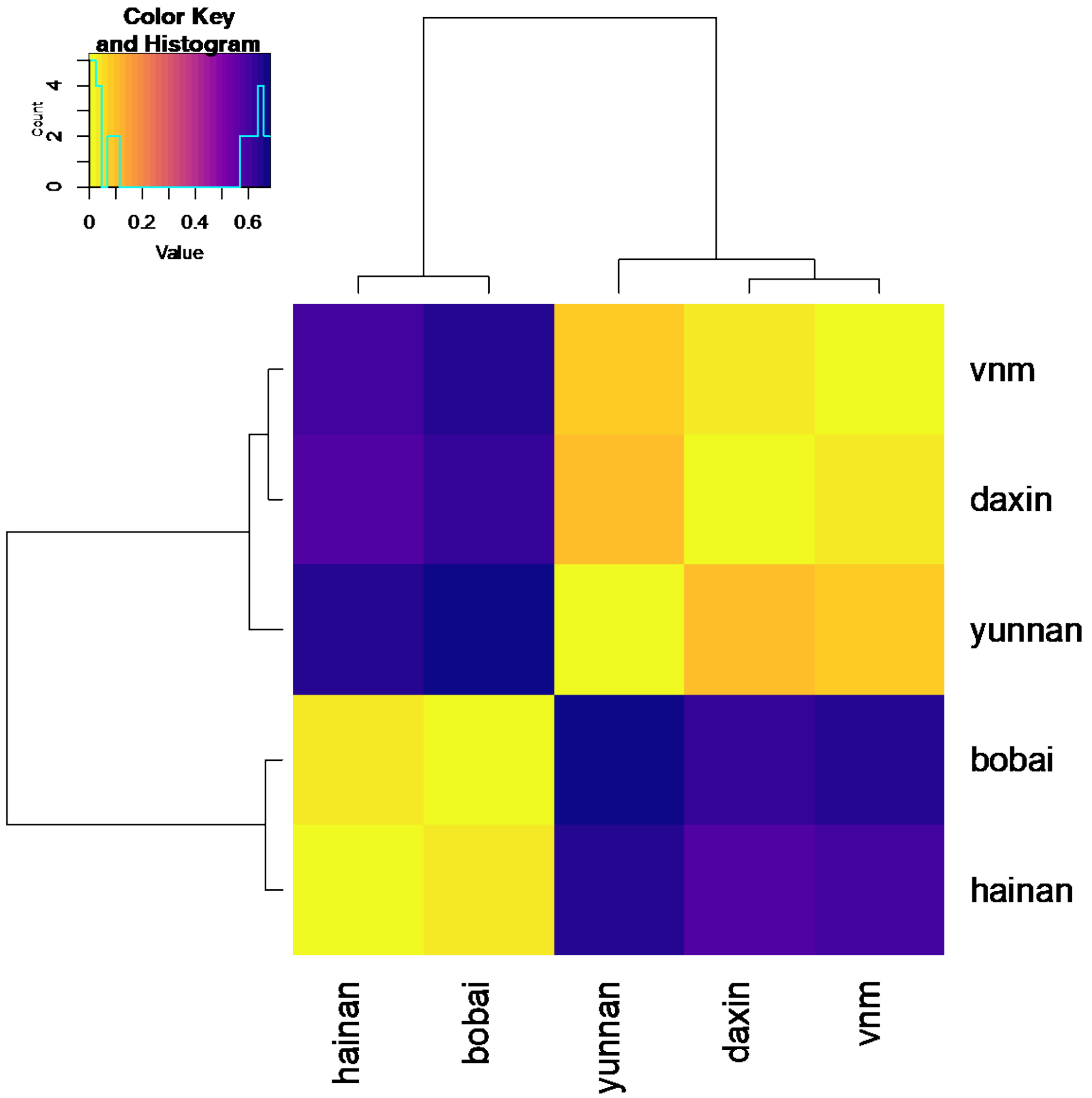

Supplementary Fig. 4: A heatmap of fixation index Fst values between wild lychee populations. Low values indicate high level of genetic overlap and gene flow, whereas high values point to divergent populations. Clustering was carried out using Euclidean distances and complete linkage hierarchical clustering.

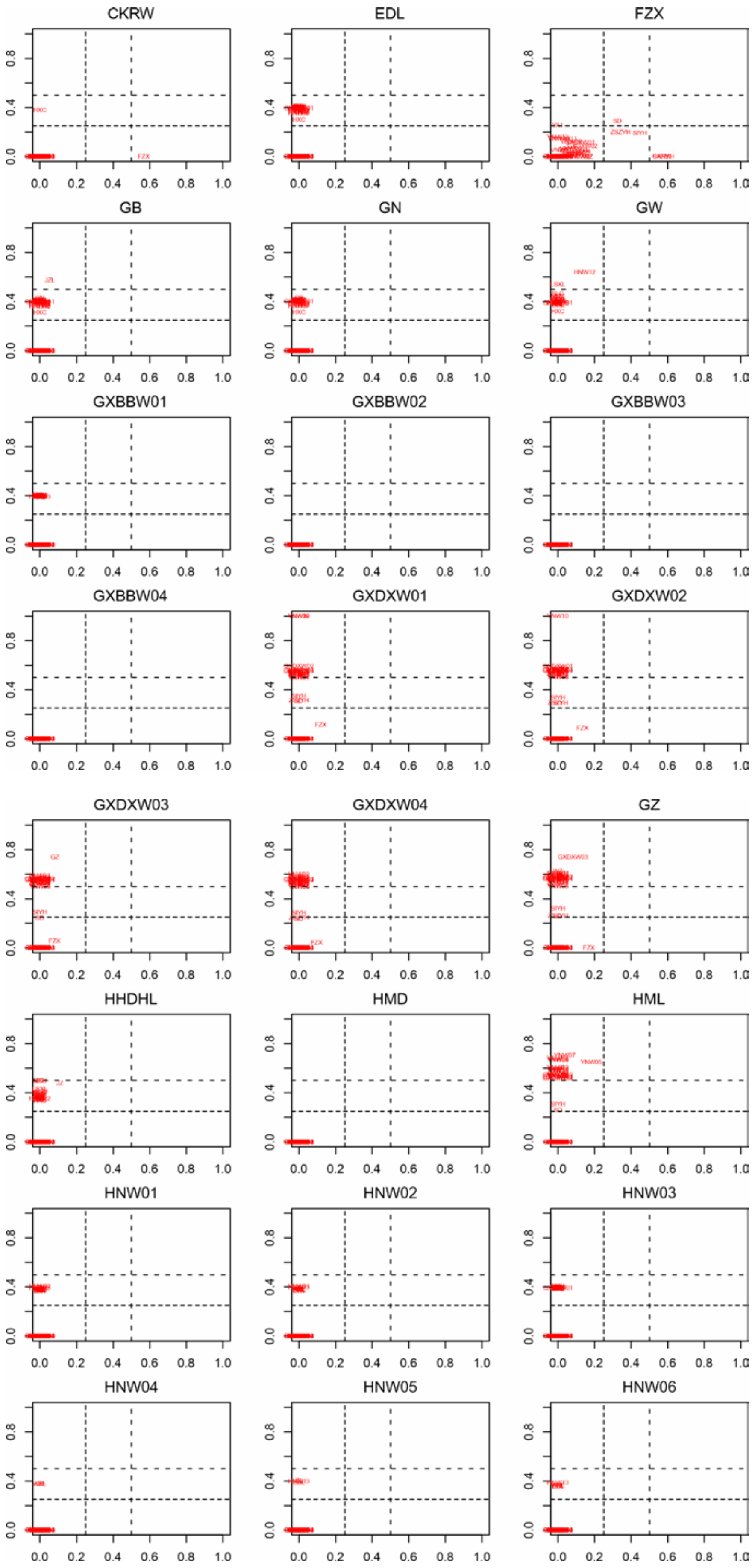
CKRW
EDL
FZX
GB
GN
GW
GXBBW01
GXBBW02
GXBBW03
GXBBW04
GXDXW01
GXDXW02
GXDXW03
GXDXW04
GZ
HHDHL
HMD
HML
HNW01
HNW02
HNW03
HNW04
HNW05
HNW06

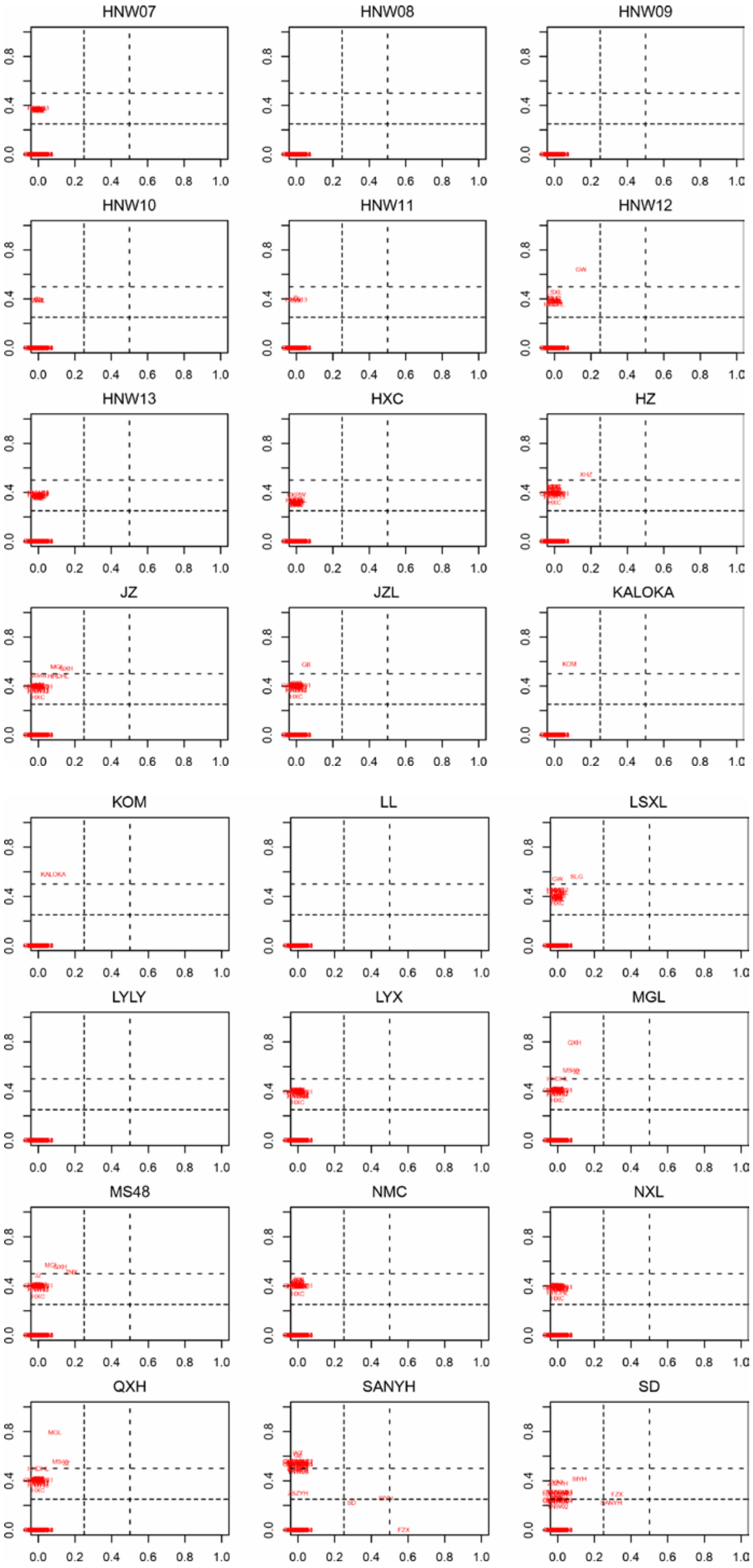
HNW07
HNW08
HNW09
HNW10
HNW11
HNW12
HNW13
HXC
HZ
JZ
JZL
KALOKA
KOM
LL
LSXL
LYLY
LYX
MGL
MS48
NMC
NXL
QXH
SANYH
SD

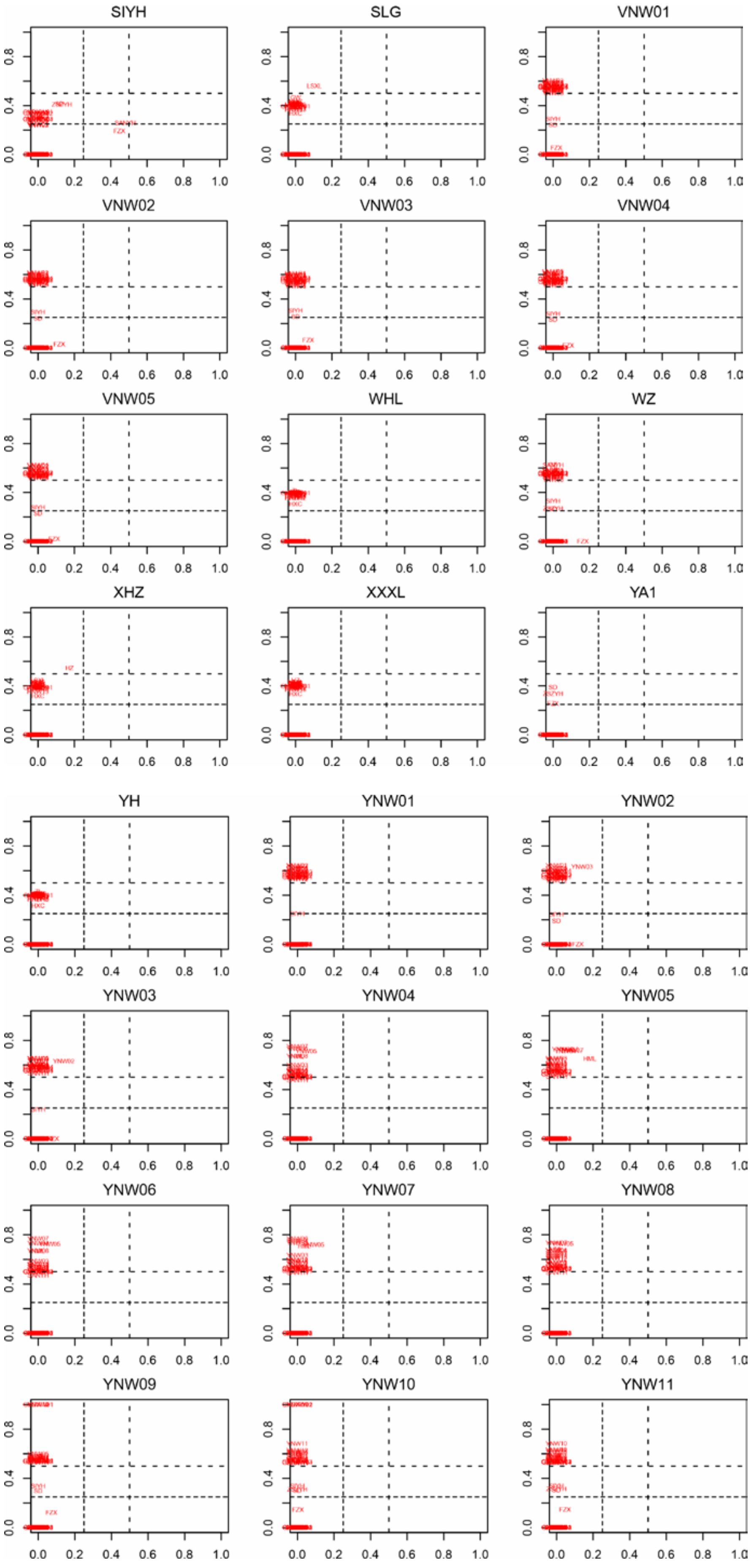
SIYH
SLG
VNW01
VNW02
VNW03
VNW04
VNW05
WHL
WZ
XHZ
XXXL
YA1
YH
YNW01
YNW02
YNW03
YNW04
YNW05
YNW06
YNW07
YNW08
YNW09
YNW10
YNW11

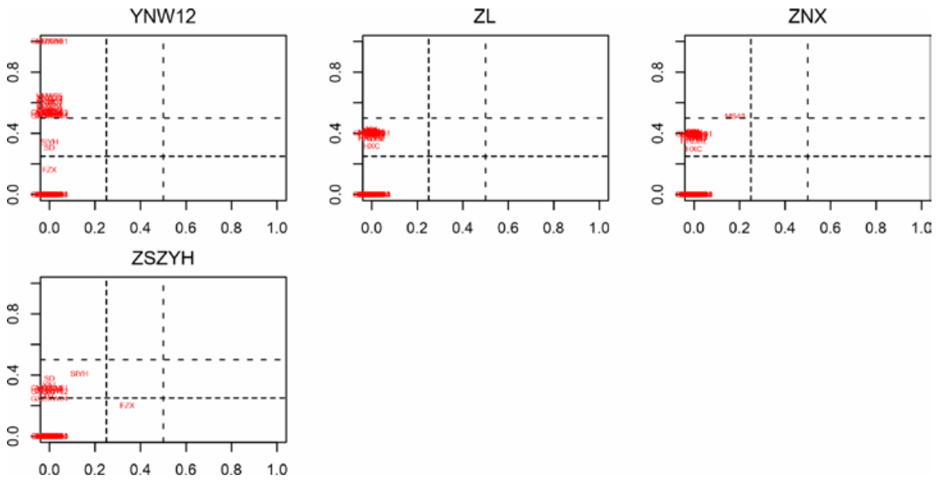

Supplementary Fig. 5 Analysis of IBS (Identity-by-state) relatedness between lychee accessions revealed close relationships among Yunnan, Daxin and Vietnam wild populations. X-axis: homozygote Z2 relationship, Y-axis: heterozygote Z1 relationship.

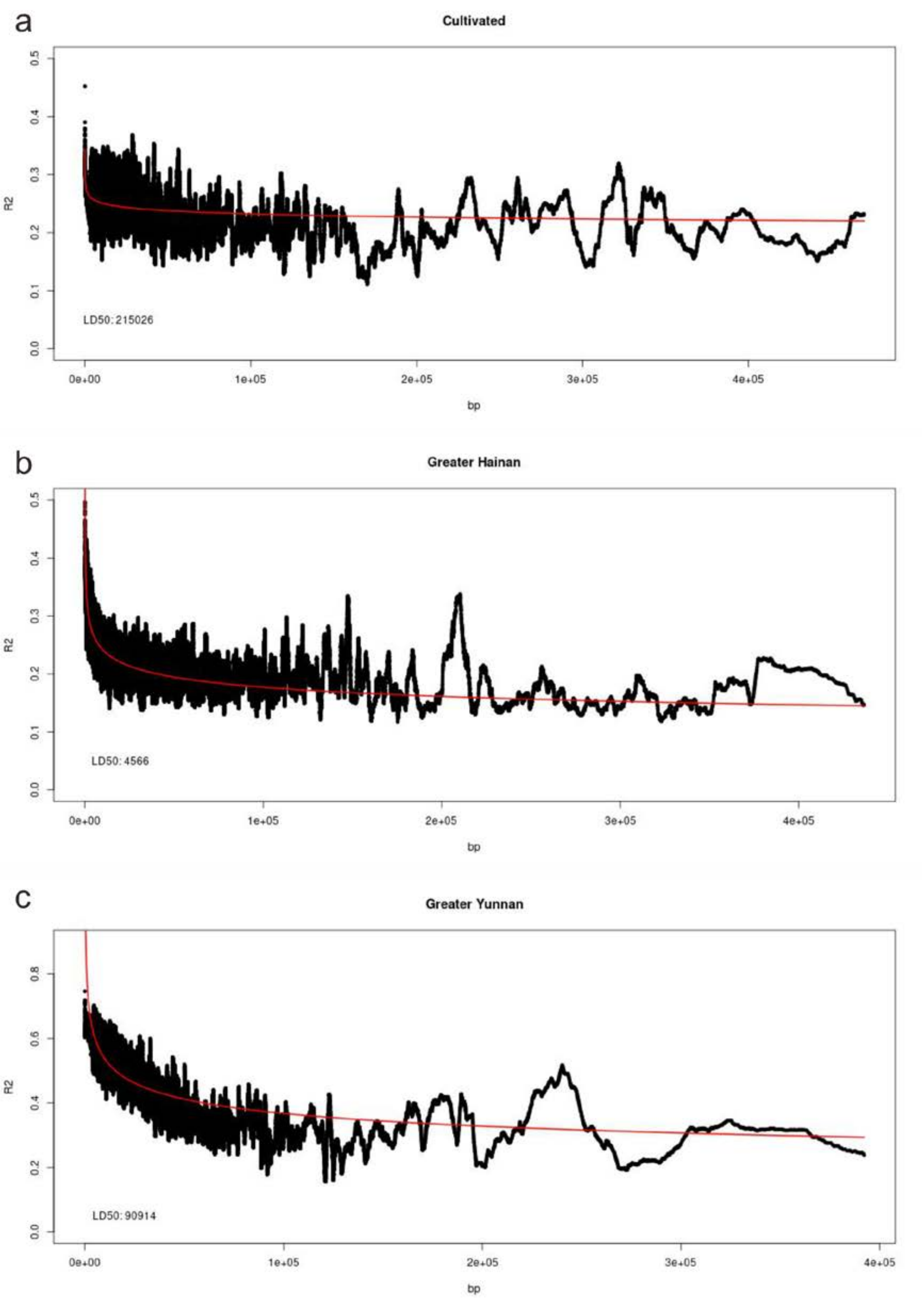

Supplementary Fig. 6: Genome-wide average LD decay estimated for cultivated (a) and wild lychee accessions (Hainan: b; Yunnan: c).

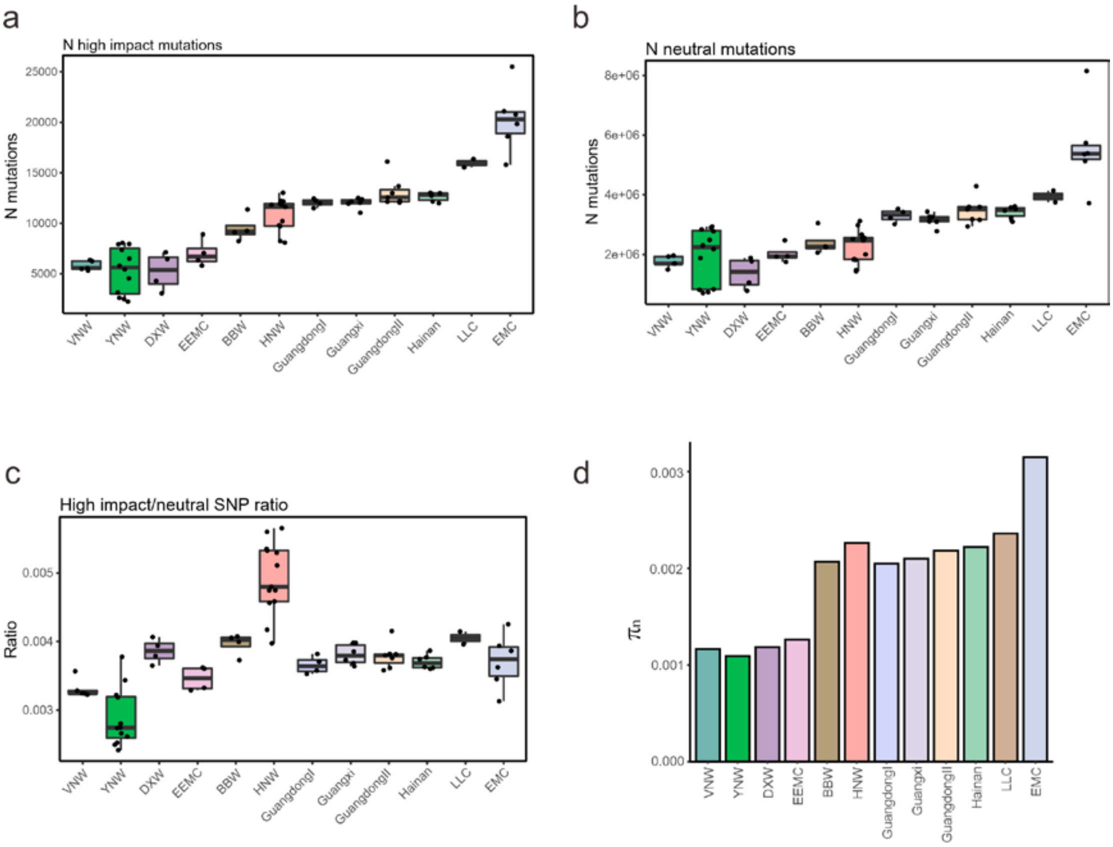

Supplementary Fig. 7: High impact and neutral mutations among cultivars with different origins show that breeding may have purged deleterious alleles. In **a**, **b** and **c**, box plots and points show the numbers of high impact, neutral SNPs that classified with snpEff and its' ratio, respectively. Box plots show the median, box edges represent the 25th and 75th percentiles, whiskers represent the maximum and minimum data points within 1.5× interquartile range outside box edges. The numbers of accessions (left to right) are n=5, 12, 4, 4, 4, 13, 4, 6, 7, 6, 2, 6, respectively.

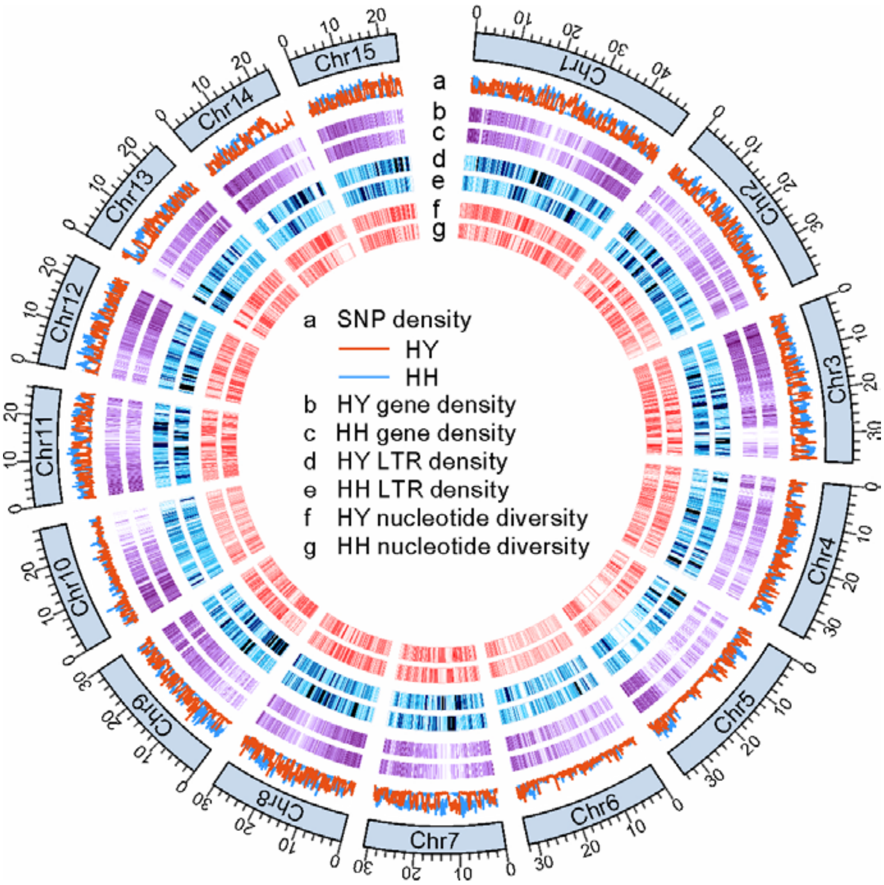

Supplementary Fig. 8: Genome features in HY and HH haplotypes.

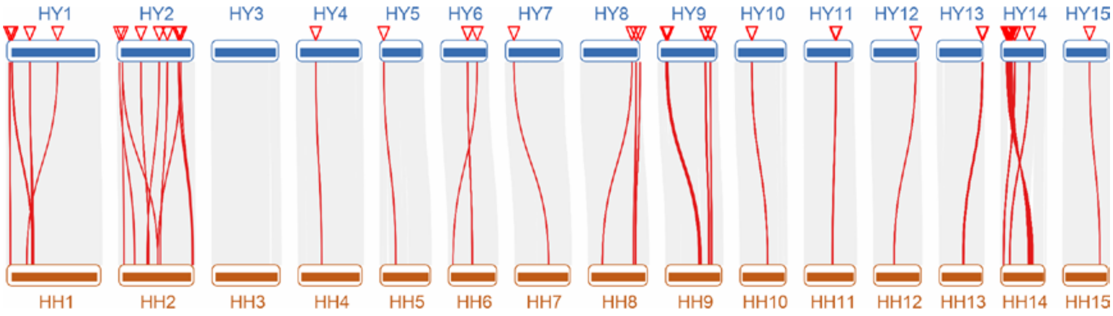

Supplementary Fig. 9: Chromosomal rearrangement events detected between HY and HH.

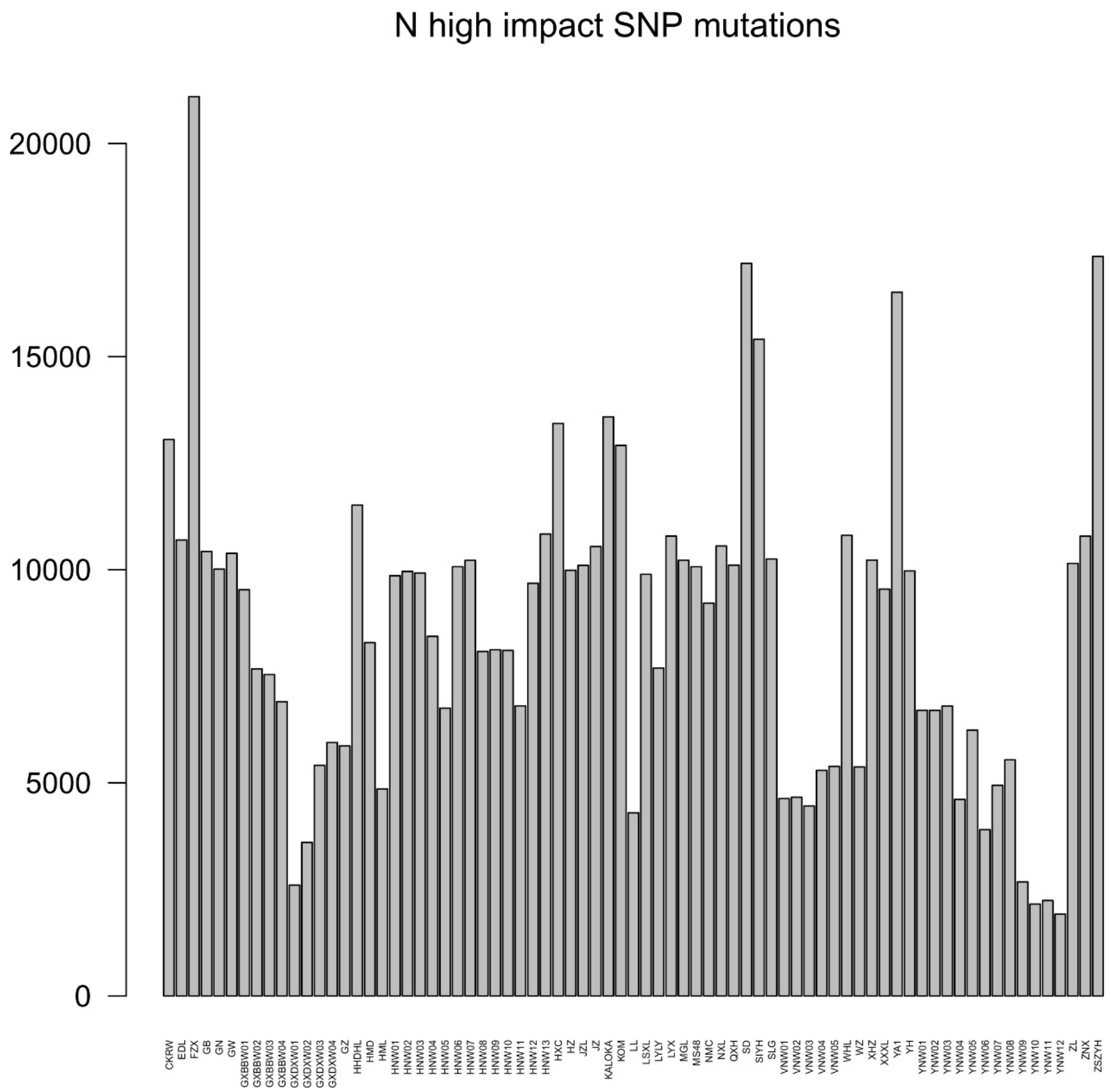

Supplementary Fig. 10: Admixed cultivars displayed high numbers of disruptive SNPs.

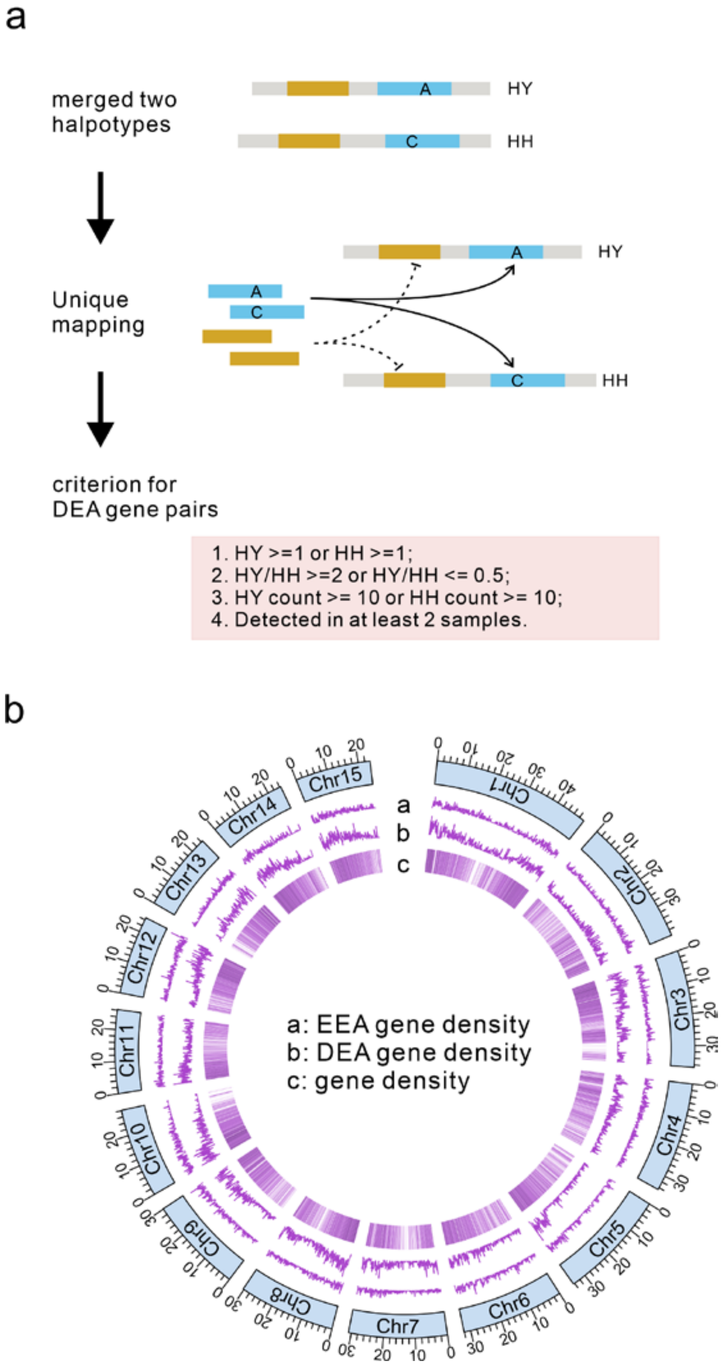

Supplementary Fig. 11: Differentially expressed allelic genes in lychee. a. DEAs were defined according to the following criteria: (1) FPKM expression is larger than 1 in HH or HY; (2) Expression fold change of HY/HH or HH/HY are larger than 2; (3) Raw read counts for a gene are more than 10 in HY or HH; and (4) The gene must be identified as a DEA in at least two RNA-seq libraries. b. Distribution of DEAs and equally expressed alleles (EEAs) in the lychee genome.

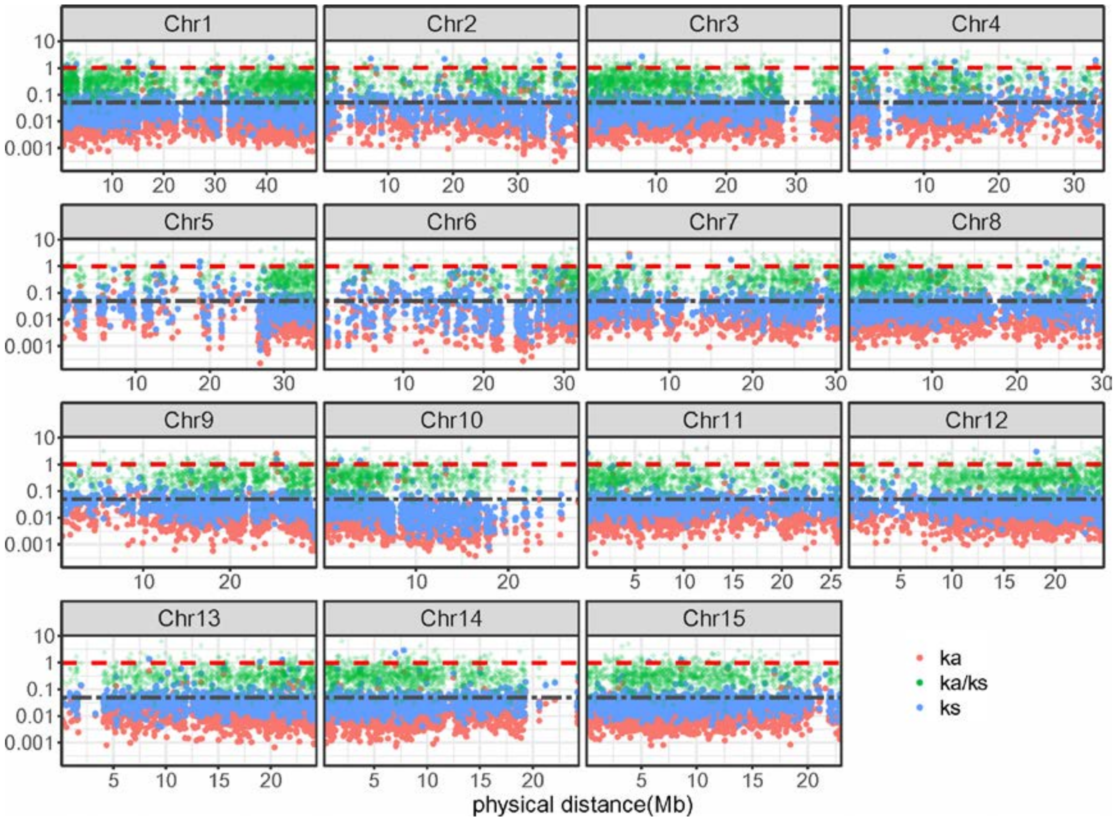

Supplementary Fig. 12: Ka and Ks between the two haplotypes. Red points: Ka, blue points: Ks, green points: Ka/Ks; red dashed line and grey dot-dashed line represent 1.0 and 0.05, respectively.

```
                                                               1st B3 domain
COL307_HY      MMKEESSDGG GGATNWARVC DTCRSAACTV YCKADLAYLC AVCDSRVHAA NRVASRHERV
COL307_HH      MMKEESSDGG GGANNWARVC DTCRSAACTV YCKADLAYLC AVCDARVHAA NRVASRHERV
               ********** ***.****** ********** ********** ****:***** **********

                            2nd B3 domain
COL307_HY      FVCEACEQAP AAFLCKADAA SLCAACDADI HSANPLARRH HRVPILPISG SIYGQTEDGF
COL307_HH      FVCEACEQAP AAFLCKADAA SLCATCDADI HSANPLARRH HRVPILPISG SIYGQTEDGF
               ********** ********** ****:***** ********** ********** **********

COL307_HY      VGAEDEDDDE EAASWLLMNP TKNTNNTDNG FLLPGEADEY LDLVDYNGEN QYVDHYNHHQ
COL307_HH      VGAEDEDDDE EAASWLLMKP TKNTNNTDNG FLFPGEVDEY LDLVDYNGEN QYVDHYKHHQ
               ********** ********:* ********** **:***.*** ********** ******:***

COL307_HY      QRQQHFGKNY ISDSIVPVQC QVKDQQNQEN FPLGLDYESP KAAYSYNGSL SHSLSVSSTD
COL307_HH      QRQQHFGKNY ISDSIVPVQY KVKDQQNQEN FPLGLDYESS KAAYSYNGSL SHCLSVSSTD
               ********** *********  :********* *********. ********** **.*******
                                                                    CCT domain
COL307_HY      VGVVPESTS- DMTISHSRPP KGTIDLFSGP PIQMPPQLTP MDREARVLRY REKKKTRKFE
COL307_HH      VGVVPESTTS DISISHSRPP KGTIDLFSGP PIQTPPQLTP MDREARVLRY REKKKTRKFE
               ********:  *::******* ********** *** ****** ********** **********

COL307_HY      KTIRYASRKA YAETRPRIKG RFAKRTDVEV EIDQMFSTTL ITDAGYGIVP SF
COL307_HH      KTIRYASRKA YAETRPRIKG RFAKRTDVEV EIDQMFSTTL ITDAGYGIVP SF
               ********** ********** ********** ********** ********** **
```

Supplementary Fig. 13: Protein sequence comparison of COL307 between the two lychee haplotypes.
