# Supplementary material for: Two divergent haplotypes from a highly heterozygous lychee genome suggest independent domestication events for early and late-maturing cultivars
Source: Nat Genet. 2022 Jan 3;54(1):73–83. doi: 10.1038/s41588-021-00971-3 (PMC8755541; doi:10.1038/s41588-021-00971-3)
Supplement: Supplementary file 2 — Reporting Summary [file 41588_2021_971_MOESM2_ESM.pdf]

nature research

Corresponding author(s): Rui Xia

Last updated by author(s): Jul 25, 2021

Reporting Summary

Nature Research wishes to improve the reproducibility of the work that we publish. This form provides structure for consistency and transparency in reporting. For further information on Nature Research policies, see our Editorial Policies and the Editorial Policy Checklist.

Statistics

For all statistical analyses, confirm that the following items are present in the figure legend, table legend, main text, or Methods section.

n/a	Confirmed	
☐	☒	The exact sample size (*n*) for each experimental group/condition, given as a discrete number and unit of measurement
☐	☒	A statement on whether measurements were taken from distinct samples or whether the same sample was measured repeatedly
☐	☒	The statistical test(s) used AND whether they are one- or two-sided<br>*Only common tests should be described solely by name; describe more complex techniques in the Methods section.*
☒	☐	A description of all covariates tested
☒	☐	A description of any assumptions or corrections, such as tests of normality and adjustment for multiple comparisons
☐	☒	A full description of the statistical parameters including central tendency (e.g. means) or other basic estimates (e.g. regression coefficient) AND variation (e.g. standard deviation) or associated estimates of uncertainty (e.g. confidence intervals)
☐	☒	For null hypothesis testing, the test statistic (e.g. *F*, *t*, *r*) with confidence intervals, effect sizes, degrees of freedom and *P* value noted<br>*Give P values as exact values whenever suitable.*
☒	☐	For Bayesian analysis, information on the choice of priors and Markov chain Monte Carlo settings
☒	☐	For hierarchical and complex designs, identification of the appropriate level for tests and full reporting of outcomes
☒	☐	Estimates of effect sizes (e.g. Cohen's *d*, Pearson's *r*), indicating how they were calculated

Our web collection on statistics for biologists contains articles on many of the points above.

Software and code

Policy information about availability of computer code

Data collection	No commercial and custom code were used for data collection.
Data analysis	The pipeline of πn and πs estimation are homemade R scripts and it is deposited in Zenodo93 (https://doi.org/10.5281/zenodo.5136527). All softwares used for analysis in this study have been described in Methods. The software used in this study: 3D-DNA v180114, Admixtools v4.1, Admixture v.1.3.0, ANNOVAR relased_2019-10-24, AUGUSTUS v3.3.2, BLASTP 2.7.1+, Bowtie2 v2.3.5, BUSCO v3.0.1, BWA v0.7.17, Canu v1.6, GATK v.3.8, ggplot2 v3.2.1, HapCUT2 v1.1, HaploMerger2 v20180603, HiCExplorer v2.1.1, JCVI v0.8.4, Juicer v1.6.2, KING v2.2.4, LoRDEC v0.7, ltr_finder v1.07, LTR_retriver v2.6, ltrharvest v1.5.10, MAKER v2.31.8, MCMCtree v 4.9i, MCScanX, Orthofinder2 v2.3.3, PASA v2.3.3, Pfam , picard v.2.5.0, Plink v1.90p, PopLDdecay 3.40, Samtools v1.7, SMC++ v1.15.3, SNAP v2013-11-29, SnpEff v.4.3t, SNPhyol v20180901, STAR 2.5.3a, Stringtie v1.3.3b, TASSEL5.0 v5.2.52, TBtools v0_6741, Trimmomatic v0.36, Trinity v2.8.3, VCFtools v0.1.15, Long Ranger v2.2.2, GMAP version 2017-11-15, MUMer 4.0.0beta2, Assemblytics webtools, MIRA v4.0.2, MITObim v1.9.1, MAFFT v7.429, trimAl v1.4.rev22, IQ-TREE v1.6.10, FastTree2 v2.1.10, MUSCLE v3.8.31, SweepFinder2 v2.1.0, ANGSD v0.934, Stairway Plot 2 v2.1.1, fastsimcoal2 v 2.7.0.2; The script used in this study: https://github.com/isaacovercast/easySFS/blob/master/easySFS.py

For manuscripts utilizing custom algorithms or software that are central to the research but not yet described in published literature, software must be made available to editors and reviewers. We strongly encourage code deposition in a community repository (e.g. GitHub). See the Nature Research guidelines for submitting code & software for further information.

Data

Policy information about availability of data

All manuscripts must include a data availability statement. This statement should provide the following information, where applicable:

- Accession codes, unique identifiers, or web links for publicly available datasets
- A list of figures that have associated raw data
- A description of any restrictions on data availability

All raw sequencing data of DNA resequencing, 10X Genomic, Hi-C and RNA-seq are available at the National Center for Biotechnology Information database with a project ID of PRJNA747875. The monoploid reference and two haplotype assemblies are also deposited in NCBI with accession JAHYJY000000000, JAIUGD00000000, and JAIUGE000000000, respectively. The assembly and annotation of monoploid and haplotype genomes were uploaded into Mendeley database (https://data.mendeley.com/datasets/kggzfwpdr9/1) as well. VCF files that contain all clean SNPs were also uploaded to the Mendeley database (https://data.mendeley.com/datasets/v37bv5jt6g/1). Accession number or websites for public genomic data or sequencing data are listed in Supplementary Table 34.

Field-specific reporting

Please select the one below that is the best fit for your research. If you are not sure, read the appropriate sections before making your selection.

☒ Life sciences ☐ Behavioural & social sciences ☐ Ecological, evolutionary & environmental sciences

For a reference copy of the document with all sections, see nature.com/documents/nr-reporting-summary-flat.pdf

Life sciences study design

All studies must disclose on these points even when the disclosure is negative.

Sample size	Samples were selected to have enough representation from cultivated and wild accessions to investigate meaningful results.
Data exclusions	No data were excluded.
Replication	No replication was applied to gene expression, as the data were collected years ago or from public released data and more importantly, these data are good enough to support the findings in the study.
Randomization	This is not relevant to our study as all of the analyses followed published and well-established methods.
Blinding	The investigators were blinded to group allocation during data collection.

Reporting for specific materials, systems and methods

We require information from authors about some types of materials, experimental systems and methods used in many studies. Here, indicate whether each material, system or method listed is relevant to your study. If you are not sure if a list item applies to your research, read the appropriate section before selecting a response.

Materials & experimental systems

n/a	Involved in the study	
☒	☐	Antibodies
☒	☐	Eukaryotic cell lines
☒	☐	Palaeontology and archaeology
☒	☐	Animals and other organisms
☒	☐	Human research participants
☒	☐	Clinical data
☒	☐	Dual use research of concern

Methods

n/a	Involved in the study	
☒	☐	ChIP-seq
☒	☐	Flow cytometry
☒	☐	MRI-based neuroimaging
