# Supplementary material for: Two divergent haplotypes from a highly heterozygous lychee genome suggest independent domestication events for early and late-maturing cultivars
Source: Nat Genet. 2022 Jan 3;54(1):73–83. doi: 10.1038/s41588-021-00971-3 (PMC8755541; doi:10.1038/s41588-021-00971-3)
Supplement: Source Data Fig. 1 — Unprocessed gel for Fig. 6e. [file 41588_2021_971_MOESM5_ESM.pdf]

Supplementary Data files.

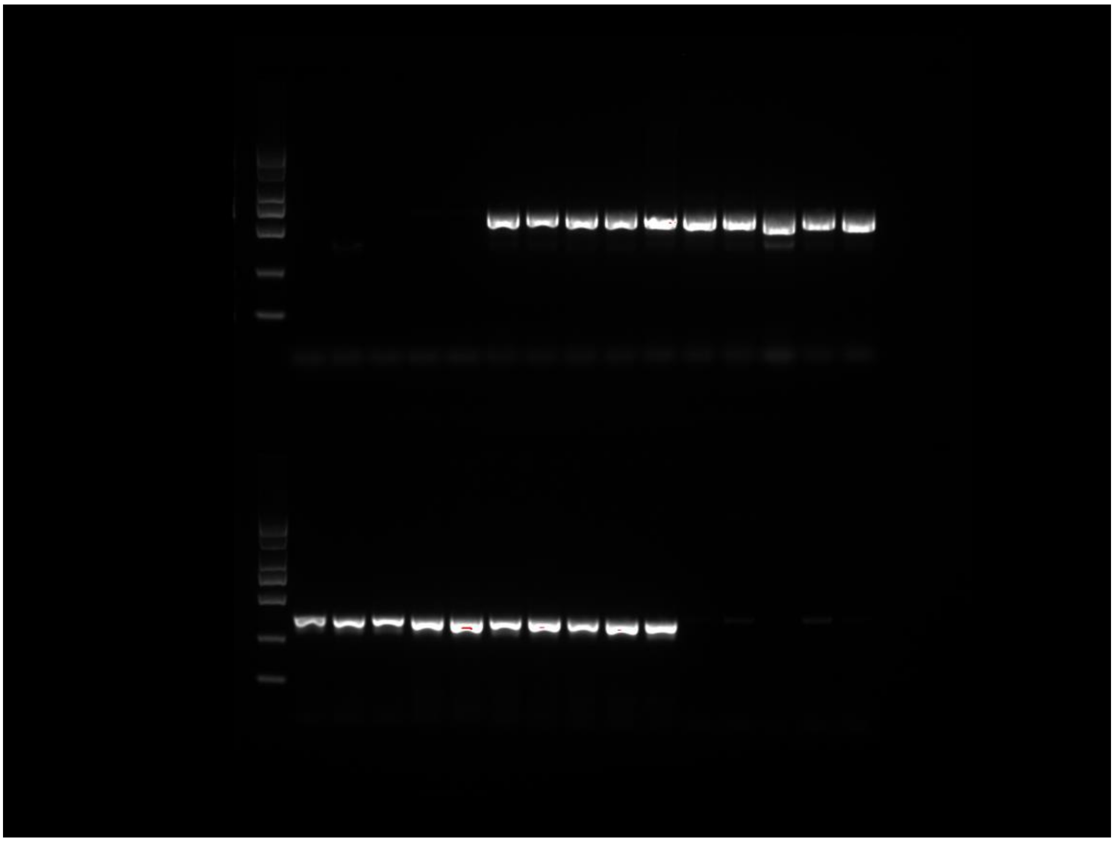

Source Data Fig. 1 | Uncropped scans of gels for the PCR of 3.7 kb deletion (Fig. 6e). Details in https://figshare.com/articles/figure/SourceFig1_tif/15049242.
