# Supplementary material for: Two divergent haplotypes from a highly heterozygous lychee genome suggest independent domestication events for early and late-maturing cultivars
Source: Nat Genet. 2022 Jan 3;54(1):73–83. doi: 10.1038/s41588-021-00971-3 (PMC8755541; doi:10.1038/s41588-021-00971-3)
Supplement: Supplementary file 4 — Peer Review Information [file 41588_2021_971_MOESM4_ESM.pdf]

natureresearch

Peer Review Information

Journal: Nature Genetics
Manuscript Title: Two divergent haplotypes from a highly heterozygous lychee genome suggest independent domestication events for early and late-maturing cultivars
Corresponding author name(s): Professor Rui Xia

Reviewer Comments & Decisions:

Decision Letter, initial version:

11th Aug 2020

Dear Professor Xia,

Your Article, "Two divergent haplotypes from a highly heterozygous lychee genome point to independent domestication events for early and late-maturing cultivars" has now been seen by 3 referees. You will see from their comments copied below that while they find your work of considerable potential interest, they have raised quite substantial concerns that must be addressed. In light of these comments, we cannot accept the manuscript for publication, but would be very interested in considering a revised version that addresses these serious concerns.

We hope you will find the referees' comments useful as you decide how to proceed. If you wish to submit a substantially revised manuscript, please bear in mind that we will be reluctant to approach the referees again in the absence of major revisions.

To guide the scope of the revisions, the editors discuss the referee reports in detail within the team, including with the chief editor, with a view to identifying key priorities that should be addressed in revision.

In this case, 1) all referees have identified substantial issues in the genome assembly, haplotype phasing, and other analyses that need to be significantly improved or clarified. Please address all referee points as thoroughly as you can. Please note that we would expect to see all technical points fully addressed as a condition of further consideration of your manuscript. 2) Please delete or re-phrase the statement on the origin of the Feizixiao cultivar "to please his favorite concubine".

We hope that you will find the prioritised set of referee points to be useful when revising your study. If

you choose to revise your manuscript taking into account all reviewer and editor comments, please highlight all changes in the manuscript text file. At this stage we will need you to upload a copy of the manuscript in MS Word .docx or similar editable format.

We are committed to providing a fair and constructive peer-review process. Do not hesitate to contact us if there are specific requests from the reviewers that you believe are technically impossible or unlikely to yield a meaningful outcome.

If revising your manuscript:

*1) Include a “Response to referees” document detailing, point-by-point, how you addressed each referee comment. If no action was taken to address a point, you must provide a compelling argument. This response will be sent back to the referees along with the revised manuscript.

*2) If you have not done so already please begin to revise your manuscript so that it conforms to our Article format instructions, available here.
Refer also to any guidelines provided in this letter.

*3) Include a revised version of any required Reporting Summary:
https://www.nature.com/documents/nr-reporting-summary.pdf
It will be available to referees (and, potentially, statisticians) to aid in their evaluation if the manuscript goes back for peer review.
A revised checklist is essential for re-review of the paper.

Please be aware of our guidelines on digital image standards.

You may use the link below to submit your revised manuscript and related files:

[REDACTED]

Note: This URL links to your confidential home page and associated information about manuscripts you may have submitted, or that you are reviewing for us. If you wish to forward this email to co-authors, please delete the link to your homepage.

If you wish to submit a suitably revised manuscript we would hope to receive it within 6 months. If you cannot send it within this time, please let us know. We will be happy to consider your revision so long as nothing similar has been accepted for publication at Nature Genetics or published elsewhere. Should your manuscript be substantially delayed without notifying us in advance and your article is eventually published, the received date would be that of the revised, not the original, version.

Please do not hesitate to contact me if you have any questions or would like to discuss the required revisions further.

Nature Genetics is committed to improving transparency in authorship. As part of our efforts in this direction, we are now requesting that all authors identified as ‘corresponding author’ on published papers create and link their Open Researcher and Contributor Identifier (ORCID) with their account on

the Manuscript Tracking System (MTS), prior to acceptance. ORCID helps the scientific community achieve unambiguous attribution of all scholarly contributions. You can create and link your ORCID from the home page of the MTS by clicking on ‘Modify my Springer Nature account’. For more information please visit please visit www.springernature.com/orcid.

Thank you for the opportunity to review your work.

Sincerely,

Wei

Wei Li, PhD
Senior Editor
Nature Genetics
1 New York Plaza, 47th Fl.
New York, NY 10004, USA
www.nature.com/ng

Reviewers' Comments:

Reviewer #1:
Remarks to the Author:
This paper describes a highly heterozygous lychee genome consisting of two clearly divergent haplotypes. Together with 72 resequenced cultivated and wild lychee accessions, the authors infer that the de novo assembled genome originated from a hybrid between two independently domesticated accessions with opposite features of the fruit-maturation period. Also, in the hybrid genome, alleles from different haplotypes may express differently in the same tissue. Some of the expression differences may be correlated with the early or late maturation period of lychee fruits. I have read this paper with great interest. In general, the paper is well written, and the data and results are clearly presented. However, there are still a few points that need to be clarified, in my opinion.

In the genome (haplotype) assembly and annotation, it is not entirely clear to me how the two haploid genomes were annotated. According to the gene prediction results, the two haplotypes have exact same gene numbers on each chromosome as the reference genome (Suppl. Table 8). However, the two gene sets each have over 70% genes with indels and SNPs, over 90% of which would change the amino acid sequences (Line 364-372). Therefore, I was wondering whether there would be any non-sense SNPs/indels or structural variations between the two haploid genomes, because of the independent domestication history of the two haploid genomes as claimed in the paper. For example, later in the paper, there is a missing intergenic region, which may be attributed to the maturity time.

Lines 359-361, the comparable mapping coverage to both HY and HH of some EMC accessions, would not suggest they are recent hybrids. Actually, one would expect a half-half read coverage for any self-mapping in a diploid genome, and this is exactly the case for the read mapping of "Feizixiao" because

the two haploid genomes are from the accession. Other EMC accessions may have a similar genetic background as "Feizixiao," so they have comparable mapping coverage when using "Feizixiao" as the reference. For example, they may be descendants of "Feizixiao," or they are also hybrids of LMC and EEMC, as shown in Fig. 3A.

For the timing of hybridization, I am not sure why the HY haplotype and HH haplotype need to have diverged from the common ancestor of lychee and longan (Line 380-384). In Line 226-228, the divergence between HNW and YNW has been estimated at 1.89 Mya (1.24 Mya in Line 623-624 though), so this is probably the earliest when the two haplotypes originated. There might be gene flow between the two populations, so the divergence between the two haplotypes could be inferred using the phylogenetic tree with single-copy orthologs as in Suppl. Fig. 15.

In the analyses of differential expression alleles, it is not entirely clear how the authors distinguished RNA-Seq reads from different alleles. Although a figure in the Methods suggests that only uniquely mapped reads with allele-specific SNPs are considered, mapping tools may tolerate mismatches that complicate the procedure, so it would be great if the approach could be discussed in somewhat more detail and caveats mentioned.

Fig. 4D does not show the differences of SNP densities for EEAs and DEAs in various gene features (Line 420-423), but just SNP densities in various gene features.
The authors state that indicating that DEAs (differentially expressed alleles) were under greater purifying selection pressure than were EEAs (evenly expressed alleles) (Fig. 4C) (lines 413-418). I'm not sure I understand. I would intuitively assume that purifying selection would keep expression of the alleles the same, unless the expression is different from the start (since the 'merging' of the two subgenomes) and this has to be maintained (is this what the authors mean?), while positive selection could be responsible for different alleles having different expression, like if you would compare duplicated genes with identical functions, and positive selection on one copy gives it a different function.

According to the authors (section "Flowering-related genes in lychee"), flowering time is the determinant of fruit maturation in lychee, and there is a tandem expansion of SVP genes in lychee and longan. Knocking out the SVP genes in peach would result in the evergrowing mutant in a certain environment. This may indeed suggest possibly adaptation – or at least a link - to climates, but I think this would naturally lead to another question of whether there is any copy number variation of the SVP genes among different cultivated and wild lychee accessions? Also, is there any copy number difference for the HH and HY haplotypes in the "Feizixiao" accession?

In the section on 'Cultivation history of lychee', it is not entirely clear to me what is meant with second (and higher-order) relationships? The authors write that 'estimates of the level of relationship' were obtained using the KING software and reflect the level of shared heterozygous or homozygous haplotype blocks. But what does 'level' mean? I could not find this in the manuscript.

Reviewer #2:
Remarks to the Author:
The authors sequenced the genome of lychee and re-sequenced 72 accessions which allowed the to obtain genome wide SNPs. They use resulting information to investigate demography, domestication

and to look for evidence of selection and allele specific gene expression. Overall the study is intended as resource for breeding.

At some level the study is novel as it provides information for a tree crop that has not been studied extensively using genomics. However it was difficult for me to see the argument for general interest as the genome information is not really used to generate novel biological insights.

The pop gen analysis also needs help

Line 194 : the simple ratio of numbers of non-syn and syn polyorphims is likely affected by the different samples sizes of the three datsets compared here. The authors should calculated pi_n/pi_s, which allows to compare datasets with different sample sizes.

Line 204 (and 212): Figure 2A is not a “phylogenetic tree”. This is because meotic recombination affected the genealogical relationships between these accessions such that no single tree can explain the evolutionary history of the sample. The this tree as a graphical representation of genetic distances between the 72 resequenced accessions.

Line 227 : it would be good to have an independent estimate of this split time done using another method (either msmc2, fastsimcoal2, dadi, or Relate). This would confer more confidence in this important result.

Line 244: Could it be that those differences also arise because of difference in levels of self-fertilization in natural populations?

Figure 3A: how exactly are those kinship relationships calculated?

l.279: what do they authors mean by “neutral” mutations? Synonymous polymorphisms? It would seem appropriate to use synonymous nucleotide diversity, which allows the comparisons of diversity between samples of different sample sizes.

L 316-318: Having more pronounced LD pattern in the cultivars versus the wild populations alone could simply result from repeated bottlenecks during domestication.

l. 320:On “time of cultivation”? Has this been done using archeological records?

l. 333: All arguments based on changes in proportion of “high-impact mutation” would benefit for re-checking for effect of different sample sizes.

l. 387: This first sentence is not clear: what is meant by “adaptability” here? Is it used in the sense of Evolvability (Payne and Wagner2018).

l.395 15000 DEA out of how many genes, 30000? That is every second gene is a DEA? Is it still possible to do meaningful enrichment analysis with this large number of DEA?

l. 416/417: using 0.1 as a cutoff for “strong purifying selection” is arbitrary -what is the justification.

l. 433 I don’t understand what evidence the authors have that DEA are time or tissue specific.

l. 542: just having Ka/Ks > 1 is not enough evidence for stating that the gene was targeted by positive selection. The authors need to use additional analyses to provide significant evidence using selective sweep detection methods like Sweepfinder2 (http://degiorgiogroup.fau.edu/sf2.html). This applies to other parts of the manuscript.

l. 550: What is the value of reporting non significant associations?

Reviewer #3:
Remarks to the Author:
Hu et al. report a genome assembly for the specialty fruit crop lychee and utilize resequencing and comparative genomics to uncover the domestication history and genomic basis for flowering time variation in lychee, respectively. The authors produced a fully phased, chromosome-scale assembly of the highly heterozygotic lychee genome and uncovered extensive allele specific expression. They provide evidence for two domestication events in lychee and identified homologs of flowering time genes in Arabidopsis that may be related to flowering time differences between early and late maturing cultivars. I read this paper with interest, but I have a few concerns that should be addressed before publication.

1. I have some concerns about the genome assembly and haplotype phasing. It is unclear based on the results and methods how one haplotype was extracted from the assembly and used for Hi-C anchoring. Based on the Hi-C contact matrix and BUSCO score, it seems like HaploMerger2 successfully binned the genome into two haplotypes, but each bin of contigs likely represents a chimera of the HY and HH haplotypes stitched together. Based on the raw PacBio assembly size, both the HY and HH haplotypes were assembled for ‘Feizixiao’, so couldn’t these be used as a basis for haplotype phasing? This would give the authors a better sense of copy number variations, TE polymorphisms, large-scale rearrangements, and other differences between the HY and HH haplotypes in ‘Feizixiao’ compared to using Illumina data alone. This may also improve alignment of reads for the resequencing data and analysis of differentially expressed alleles. If the within genome heterozygosity is truly ~2.2%, the HiC data should be sufficient for phasing HY and HH from the original assembly, especially if the authors use ALLHiC (which was created by several coauthors of this manuscript). There are very few phased haplotype genome assemblies for plants and additional analyses using an improved assembly would strengthen the manuscript.
Details on haplotype phasing and verification are vague. The authors state HapCUT2 was used for phasing and ‘eventually, 15 pairs of homologous chromosomes were obtained’. If the original haploid assembly was a chimera, wouldn’t these chimeric regions be carried over in the resulting HY and HH haplotypes? I am unsure why aligned Illumina data was used for phasing when both alleles were already assembled in the raw PacBio contigs.

2. The results on maturation time are interesting, but the finding that CONSTANS genes contribute to maturity time is not well supported. It is not surprising that the authors found no statistically

significant GWAS peaks for flowering time given the low sample size (62 based on the methods) and significant population structure between the wild and independently domesticated cultivars. The authors identified one flowering time related gene from the top 20 GWAS peaks, but this is not unexpected since the genome contains so many flowering genes. The heterozygous deletion in one haplotype is interesting, but because both alleles are expressed, it's difficult to say if this plays a role in flowering time variation. Additional evidence would help strengthen the claim that this gene/gene pair is involved in maturation.

3. The manuscript is quite long overall, and some sections contain extraneous text that distracts from the most meaningful findings. For instance, the conservation of a VRN1-like gene cluster across eudicots is interesting but does not yield meaningful results in lychee. The number of VRN1- like genes is similar in lychee and other genomes and many of these genes are expressed across diverse tissues, so it is unclear what role this gene cluster may play in flowering time regulation compared to other species.

Minor:
It is difficult to distinguish the wild from cultivated, maturity classes, and country of origin of the accessions in Figure 2A. I like the concept of using different fruit and leaf colors, but it is hard to interpret.

Lychee is a specialty fruit and I suspect many readers will be unfamiliar with the different cultivars of lychee and their distinguishing characteristics. It may be helpful to include pictures of representative cultivar groups for the wild, extremely early, early, and late-maturing groups in one of the figures.

Author Rebuttal to Initial comments

Dear Editors and Reviewers,

Thank you for your time and effort for handling our manuscript. We are pleased that the Reviewers and the Editors believe that our work is of great interest and provide new, valid information to the plant genomics field. We are grateful for the invaluable comments and suggestions from the three Reviewers, which were very helpful for the revision and improvement of our manuscript. We have substantially revised our manuscript by conducting additional sequencing, redoing our haplotypic genome assembly, reanalyzing many datasets, and redrawing or reorganizing a few figures. Below we provide a brief summary of the revisions made to best address the Reviewer's concerns and questions.

The major revisions are as follows.
(1) To address the concern expressed by both the Reviewers and Editors on the haplotype phasing, we first performed additional genome sequencing using the 10X Genomics platform to better assess the accuracy of haplotype phasing and to improve the identification of haplotypic SNP blocks.

(2) For further improvement of haplotype genome quality, we adopted a completely new strategy to assemble haplotypic genomes. A new set of haplotypes with significantly better SNP haplotyping consistency was obtained and used for downstream data reanalyses, including new variant analyses.

(3) We were able to obtain more haplotypic genomic information, including structural variation, copy number variation, and large-scale rearrangements, by comparing the new haplotypic genomes. Using the new and improved haplotype assemblies, we redid most of the analyses related to the differential expression of allelic genes.

(4) For the population genetic analyses, we have redone many analyses or added additional analyses, as reviewer #2 suggested we solidify our results. In order to carry out the revisions we developed new software to calculate the requested π_n / π_s values (coined as PiNSiR, π_n π_s analysis in R). The software is available in github at https://github.com/jsalojar/PiNSiR. Additionally, we found that inbreeding has a strong impact on demographic analyses using coalescent approaches, such as pairwise sequentially Markovian coalescent (PSMC), Stairway plot, and SMC++ models, and we here demonstrate its effect for PSMC. We summarize the result in a new supplementary note where we simulate the effect of inbreeding with an R code developed for the purpose, and we provide a practical approach for compensating for it.

Our point-by-point responses are preceded by ">>>" in blue, with the Reviewers' original comments in black.

##

Key priorities identified by the Editors:

1) all referees have identified substantial issues in the genome assembly, haplotype phasing, and other analyses that need to be significantly improved or clarified. Please address all referee points

as thoroughly as you can. Please note that we would expect to see all technical points fully addressed as a condition of further consideration of your manuscript.
2) Please delete or re-phrase the statement on the origin of the Feizixiao cultivar “to please his favorite concubine”.

>>>Thank you for identifying the first key priority and important second point. We apologize for the confusion or inclarities in our original manuscript.

Response to the priority #1:
Reviewer #3 was concerned with the haplotype phasing strategy we previously used, and he/she recommended to use AllHiC for direct haplotype phasing and contig anchoring simultaneously. This was a pertinent suggestion, as we had indeed tested AllHiC when we started to assemble the initial genome, but unfortunately AllHiC did not provide a good result at the time. To better address the reviewer's concern, we (including the main developers of the AllHiC tool, Dr. Xingtan Zhang and Dr. Haibao Tang) repeated the work using the AllHiC strategy (as the reviewer #3 suggested), but in a much more thorough way. We still obtained poor results, which we concluded to mean that AllHiC was not appropriate for our data. In a best-case scenario, assembled contigs assigned to different homologous groups (HGs) can be separated into contig groups (in the partition step of AllHiC), showing good synteny to corresponding chromosomes. But in our reanalysis, as shown in the figure below, although assembled contigs can be well separated into 15 HGs, most contig groups in a HG (y axis) do not show a good 2-to-1 relationship to the corresponding chromosomes (x axis) of the reference genome. This is likely due to our HiC data not effectively distinguishing preassembled haplotypic contigs, as was suspected by the referees as well. Among the 15 chromosomes, only Chr7 and its HGs showed a good 1-2 relationship. Most of the other HGs represent partial or incomplete 2-to-1 correspondences to their related chromosomes, for instance, Chr1, 8, 9, 11, 14. For some HGs, only 1-to-1 relationships were detected, including Chr5, 6 10, 15, suggesting that the two haplotypes of these chromosomes could not be possibly distinguished by AllHiC either.

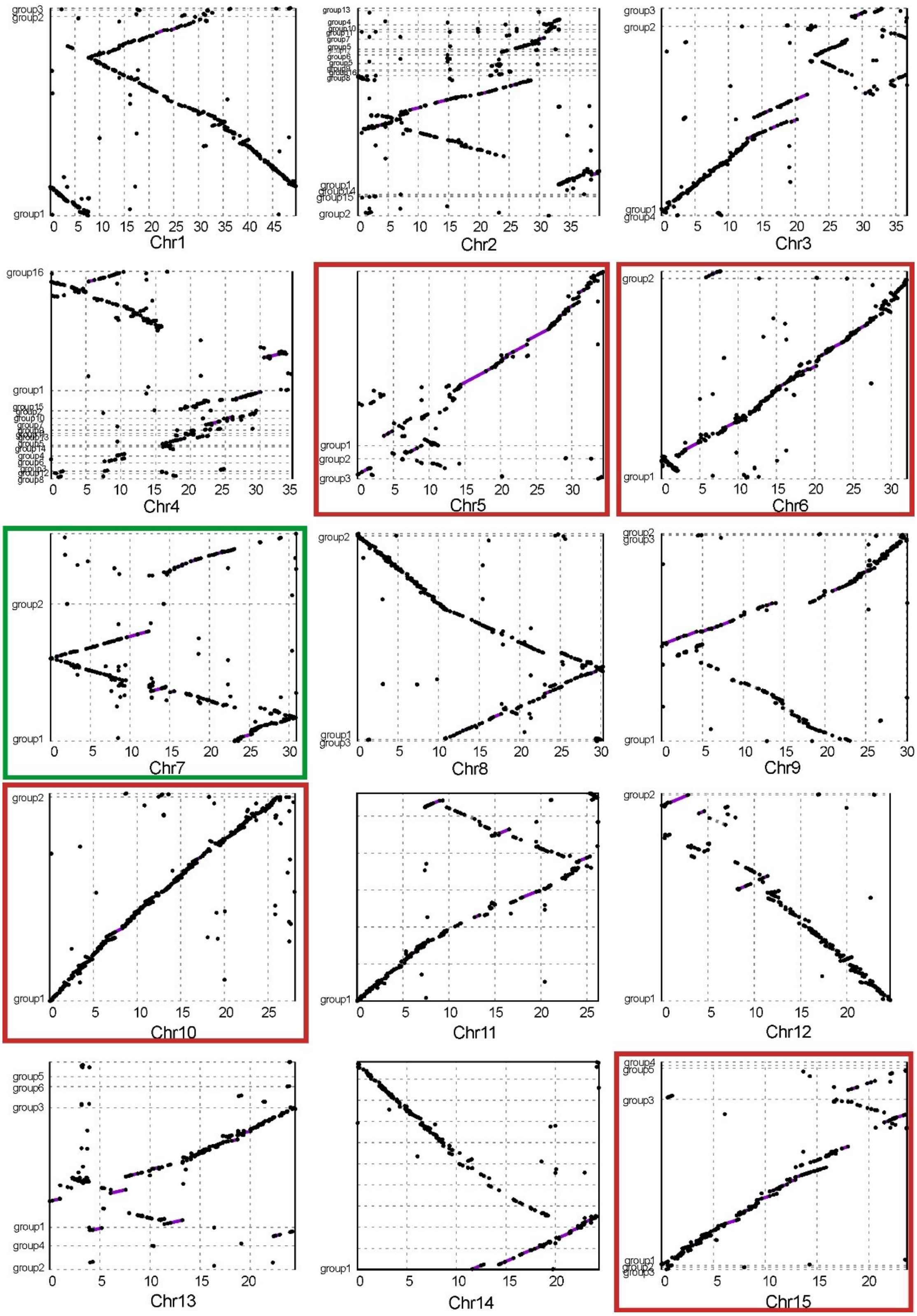

To ascertain why AllHiC did not work in our case, we carefully evaluated our results, and found that the main reason for the failure of AllHiC was because the assembly tool CANU likely introduced chimeric contigs in the first (error correction) step of contig assembly. CANU is one of the most popular and effective tools used for assembling contigs from raw PacBio long reads, and we still preferred to employ it given our experience with various assembly options available. The

problem of chimeric assembly using CANU has also been demonstrated and reported in our original AllHiC paper (Zhang et al., 2019, “Assembly of allele-aware, chromosomal-scale autopolyploid genomes based on Hi-C data”, Nature Plants). Once the chimeric contigs are introduced, it is almost impossible to resolve them in the following steps. Although the CANU-trio binning algorithm is a haplotype phasing method, it requires parental genome information, which was lacking in our case. Therefore, more data were obtained and a new phasing approach was employed, as described below.

To evaluate the accuracy of our haplotype phasing, we resequenced the ‘Feizixiao’ genome using the 10X Genomics platform (10X data) to obtain long-range linked-reads data with 100X coverage. Using the 10X data, we found that our original phasing accuracy of SNPs using HiC data was around 90%, which we considered to have been reasonable. For sequence blocks with SNP quantity >50 per block, the consistency between each haplotype and the 10X data is between 86-90% (as shown in the table below).

**SNP quantity per block**	**block num**	**total SNPs**	**Consistency (%)**	
			**hap1**	**hap2**
>=1	33791	5,827,504	98.9	98.2
>=5	3503	5,790,274	92.2	90.1
>=10	2217	5,782,172	89.4	87.3
>=20	1716	5,775,566	88.0	85.9
>=50	1522	5,769,915	87.9	86.0
>=100	1449	5,764,489	88.1	86.5
>=200	1346	5,749,365	88.8	87.3
>=500	1161	5,687,591	89.6	88.4
>=1000	956	5,536,842	90.4	89.4
>=2000	716	5,186,592	91.3	90.4

However, inspired by the reviewers’ suggestions and a recent strategy our coauthor Dr. Xintan Zhang used in another study (Zhang et al., 2020, Genomes of the banyan tree and pollinator wasp provide insights into fig-wasp coevolution, Cell), we decided to adopt a similar approach for an all-new haplotype phasing. SNP blocks identified from HiC data and 10X data are used for direct phasing of raw PacBio long reads before the step of CANU assembly. After the phasing, reads

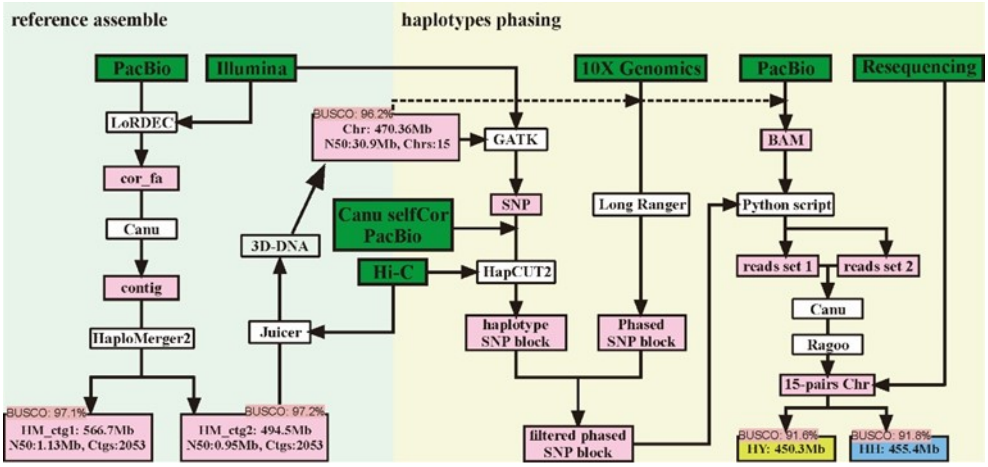

separated into two groups were subsequently assembled by CANU independently, after which assembled contigs were scaffolded using RaGOO with the guidance of the reference genome, achieving 15 pairs of homologous chromosomes. The workflow is shown below, and in Supplementary figure 1 as well. Detailed steps are described in Methods.

In this way, we were able to obtain two new, high-quality haplotypic genome sequences. Although their completeness dropped slightly, the accuracy of haplotype phasing was improved considerably. We consider this tradeoff reasonable, since the strategy involved separating raw PacBio reads into two groups for haplotype CANU assembly, where the amount of PacBio reads used for each haplotype assembly was almost half of the total reads. Accuracies of the new haplotype assemblies compared to 10X data were increased to 93-96% for genomic blocks with >50 SNPs (shown in the table below).

SNP quantity per block	block num	total SNPs	Consistency (%)	
			hap1	hap2
>=1	10925	6,683,700	98.2	97.7
>=5	3454	6,669,827	95.7	95.6
>=10	2205	6,661,877	94.1	94.0
>=20	1717	6,655,451	93.5	93.3
>=50	1530	6,650,155	93.4	93.3
>=100	1460	6,644,906	93.6	93.5
>=200	1367	6,631,872	94.0	93.9
>=500	1188	6,571,728	94.9	94.8
>=1000	1001	6,433,426	95.6	95.5
>=2000	762	6,087,071	96.3	96.2

Based on our overall significantly improved SNP haplotyping consistency and genome contiguity, we have now decided to use these improved haplotypic genomes for subsequent analyses and have updated all results accordingly. In summary, the improved haplotype-resolved genomes are now based on an integrated assembly and phasing pipeline utilizing a mixture of PacBio, Illumina, 10X Genomics as well as HiC data.

BUSCO analysis of haplotypic genome assemblies (raw genome sequence)

Description	Chr		HY		HH	
	Number	Per. (%)	Number	Per. (%)	Number	Per. (%)
Complete BUSCOs (C)	2041	96.2%	1943	91.6%	1948	91.8%
Complete and single-copy BUSCOs (S)	1930	91.0%	1895	89.3%	1905	89.8%
Complete and duplicated BUSCOs (D)	111	5.2%	48	2.3%	43	2.0%
Fragmented BUSCOs (F)	28	1.3%	32	1.5%	34	1.6%
Missing BUSCOs (M)	52	2.5%	146	6.9%	139	6.6%
Total BUSCO groups searched	2121	100.0%	2121	100.0%	2121	100.0%

BUSCO analysis of genome annotation (protein coding sequences)

Description	Chr		HY		HH	
	Number	Per. (%)	Number	Per. (%)	Number	Per. (%)
Complete BUSCOs (C)	2010	94.8%	1817	85.7%	1816	85.6%
Complete and single-copy BUSCOs (S)	1152	54.3%	1765	83.2%	1770	83.5%
Complete and duplicated BUSCOs (D)	858	40.5%	52	2.5%	46	2.2%
Fragmented BUSCOs (F)	41	1.9%	75	3.5%	77	3.6%
Missing BUSCOs (M)	70	3.3%	229	10.8%	228	10.7%
Total BUSCO groups searched	2121	100.0%	2121	100.0%	2121	100.0%

Response to the priority #2:

We have reworded the sentence to "In the ancient Tang Dynasty, roughly 1300-1100 years ago, the Emperor set up a courier service with fast horse relays to transport fresh lychee from southern China to the imperial court because of the prodigious flavor of the spoilable fruit." at Lines 76-79.

Reviewers' Comments:

Reviewer #1:

Remarks to the Author:

This paper describes a highly heterozygous lychee genome consisting of two clearly divergent haplotypes. Together with 72 resequenced cultivated and wild lychee accessions, the authors infer that the de novo assembled genome originated from a hybrid between two independently domesticated accessions with opposite features of the fruit-maturation period. Also, in the hybrid genome, alleles from different haplotypes may express differently in the same tissue. Some of the expression differences may be correlated with the early or late maturation period of lychee fruits. I have read this paper with great interest. In general, the paper is well written, and the data and results are clearly presented. However, there are still a few points that need to be clarified, in my opinion.

>>> Thank you; we greatly appreciate these positive comments.

In the genome (haplotype) assembly and annotation, it is not entirely clear to me how the two haploid genomes were annotated. According to the gene prediction results, the two haplotypes have exact same gene numbers on each chromosome as the reference genome (Suppl. Table 8). However, the two gene sets each have over 70% genes with indels and SNPs, over 90% of which would change the amino acid sequences (Line 364-372). Therefore, I was wondering whether there would be any non-sense SNPs/indels or structural variations between the two haploid genomes, because of the independent domestication history of the two haploid genomes as claimed in the paper. For example, later in the paper, there is a missing intergenic region, which may be attributed to the maturity time.

>>> Thank you for raising this question. We performed a genome-wide calculation. There are indeed non-sense SNPs/indels, which account for ~2.6% (8,292/319,125) of all the nonsynonymous SNPs, and these occur in ~9.2% (2934/31,896) of all annotated genes. A GO functional analysis revealed that those genes with non-sense SNPs/indels were significantly enriched in biological processes related to defense responses (see table below). We added this information to the revised manuscript at Lines 382-384.

Top 20 GO enriched terms ("Biological process") for genes with non-sense SNPs/indels

GO_Name	GO_ID	HitsGenesCounts InSelectedSet	corrected p-value (BH method)
detection of other organism	GO:0098543	137	0
response to other organism	GO:0051707	1083	0
defense response to bacterium, incompatible interaction	GO:0009816	191	0
response to external biotic stimulus	GO:0043207	1083	0
innate immune response	GO:0045087	681	0
detection of bacterium	GO:0016045	137	0
detection of external biotic stimulus	GO:0098581	160	1.54E-13
host programmed cell death induced by symbiont	GO:0034050	252	1.54E-13
defense response	GO:0006952	1217	1.54E-13
detection of biotic stimulus	GO:0009595	160	1.54E-13
response to bacterium	GO:0009617	728	1.54E-13
plant-type hypersensitive response	GO:0009626	252	3.6E-13
immune response	GO:0006955	763	4.16E-13
defense response to other organism	GO:0098542	832	5.41E-13
defense response to bacterium	GO:0042742	612	7.93E-13
response to biotic stimulus	GO:0009607	1099	1.28E-12
defense response, incompatible interaction	GO:0009814	342	2.04E-12
defense response by cell wall thickening	GO:0052482	117	1.53E-11
defense response by callose deposition in cell wall	GO:0052544	117	1.53E-11
response to oomycetes	GO:0002239	200	3.61E-10
callose deposition in cell wall	GO:0052543	125	4.11E-10
defense response by callose deposition	GO:0052542	134	5.1E-10
detection of stimulus	GO:0051606	239	6.58E-10
immune system process	GO:0002376	839	9.5E-10

Lines 359-361, the comparable mapping coverage to both HY and HH of some EMC accessions, would not suggest they are recent hybrids. Actually, one would expect a half-half read coverage for any self-mapping in a diploid genome, and this is exactly the case for the read mapping of "Feizixiao" because the two haploid genomes are from the accession. Other EMC accessions may have a similar genetic background as "Feizixiao," so they have comparable mapping coverage when using "Feizixiao" as the reference. For example, they may be descendants of "Feizixiao," or they are also hybrids of LMC and EEMC, as shown in Fig. 3A.

>>>Thank you for this concern. We agree that "a half-half read coverage would be expected for any self-mapping in a diploid genome". However, in our case, it was not self-mapping; instead we

mapped the resequencing data from different lychee varieties (both wild and cultivated) to the two haplotypes of ‘Feizixiao’ independently. For ‘Feizixiao’ itself, half-half coverage indeed came from self-mapping, but for all other varieties, their biased coverage between HY and HH was not due to self-mapping, but instead because of the diverse genetic backgrounds studied.

We also agree that the comparable mapping coverage of other EMC varieties suggested that they may have similar genetic backgrounds as ‘Feizixiao’, i.e., that they might be descendants of ‘Feizixiao’ or hybrids of LMC and EEMC, which is also supported by our inferred cultivation history for lychee varieties (Figure 3). We have revised a few words to make this information clearer at Lines 363-366.

For the timing of hybridization, I am not sure why the HY haplotype and HH haplotype need to have diverged from the common ancestor of lychee and longan (Line 380-384). In Line 226-228, the divergence between HNW and YNW has been estimated at 1.89 Mya (1.24 Mya in Line 623-624 though), so this is probably the earliest when the two haplotypes originated. There might be gene flow between the two populations, so the divergence between the two haplotypes could be inferred using the phylogenetic tree with single-copy orthologs as in Suppl. Fig. 15.

>>> Regarding the divergence time, as suggested, we did consider and experiment with using single-copy orthologs. The divergence time estimate obtained in this manner was ~4.6 Mya (below), which is not rational for a within-species split. We therefore only feel comfortable reporting our estimates based on actual Hainan and Yunnan wild population genomic data, given biasing factors in the approach outlined above such as deep fossil calibration points (far older than any within-species split in Sapindaceae) and unknown (and family-by-family variable) natural selection influences on single-copy ortholog sequences. On the latter point, our genome-wide SNPs are expected to be mostly neutral given the low percentage of gene space per megabase of DNA in the lychee assembly.

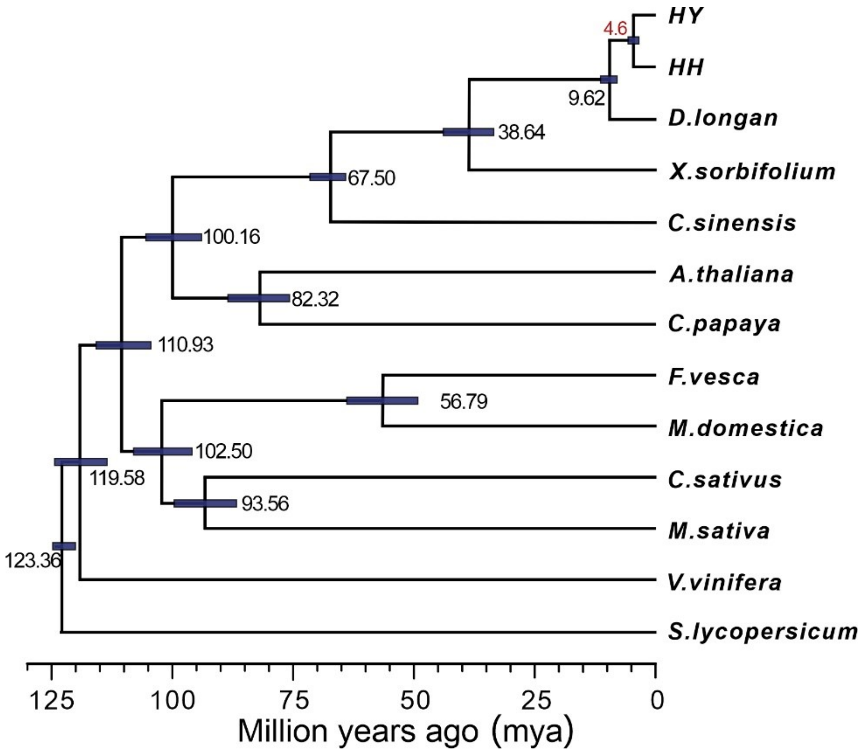

In the analyses of differential expression alleles, it is not entirely clear how the authors distinguished RNA-Seq reads from different alleles. Although a figure in the Methods suggests that only uniquely mapped reads with allele-specific SNPs are considered, mapping tools may tolerate mismatches that complicate the procedure, so it would be great if the approach could be discussed in somewhat more detail and caveats mentioned.

>>>We understand the inclarity in our previous manuscript version. We used the STAR aligner to map RNAseq reads to the merged HY and HH haplotype genome. We agree that it is possible that the tolerance of mismatches of the mapping tool may complicate the distinction between different alleles. However, using STAR and selecting uniquely mapping reads may still be the best strategy to minimize the possibility of mismapping. During RNAseq read mapping, the mismatch mainly came from significant SNP positions, which means fewer mismatches but less SNP difference. Therefore, the unique mapping with less mismatches should ensure that the majority of mapped RNAseq reads comes from each corresponding haplotypic genome.

To appraise this, we performed quick evaluations for a few different RNAseq data. We first aligned RNAseq reads to merged haplotypic genome sequences and only allowed unique matches, which separated RNAseq reads into two groups, reads aligned to HH and reads to HY. Then SNP positions were calculated based on the uniquely mapped RNAseq reads. After that, RNAseq-based SNPs for each haplotype were compared to the SNPs detected by HaploCUT2 from HiC and PacBio data to calculate the SNP consistency. Overall, the consistency value was >95%, suggesting a very low level of mismapping. Therefore, our expression calculations for allelic genes based on unique mapping of RNAseq reads can be considered of high accuracy.

We added a few words on this to the Methods section.

Tissue	Haplotypes	HapCUT2 SNPs detected by RNAseq data (same position)		Consistent SNPs between HapCUT2 and RNA-seq (same nucleotide)		Accuracy	
		HY	HH	HY	HH	HY	HH
aril	HY	149,108	6,920	148,966		95.5%	
	HH	6,597	162,615		162,475		96.0%
embryo	HY	255,262	17,152	254,950	16,786	93.6%	
	HH	17,153	251,279		250,969		93.5%
pericarp	HY	185,070	10,274	184,857		94.6%	
	HH	9,625	198,806	9,439	198,584		95.3%

Fig. 4D does not show the differences of SNP densities for EEAs and DEAs in various gene features (Line 420-423), but just SNP densities in various gene features.

>>>Thank you for catching this deficiency. The old Fig. 4D fails to show the SNP differences between EEAs and DEAs. We replaced old Fig. 4D with an updated figure, in which the difference of SNP densities between EEAs and DEAs was compared among different gene feature regions. DEAs have higher SNP densities in most of these regions except exons and ~2kb after the 3'-UTRs.

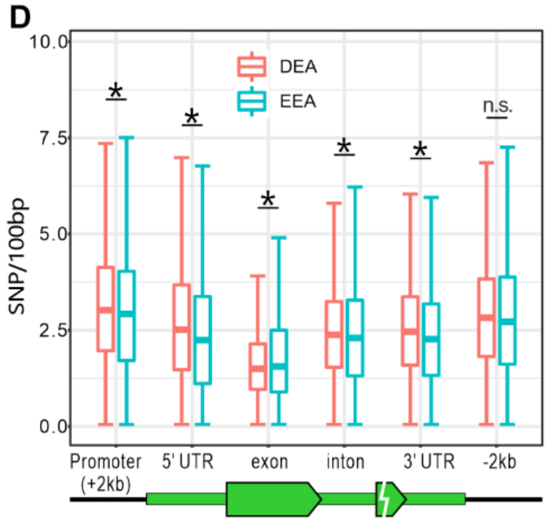

The authors state that indicating that DEAs (differentially expressed alleles) were under greater purifying selection pressure than were EEAs (evenly expressed alleles) (Fig. 4C) (lines 425-427). I'm not sure I understand. I would intuitively assume that purifying selection would keep expression of the alleles the same, unless the expression is different from the start (since the 'merging' of the two subgenomes) and this has to be maintained (is this what the authors mean?), while positive selection could be responsible for different alleles having different expression, like if you would compare duplicated genes with identical functions, and positive selection on one copy gives it a different function.

>>> Thank you for pointing out this inclarity. Indeed, haplotypic expression was likely to be different when ‘Feizixiao’ formed from hybridization between long-distinct LMC/HNW and EEMC/YNW haplotypes, and this differential expression was likely maintained. In fact, this genomic plasticity might explain the heterosis observed for ‘Feizixiao’, which has much improved fruit traits compared to wild lychee.

According to the authors (section "Flowering-related genes in lychee"), flowering time is the determinant of fruit maturation in lychee, and there is a tandem expansion of SVP genes in lychee and longan. Knocking out the SVP genes in peach would result in the evergrowing mutant in a certain environment. This may indeed suggest possibly adaptation – or at least a link - to climates, but I think this would naturally lead to another question of whether there is any copy number variation of the SVP genes among different cultivated and wild lychee accessions? Also, is there any copy number difference for the HH and HY haplotypes in the "Feizixiao" accession?

>>>Thank you, this is a good question. We double checked our original PacBio sequencing data of ‘Feizixiao’ and found that there is no copy number variation for SVP genes between the HH and HY haplotypes. Our 10X Genomics single-cell data also confirmed this result.

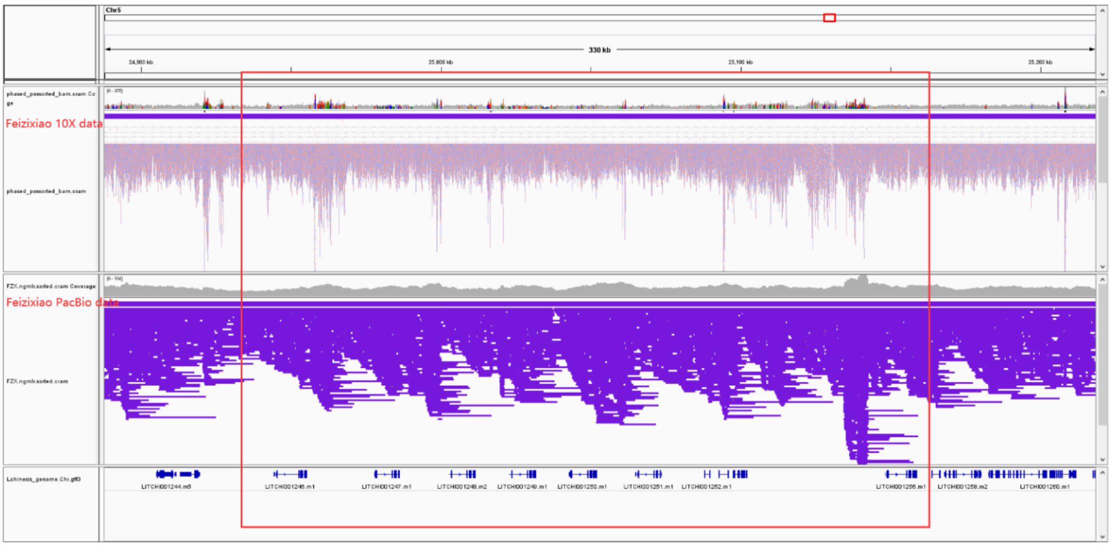

For other lychee variants (wild or cultivated lychee), we were not able to assess this because only Illumina short reads were sequenced for them, which were of too low coverage (average 13.72X) to reliably assemble the entire SVP cluster region.

In the section on ‘Cultivation history of lychee’, it is not entirely clear to me what is meant with second (and higher-order) relationships? The authors write that ‘estimates of the level of relationship’ were obtained using the KING software and reflect the level of shared heterozygous or homozygous haplotype blocks. But what does ‘level’ mean? I could not find this in the manuscript.

>>> We apologize, the nomenclature was an error made by a non-native English speaker. The order of relationship between individuals was estimated using KING-Robust algorithm

(Manichaikul et al. 2010), which estimates a kinship coefficient that is claimed to be independent of sample composition or population structure. The proper expression is the "order" of relationship, whether it is monozygotic twins, 1st order, 2nd order or 3rd order, where the order is determined by the kinship value ranges recommended in the KING online manual. We have now corrected this in the text and added explanation of the kinship coefficient into Materials and Methods.

References:

Ani Manichaikul, Josyf C. Mychaleckyj, Stephen S. Rich, Kathy Daly, Michèle Sale, Wei-Min Chen, Robust relationship inference in genome-wide association studies, Bioinformatics, Volume 26, Issue 22, 15 November 2010, Pages 2867–2873, https://doi.org/10.1093/bioinformatics/btq559

Reviewer #2:

Remarks to the Author:

The authors sequenced the genome of lychee and re-sequenced 72 accessions which allowed the to obtain genome wide SNPs. They use resulting information to investigate demography, domestication and to look for evidence of selection and allele specific gene expression. Overall the study is intended as resource for breeding.

At some level the study is novel as it provides information for a tree crop that has not been studied extensively using genomics. However it was difficult for me to see the argument for general interest as the genome information is not really used to generate novel biological insights.

>>> Thank you for your comment. Although lychee is not a globally popular fruit crop, it is one of the economically foremost fruit crops grown in eastern Asia, with >2000 years of recorded of cultivation history. It is the most agriculturally important crop in Sapindaceae, a huge family of flowering plants (including maple) that consists of 138 genera and ~2000 accepted species.

So far, in-depth genomics research on Sapindaceae species is sparse. Our thorough analyses of one lychee genome, diverse cultivars and wild populations will greatly broaden knowledge of the genomics of Sapindaceae species. Moreover, the highly heterozygous genome (2.27%) of 'Feizixiao' lychee enabled the assembly of two haplotypic genomes, which in fact represent two entirely separate wild lychee populations. We also demonstrated that extremely early and late-maturing cultivars were derived from these two populations via independent domestication events. Thus, we believe our work indeed provides valuable general insights into plant genomics.

The pop gen analysis also needs help

Line 194 : the simple ratio of numbers of non-syn and syn polyorphims is likely affected by the different samples sizes of the three datsets compared here. The authors should calculated pi_n/pi_s, which allows to compare datasets with different sample sizes.

>>> Thank you for this suggestion; we have now carried out all the analyses using π_n, π_s and their ratio. To do so, we developed a pipeline for calculating them from whole genome sequencing data, since we found the existing software cumbersome and very slow for whole genome sequencing data, taking over one week for one chromosome and one set of individuals. The implementation of

our pipeline is explained in the M&M and is made available in github (https://github.com/jsalojar/PiNSiR). We are using a combination of snpEff to predict functional impact of a position, ANGSD to calculate accurate estimates of site-wise π_n, π_s values under missing data, and we developed R scripts implementing parallel computation to calculate genome-wide averages. The pipeline also filters the genome for high-quality gene models. In its current state, assuming that the existing SnpEff and ANGSD files are present, the implementation calculates the π_n, π_s values within tens of minutes.

The revised analyses did not change the biological results. We can still see the effects of a cultivation bottleneck, but the tools help in assessing the effects more accurately, which is shown by the interesting results from non-synonymous diversity, where the cultivation bottleneck is clearly visible from purging of deleterious alleles.

Line 204 (and 212): Figure 2A is not a "phylogenetic tree". This is because meotic recombination affected the genealogical relationships between these accessions such that no single tree can explain the evolutionary history of the sample. The this tree as a graphical representation of genetic distances between the 72 resequenced accessions.

>>> Yes, this is correct. We have corrected the nomenclature and discuss genetic distances regarding the SNP tree instead.

Line 227 : it would be good to have an independent estimate of this split time done using another method (either msmc2, fastsimcoal2, dadi, or Relate). This would confer more confidence in this important result.

>>> Thank you for the recommendation. This suggestion made us look at the population histories in a very detailed manner. In order to increase the accuracy of our estimation we first estimated ancestral states for the nucleotides using longli, longan and rambutan sequencing data and ANGSD software. We then applied several demographic modeling approaches (pairwise sequentially Markovian coalescent, Stairway plot 2, and SMC++) to see if the results agree, as well as Fastsimcoal2 modeling suggested by the referee, using the estimated unfolded site frequency spectrum. In all cases the population divergence times showed surprisingly deep time scales on the order of millions of years.
Upon close inspection of the Ne trajectories we identified a consistent "shift" towards modern times in the Yunnan population. Since the inbreeding coefficient was high among the Yunnan population, and heterozygosity is the main factor used to define coalescence, we speculated that the discrepancy was due to an extensive amount of inbreeding or selfing occurring in the Yunnan

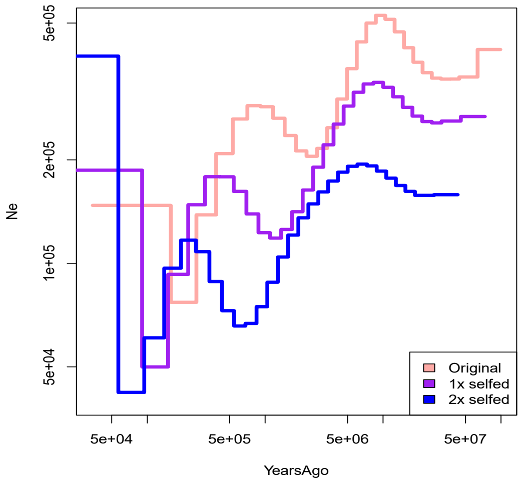

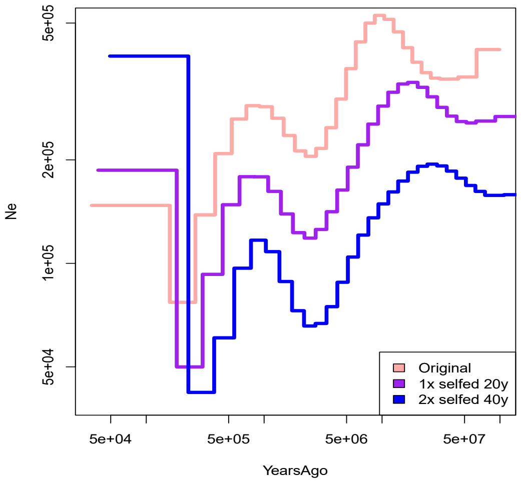

population. We simulated the effect of selfing – loss of heterozygosity due to Mendelian inheritance in the selfing individuals – using actual data from one individual from Hainan, and obtained population trajectories matching the effects seen in Yunnan lychee data (see the figure below extracted from our new supplementary note). Furthermore, since nucleotide diversity and mutation rate are linearly dependent, we corrected for the effect of inbreeding by adjusting the mutation rate of the individual. The adjustment matched the timing of the Ne trajectories, except that the overall effective population sizes were lower (corresponding to the drop of effective population size due to inbreeding).

With the actual data we noticed that adjusting the generation time of Yunnan to 3x the time in Hainan provided the best fit, corresponding to ~66% loss of heterozygosity due to inbreeding. This adjustment provided concordant trajectories for all demographic models. To avoid over-fitting, we carried out the estimation of inbreeding only with PSMC results and then observed the effect in Stairway plots and SMC++ results. See the plots below regarding differences for unadjusted (left) and adjusted (right) trajectories. The result and argumentation are summarized in our new supplementary note.

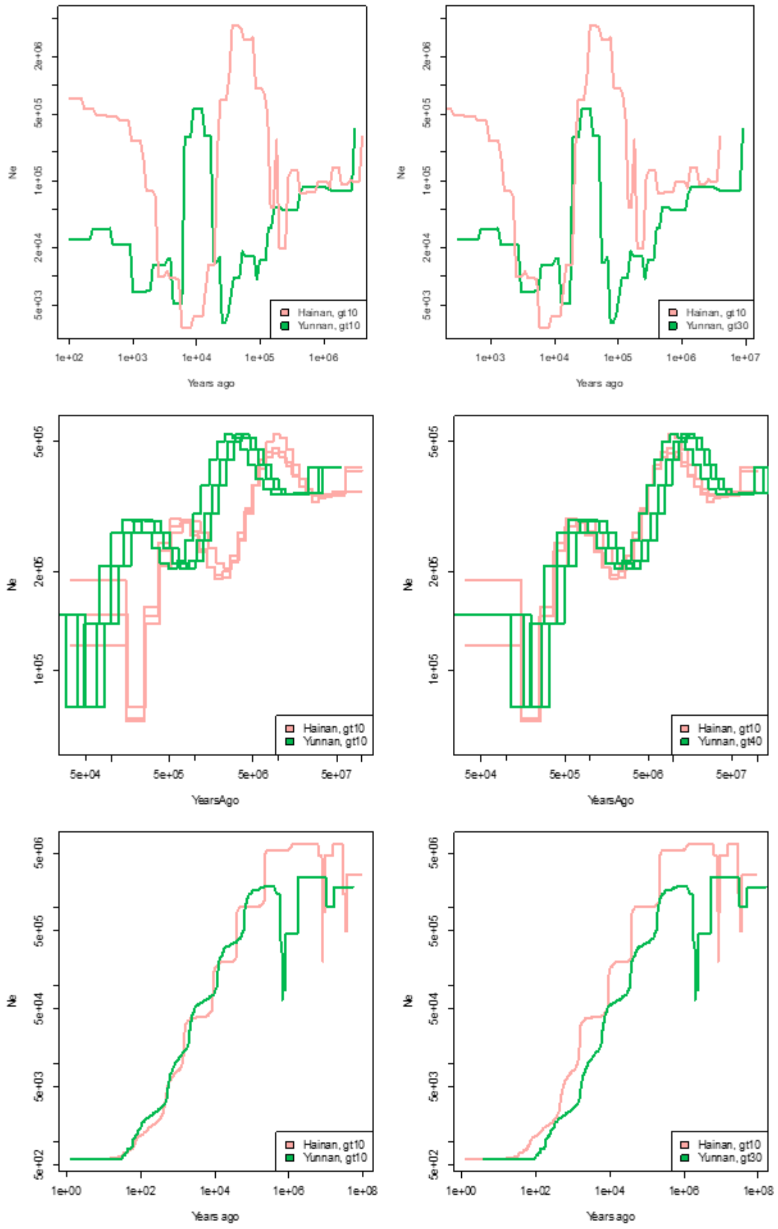

The SMC++ split time estimation does not allow different generation times since the 2d-site frequency spectrum is not properly estimated, so we therefore estimated the divergence time by overlaying the independently estimated trajectories. This gave us a divergence time of ~18,000 years (with considerable variation around the estimate due to uncertain generation times and mutation rates). This is much more realistic than the previously obtained date on the order of millions of years. Unfortunately, under this scenario, parameter estimation using Fastsimcoal simulations was not possible due to problems in estimating the 2-d site frequency spectrum. Regarding the models suggested by the referee, neither msmc2 nor Relate were applicable since they required phased genome data.

Line 244: Could it be that those differences also arise because of difference in levels of self-fertilization in natural populations?

>>> Yes. We assumed that selfing occurs because there's little opportunity for outbreeding due to low effective population size. It is of course also possible that the Yunnan populations have lower levels of self-incompatibility and therefore selfing is more common. We propose both scenarios in the revised manuscript at Lines 225-227.

Figure 3A: how exactly are those kinship relationships calculated?

>>> The order of relationship between individuals was estimated using the KING-Robust algorithm (Manichaikul et al. 2010, "Robust relationship inference in the presence of population substructure"), which estimates a kinship coefficient that is claimed to be independent of sample composition or population structure by calculating an estimator of the kinship coefficient based on the difference between shared heterozygosity and shared homozygosity (see Eq. 9 in the original publication). We have added this explanation in the Methods part of the manuscript.

References:

Ani Manichaikul, Josyf C. Mychaleckyj, Stephen S. Rich, Kathy Daly, Michèle Sale, Wei-Min Chen, Robust relationship inference in genome-wide association studies, Bioinformatics, Volume 26, Issue 22, 15 November 2010, Pages 2867–2873, https://doi.org/10.1093/bioinformatics/btq559

l.279: what do they authors mean by "neutral" mutations? Synonymous polymorphisms? It would seem appropriate to use synonymous nucleotide diversity, which allows the comparisons of diversity between samples of different sample sizes.

>>> We focused on intergenic mutations to estimate the neutral mutations. Compared to synonymous mutations, which are present only in gene coding regions, intergenic mutations are much more abundant, do not depend on the quality of the predicted models, and therefore permit more accurate estimation of π_s.

L 316-318: Having more pronounced LD pattern in the cultivars versus the wild populations alone could simply result from repeated bottlenecks during domestication.

>>> Yes, this is absolutely true, and has been observed in other crop species as well. We have altered the text accordingly at Lines 317-318.

l. 320:On "time of cultivation"? Has this been done using archeological records?

>>> We apologize, there is no record of the timing of the cultivation event. This was not accurately expressed. The text has been corrected to say "we split the cultivars into different categories according to their relatedness and cultivar origins"

l. 333: All arguments based on changes in proportion of "high-impact mutation" would benefit for re-checking for effect of different sample sizes.

>>> Thank you, we have now taken this into account by developing a novel pipeline for estimating π_n and π_s values from whole genome sequencing data, and share the pipeline through github. We have compared the estimated diversities to those computed for a large number of

species by another group (Chen et al 2017) and the values as well as the π_n/π_s ratio agree with outcrossing plants in general.

Reference:

Jun Chen, Sylvain Glémin, Martin Lascoux, Genetic Diversity and the Efficacy of Purifying Selection across Plant and Animal Species, Molecular Biology and Evolution, Volume 34, Issue 6, June 2017, Pages 1417–1428, https://doi.org/10.1093/molbev/msx088

l. 387: This first sentence is not clear: what is meant by "adaptability" here? Is it used in the sense of Evolvability (Payne and Wagner2018).

>>> Thank you. Yes, what we meant by "adaptability" is similar to the "evolvability" of Payne and Wagner (2018). We have revised the sentence and added the reference.

l.395 15000 DEA out of how many genes, 30000? That is every second gene is a DEA? Is it still possible to do meaningful enrichment analysis with this large number of DEA?

>>> Thank you for the suggestion. We performed GO enrichment analysis with the newly identified 13,517 DEAs. For Cellular component, plastid and thylakoid are the most significant enrichment GO terms. Photosynthesis (Biological process) and carboxylic acid binding (Molecular function) are enriched as well, suggesting that the DEAs are mainly associated with energy metabolism including photosynthesis, which may contribute to the growth vigor of lychee trees.

Class	GO_Name	GO_ID	HitsGenesCounts InSelectedSet	corrected p-value (BH method)
Molecular function	organic acid binding	GO:0043177	204	2.33E-04
Molecular function	carboxylic acid binding	GO:0031406	202	1.59E-04
Molecular function	antioxidant activity	GO:0016209	179	4.28E-04
Molecular function	metallochaperone activity	GO:0016530	30	0.004858635
Molecular function	disulfide oxidoreductase activity	GO:0015036	74	0.005233392
Cellular component	thylakoid part	GO:0044436	406	6.85E-12
Cellular component	thylakoid	GO:0009579	525	4.94E-12
Cellular component	plastid	GO:0009536	2563	2.94E-11
Cellular component	photosynthetic membrane	GO:0034357	390	2.23E-11
Cellular component	thylakoid membrane	GO:0042651	390	2.23E-11
Biological process	response to chitin	GO:0010200	422	8.25E-06
Biological process	small molecule metabolic process	GO:0044281	2471	1.36E-05
Biological process	photosynthesis	GO:0015979	216	9.75E-06
Biological process	single-organism biosynthetic process	GO:0044711	2546	9.16E-06
Biological process	response to water deprivation	GO:0009414	988	7.71E-06

l. 416/417: using 0.1 as a cutoff for "strong purifying selection" is arbitrary -what is the justification.

>>> Thank you for the comment. In general, a Ka/Ks value less than 1 meant that genes have experienced some purifying selection, with lower Ka/Ks value being a sign of increasing purifying

selection. There is unfortunately no gold standard for “strong purifying selection”, and various cutoffs of Ka/Ks have been used as indicative of “strong purifying selection”; for instance, Imran et al. (2016) and Meng et al. (2019) used 0.3. Although using a lower value of 0.1 here in our study is also subjective, our purpose is to ensure the highest reliability of our results. To avoid potential misunderstanding, we do not use “strong” to avoid overstated claims.

References:

(1) Imran M, Tang K, Liu JY. Comparative Genome-Wide Analysis of the Malate Dehydrogenase Gene Families in Cotton. Plos one. 2016 ;11(11):e0166341. DOI: 10.1371/journal.pone.0166341.

(2) Meng, D., Cao, Y., Chen, T. et al. Evolution and functional divergence of MADS-box genes in Pyrus. Sci Rep 9, 1266 (2019). https://doi.org/10.1038/s41598-018-37897-6

l. 433 I don’t understand what evidence the authors have that DEA are time or tissue specific.

>>>Thank you. What we meant is that the expression of DEAs is time or tissue specific, not the DEAs themselves, because our DEAs were identified by comparing their expression, and the quantity of DEAs varied among different samples (totally 39 samples).

l. 542: just having Ka/Ks > 1 is not enough evidence for stating that the gene was targeted by positive selection. The authors need to use additional analyses to provide significant evidence using selective sweep detection methods like Sweepfinder2 (http://degiorgiogroup.fau.edu/sf2.html). This applies to other parts of the manuscript.

>>> Thank you for your suggestion. We conducted selective sweep analysis using Sweepfinder2, and we indeed obtained a sweep region overlapping with the VRN1 cluster region we identified (as shown in the figure below). We added this information in our revision at Lines 548-549.

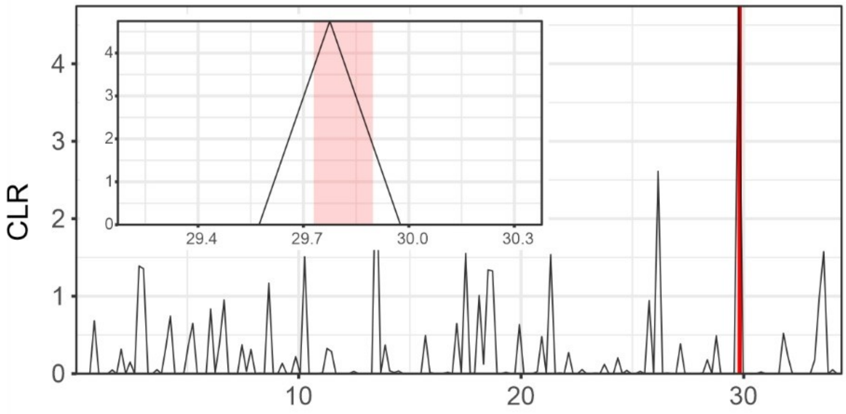

Note: Sweep analysis was conducted using CLR (Composite Likelihood Ratio) test by Sweepfinder2 for Chr 5 with a window size of 200 kb. The VRN1 cluster region is highlighted by the light-pink box.

l. 550: What is the value of reporting non significant associations?

>>> In total 1,913,230 SNPs were used in our GWAS analyses. We used 0.05 calculated from the Bonferroni P-value corrected method as a threshold, that is -log10(0.05/1913230) = 7.58. And we also revised the text at Line 557-560.

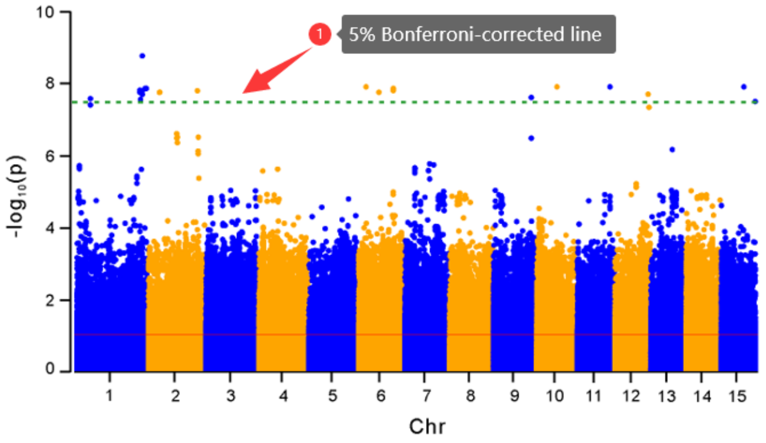

Reviewer #3:
Remarks to the Author:
Hu et al. report a genome assembly for the specialty fruit crop lychee and utilize resequencing and comparative genomics to uncover the domestication history and genomic basis for flowering time variation in lychee, respectively. The authors produced a fully phased, chromosome-scale assembly of the highly heterozygotic lychee genome and uncovered extensive allele specific expression. They provide evidence for two domestication events in lychee and identified homologs of flowering time genes in Arabidopsis that may be related to flowering time differences between early and late maturing cultivars. I read this paper with interest, but I have a few concerns that should be addressed before publication.

1. I have some concerns about the genome assembly and haplotype phasing. It is unclear based on the results and methods how one haplotype was extracted from the assembly and used for Hi-C anchoring. Based on the Hi-C contact matrix and BUSCO score, it seems like HaploMerger2 successfully binned the genome into two haplotypes, but each bin of contigs likely represents a chimera of the HY and HH haplotypes stitched together. Based on the raw PacBio assembly size, both the HY and HH haplotypes were assembled for 'Feizixiao', so couldn't these be used as a basis for haplotype phasing? This would give the authors a better sense of copy number variations, TE polymorphisms, large-scale rearrangements, and other differences between the HY and HH haplotypes in 'Feizixiao' compared to using Illumina data alone. This may also improve alignment of reads for the resequencing data and analysis of differentially expressed alleles. If the within genome heterozygosity is truly ~2.2%, the HiC data should be sufficient for phasing HY and HH from the original assembly, especially if the authors use ALLHiC (which was created by several coauthors of this manuscript). There are very few phased haplotype genome assemblies for plants and additional analyses using an improved assembly would strengthen the manuscript.
Details on haplotype phasing and verification are vague. The authors state HapCUT2 was used for phasing and 'eventually, 15 pairs of homologous chromosomes were obtained'. If the original haploid assembly was a chimera, wouldn't these chimeric regions be carried over in the resulting

HY and HH haplotypes? I am unsure why aligned Illumina data was used for phasing when both alleles were already assembled in the raw PacBio contigs.

>>>Thank you for your comments and suggestions regarding the phasing of haplotype genomes. Indeed, as described above, we found assembly chimerism when using CANU and all PacBio reads for contig assembly. Therefore, we originally had phased haplotype specific SNPs identified from HiC data onto our original reference genome to obtain the two haplotypic genomes originally reported, but we lost a considerable amount of genomic information regarding structural variation, copy number variation, and large-scale rearrangements in the process.

In our revision, we tested ALLHiC as the reviewer suggested. Unfortunately it failed to work, as expected from our previous attempts. We then designed a new strategy to conduct the haplotype phasing. In brief, as described above, we first separated the raw PacBio long reads into two groups using haplotypic SNPs identified from HiC and 10X Genomics data, and then performed guided genome assembly separately for each of the reads group. In this way, two independently assembled haplotypic genomes were obtained. For detailed description of the process, please refer to our response to the editor's #1 priority above.

We also explored CNVs, SVs and rearrangements between the two newly assembled haplotype genomes. All the related results were provided in Supplemental table 20-22, and corresponding description was added in Line 368-377.

2. The results on maturation time are interesting, but the finding that CONSTANS genes contribute to maturity time is not well supported. It is not surprising that the authors found no statistically significant GWAS peaks for flowering time given the low sample size (62 based on the methods) and significant population structure between the wild and independently domesticated cultivars. The authors identified one flowering time related gene from the top 20 GWAS peaks, but this is not unexpected since the genome contains so many flowering genes. The heterozygous deletion in one haplotype is interesting, but because both alleles are expressed, it's difficult to say if this plays a role in flowering time variation. Additional evidence would help strengthen the claim that this gene/gene pair is involved in maturation.

>>> Thank you for this comment. We fully agree that further functional validation will be required to confirm the role of these *CONSTANS-like* genes in lychee flowering regulation. However, as a perennial woody fruit crop, it is currently intractable to perform functional characterization in lychee using a gene overexpression or knock-out/knock-down or similar strategy. So far, there is not even a stable transgenic system available, although we are working hard on developing one.

However, although >500 flowering-related genes were identified from the lychee genome, they were very rarely located close to the top 20 GWAS signals. In a ±15 kb spanning region, 27 annotated genes were found close to these 20 signals. Among them, only one gene (COL307) was a flowering-related gene. If we extended the spanning region to ±50 kb, COL307 was still one of the two flowering-related genes among all 109 associated genes. Two more flowering-related genes could be found if a window size of ±100 kb was chosen, including the COL305 gene. In short, four flowering-related genes are located in the ±100 kb regions spanning the top 20 GWAS

signals. Among them, LITCHI023128 (TPS1_ARATH) is not a differentially expressed allelic gene. Although we cannot exclude the possibility that G2OX2 (LITCHI026618) is phenotypically important, we suggest that the two COL genes are clearly better candidate genes, especially given our finding of the association with the 3.7kb deletion.

**Gene ID**	**Swiss_Des**	**Annotation**	**Distance to the closest GWAS signal**	**in ±50kb region**	**in ±100kb region**
LITCHI019307	COL2_ARATH	Zinc finger protein CONSTANS-LIKE 2	12.1 kb	YES	YES
LITCHI023128	TPS1_ARATH	Alpha, alpha-trehalose-phosphate	28.9 kb	YES	YES
LITCHI019305	COL2_ARATH	Zinc finger protein CONSTANS-LIKE 2	65.5 kb	NO	YES
LITCHI026618	G2OX2_PEA	Gibberellin 2-beta-dioxygenase 2	93 kb	NO	YES

3. The manuscript is quite long overall, and some sections contain extraneous text that distracts from the most meaningful findings. For instance, the conservation of a VRN1-like gene cluster across eudicots is interesting but does not yield meaningful results in lychee. The number of VRN1- like genes is similar in lychee and other genomes and many of these genes are expressed across diverse tissues, so it is unclear what role this gene cluster may play in flowering time regulation compared to other species.

>>>Thank you. While we do agree that the conservation of the *VRN1-like* gene cluster does not yield distinctive results for lychee per se, but this is an exceptional clade-specific finding which may have broad meaning to the plant community. First, *VRN1* is a functionally essential gene in vernalization (temperature responses) as proven in Arabidopsis. Its highly conserved synteny among many eudicots implies that these syntenic *VRN1* genes likely have similar essential (conserved) functions among eudicot plants. Second, as indicated previously, lychee is the most agriculturally important crop in Sapindaceae, a huge family of flowering plants (including maple) that consists of 138 genera and ~2000 accepted species. We have intended that our detailed study of the lychee genome would provide in-depth genomic relevance for Sapindaceae species in general. The specific expansion of the VRN1 gene cluster suggests that there are probably some Sapindaceae-specific traits regulated by this particular set of duplicated *VRN1* genes. The diverse expression of these *VRN1-like* genes indeed implies more complexity in their function, which may be different from their orthologous counterpart, the *VRN1* gene in Arabidopsis, and they may well have broader functions other than simply vernalization (temperature responses). Even if the main roles of this *VRN1-like* gene cluster are not fully associated with flowering, the process of tandem duplication and neo/subfunctionalization provide a novel and unique case to study the evolutionary characteristics of a functionally relevant gene family.

Minor:

It is difficult to distinguish the wild from cultivated, maturity classes, and country of origin of the

accessions in Figure 2A. I like the concept of using different fruit and leaf colors, but it is hard to interpret.

>>>Thank you, we have revised the figure by highlighting the different groups with different shade colors.

Lychee is a specialty fruit and I suspect many readers will be unfamiliar with the different cultivars of lychee and their distinguishing characteristics. It may be helpful to include pictures of representative cultivar groups for the wild, extremely early, early, and late-maturing groups in one of the figures.

>>>Thank you for this suggestion. We have added a few representative photos to show lychee fruit and its diversity in Fig. 1A and supplementary figure 1.

natureresearch

Decision Letter, first revision:

8th Apr 2021

Dear Professor Xia,

Your Article, "Two divergent haplotypes from a highly heterozygous lychee genome point to independent domestication events for early and late-maturing cultivars" has now been seen by 3 referees. You will see from their comments below that while they find your work of interest, some important points are raised. We are interested in the possibility of publishing your study in Nature Genetics, but would like to consider your response to these concerns in the form of a revised manuscript before we make a final decision on publication.

As you will see from these comments, reviewer #1 has concerns regarding the number of genes in the reference genome and the two haplotypes. Reviewer #2 points out essential technical issues that need to be addressed. In addition, please try to improve the biological insights if possible.

We therefore invite you to revise your manuscript taking into account all reviewer and editor comments. Please highlight all changes in the manuscript text file. At this stage we will need you to upload a copy of the manuscript in MS Word .docx or similar editable format.

We are committed to providing a fair and constructive peer-review process. Do not hesitate to contact us if there are specific requests from the reviewers that you believe are technically impossible or unlikely to yield a meaningful outcome.

When revising your manuscript:

*1) Include a “Response to referees” document detailing, point-by-point, how you addressed each referee comment. If no action was taken to address a point, you must provide a compelling argument. This response will be sent back to the referees along with the revised manuscript.

*2) If you have not done so already please begin to revise your manuscript so that it conforms to our Article format instructions, available
here.
Refer also to any guidelines provided in this letter.

*3) Include a revised version of any required Reporting Summary:
https://www.nature.com/documents/nr-reporting-summary.pdf
It will be available to referees (and, potentially, statisticians) to aid in their evaluation if the manuscript goes back for peer review.
A revised checklist is essential for re-review of the paper.

Please be aware of our guidelines on digital image standards.

Please use the link below to submit your revised manuscript and related files:

[REDACTED]

Note: This URL links to your confidential home page and associated information about manuscripts you may have submitted, or that you are reviewing for us. If you wish to forward this email to co-authors, please delete the link to your homepage.

We hope to receive your revised manuscript within four to eight weeks. If you cannot send it within this time, please let us know.

Please do not hesitate to contact me if you have any questions or would like to discuss these revisions further.

Nature Genetics is committed to improving transparency in authorship. As part of our efforts in this direction, we are now requesting that all authors identified as 'corresponding author' on published papers create and link their Open Researcher and Contributor Identifier (ORCID) with their account on the Manuscript Tracking System (MTS), prior to acceptance. ORCID helps the scientific community achieve unambiguous attribution of all scholarly contributions. You can create and link your ORCID from the home page of the MTS by clicking on 'Modify my Springer Nature account'. For more information please visit please visit www.springernature.com/orcid.

We look forward to seeing the revised manuscript and thank you for the opportunity to review your work.

Sincerely,

Wei Li, PhD
Senior Editor
Nature Genetics
One New York Plaza, 47th Fl.
New York, NY 10004, USA
www.nature.com/ng

Reviewers' Comments:

Reviewer #1:
Remarks to the Author:
I think the authors have done their utmost best to address all issues raised by the reviewers and most of my previous comments have been adequately addressed. However, there is one remaining issue: the authors should still try to better explain how they derive the number of genes reported in the manuscript. In the previous version, the authors reported 31,896 genes in the reference genome and

the two haplotypes, and I asked about the number of 'identical genes' in the both haplotypes. In the revised version of the manuscript, the authors report 31,896 genes in the reference genome, but 27,687 and 27,711 genes in HY and HH, respectively. Maybe they could clarify the relationship between the reference genome and the two 'haplotype genomes', and what causes the decrease of gene numbers in the latter? Also, they found 40.5% duplicated BUSCOs based on the 31,896 genes, in contrast to 2.5% and 2.2% in HY and HH. Is it possible that there are many allelic gene 'variants' in the 31,896 reference gene set? My 'gut' feeling is that the lychee genome should have about 27,000 or 28.000 genes, rather than about 32,000 genes, but I'd be happily proven wrong.

Reviewer #2:
Remarks to the Author:
The manuscript has improved considerably with revision.

Some essential remaining points:

1. pi_n/pi_s

They write that they developed a new script to calculate this statistic and that the source code is available on github. The repository but it is empty, so could not check the code. (https://github.com/jsalojar/PiNSiR)

2. Theoretical results about the relation between diversity and selfing exist. They demonstrate how a rescaling of selfing (and recombination if relevant) can be used to account for self-fertilization. I therefore recommend to revise the part of the rescaling of mutation rate to account for selfing after reading Nordborg and Donnelly 1999 (Genetics)
https://www.ncbi.nlm.nih.gov/pmc/articles/PMC1208046/

3. The sweepfinder2 analyses needs statistical threshold. Critical values at 5% significance level can be obtained based on neutral simulations using the best demographic model. See Huber et al (2015) for an example: https://onlinelibrary.wiley.com/doi/full/10.1111/mec.13351.

Sweepfinder (or SweeD) can be run on the simulated data and the 95th percentile can be used as cutoff value.

Reviewer #3:
Remarks to the Author:
I thank the authors for their detailed responses and efforts to improve the quality of the genome assembly and phasing as well as downstream population genetics and comparative genomics analyses. The manuscript is overall much improved, and the authors have addressed my previous comments/concerns.

Author Rebuttal, first revision:

Dear Editors and Reviewers,

Thank you for your time and effort for handling our manuscript one more time. We are happy to know that the reviewers are satisfactory with most of our responses and revisions. Below are our responses to the new comments, with the reviewers' comments in dark and our responses preceded by ">>>" in blue.

Reviewers' Comments:

Reviewer #1:
Remarks to the Author:
I think the authors have done their utmost best to address all issues raised by the reviewers and most of my previous comments have been adequately addressed. However, there is one remaining issue: the authors should still try to better explain how they derive the number of genes reported in the manuscript. In the previous version, the authors reported 31,896 genes in the reference genome and the two haplotypes, and I asked about the number of 'identical genes' in the both haplotypes. In the revised version of the manuscript, the authors report 31,896 genes in the reference genome, but 27,687 and 27,711 genes in HY and HH, respectively. Maybe they could clarify the relationship between the reference genome and the two 'haplotype genomes', and what causes the decrease of gene numbers in the latter? Also, they found 40.5% duplicated BUSCOs based on the 31,896 genes, in contrast to 2.5% and 2.2% in HY and HH. Is it possible that there are many allelic gene 'variants' in the 31,896 reference gene set? My 'gut' feeling is that the lychee genome should have about 27,000 or 28.000 genes, rather than about 32,000 genes, but I'd be happily proven wrong.

>>> Thanks for this comment. There are 31,896 gene models in the reference gene set, which contains 59,630 transcripts, because we predicted alternatively spliced variants when doing genome-wide gene annotation. We used those transcripts for BUSCOs assessment of the transcriptome quality and this is the reason why there are 40.5% duplicate BUSCOs for the reference gene set. However, when assigning gene models from the reference genome to the haplotypes, it is hard to take these alternatively spliced transcripts into account, and we therefore removed them and kept only the longest transcripts as representative gene models. As we used only the core set of gene models (27,687 and 27,711 genes in HY and HH, respectively) for BUSCOs analysis, the number of duplicate BUSCOs of haplotype assemblies dropped down a lot. For the reference gene set, if we only kept the longest transcripts, the duplicated BUSCOs would drop as well from 40.5% to 5.8% (shown in the table below).

BUSCO analysis of genome annotation (protein)(eudicotyledons_odb10)

Description	Ref. (59630 transcripts)		Ref. (31896 transcripts)	
	Number	Per. (%)	Number	Per. (%)
Complete BUSCOs (C)	2010	94.8%	2002	94.4%
Complete and single-copy BUSCOs (S)	1152	54.3%	1880	88.6%
Complete and duplicated BUSCOs (D)	858	40.5%	122	5.8%
Fragmented BUSCOs (F)	41	1.9%	48	2.3%
Missing BUSCOs (M)	70	3.3%	71	3.3%
Total BUSCO groups searched	2121	100.0%	2121	100.0%

As for ‘what causes the decrease of gene numbers in the haplotype assemblies?’, we think there are two main reasons: (1) the haplotype genomes are not as complete as the reference genome. Missing sequences likely causes incorrect or incomplete gene assignment; (2) as shown in our results, there are plenty of variants including SNPs/Indels and structural variants between HY/HH haplotype genomes and the reference, which can also result in missing genes when mapping gene models from the reference to the haplotype genomes (as shown in the figure below for three representative chromosomes).

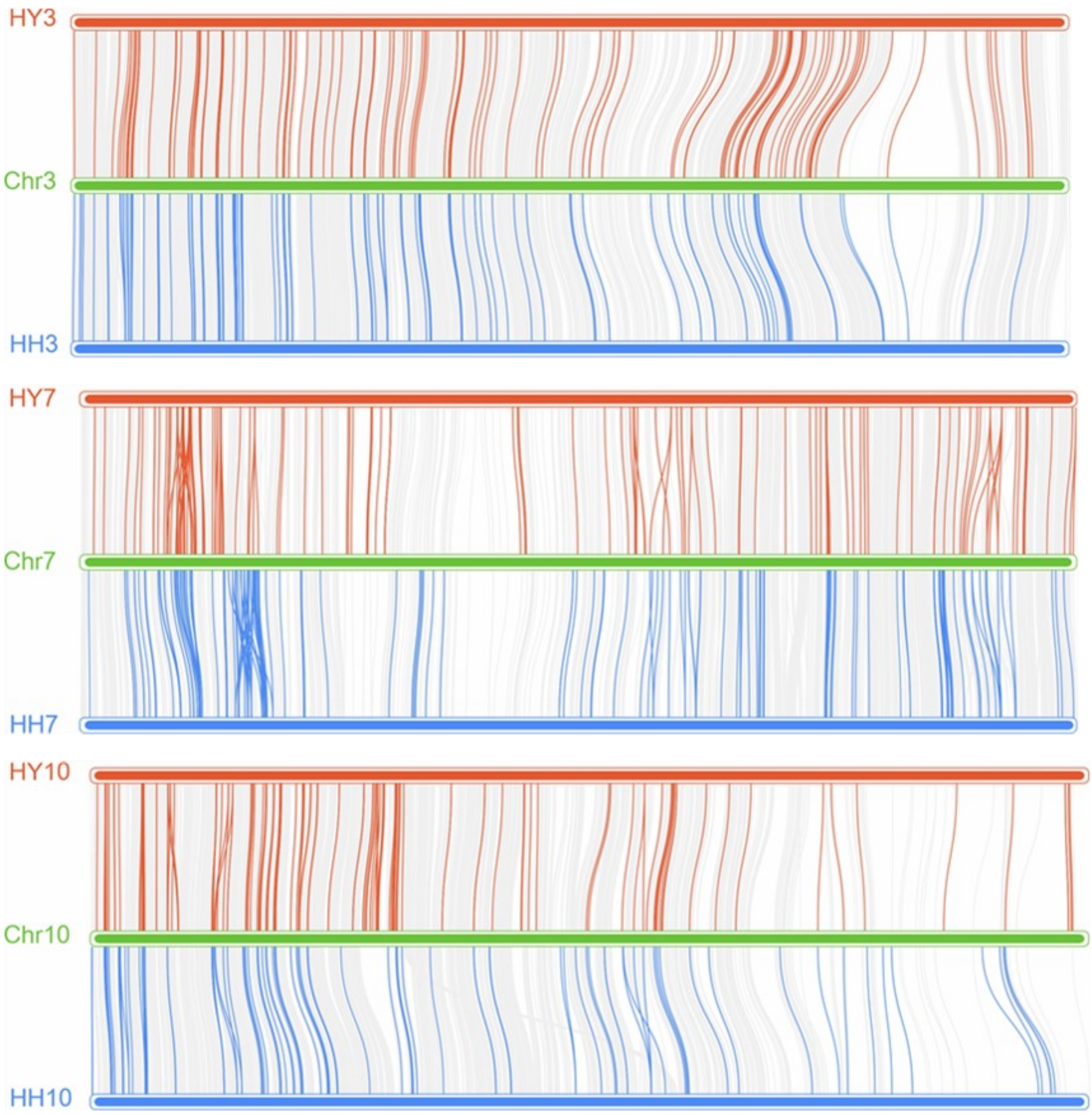

Reviewer #2:
Remarks to the Author:
The manuscript has improved considerably with revision.

Some essential remaining points:

1. pi_n/pi_s

They write that they developed a new script to calculate this statistic and that the source code is available on github. The repository but it is empty, so could not check the code. (https://github.com/jsalojar/PiNSiR)

>>> Thanks. Since this publication will be the original reference to the software, we have been working on the code and documentation without making it available before acceptance of the publication. The github site is now updated with the first version of the pipeline and we are currently working on a more user-friendly version of the software, to be published with the initial publication of this genome paper. In future the pipeline will not need as many external components. The benefit of the current version is that it relies on generally accepted tools and thus it can be used to verify the results from future development versions.

2. Theoretical results about the relation between diversity and selfing exist. They demonstrate how a rescaling of selfing (and recombination if relevant) can be used to account for self-fertilization. I therefore recommend to revise the part of the rescaling of mutation rate to account for selfing after reading Nordborg and Donnelly 1999 (Genetics) https://www.ncbi.nlm.nih.gov/pmc/articles/PMC1208046/

>>> We thank the referee very much for the suggestion, this provides a theoretical framework for our results. We have adjusted the supplementary note discussing the rescaling accordingly. In the main paper, we added a reference to the Nordborg and Donnelly paper and revised the sentence discussing this part to "Here, motivated by coalescent process with selfing , we developed an ad hoc approach to compensate for this, but further model development is necessary to formally incorporate the differences in reproductive strategies among populations into modeling.".

3. The sweepfinder2 analyses needs statistical threshold. Critical values at 5% significance level can be obtained based on neutral simulations using the best demographic model. See Huber et al (2015) for an example: https://onlinelibrary.wiley.com/doi/full/10.1111/mec.13351. Sweepfinder (or SweeD) can be run on the simulated data and the 95th percentile can be used as cutoff value.

>>> Thank you, we have now estimated the critical value as recommended. To simulate neutral model, we fitted a Fastsimcoal2 model to the observed site frequency spectrum with population size changes initialized to roughly the times illustrated in Fig 2d; the best parameter settings were then found by maximum likelihood fits starting from 100 independent random starting values. To take the inbreeding into account we assumed a decreasing population size in Yunnan population after the split. The best model had a population split at 1975 generations, and the population size changes followed the SMC++ trajectory. Using the neutral model we simulated 1,000 1Mb genome blocks with MSMS and calculated Sweepfinder 2 statistics from the simulated data. We assumed two recombination events per chromosome, this produced very similar values per bp as has been given for peach (Wang et al., 2016) and Arabidopsis (Rowan et al., 2019) - the value is likely an overestimate, since for Arabidopsis the average number of recombination events per chromosome is less than two, and the recombination rate has been speculated to be even lower for long-lived trees (Wang et al. 2016). To reduce the uncertainty related with the unknown mutation rate we simulated the number of observed SNPs per Mb instead, as this is possible in MSMS.
In simulated data, the first and last Sweepfinder2 statistic estimation points were close to chromosome ends (and thus not the full window was not used) and were therefore dropped out;

three middle evaluation positions (at 250, 500 and 750 kp) were retained. The neutral simulations produced 3,000 Sweepfinder2 likelihood ratio (LR) statistic values and a critical 95% value of 0.82. The LR statistic for the VRN region in lychee was 1.333696, corresponding to 97.8% critical value. We have now added this description in the Methods section and illustrate the critical value in Supplementary Figure 21

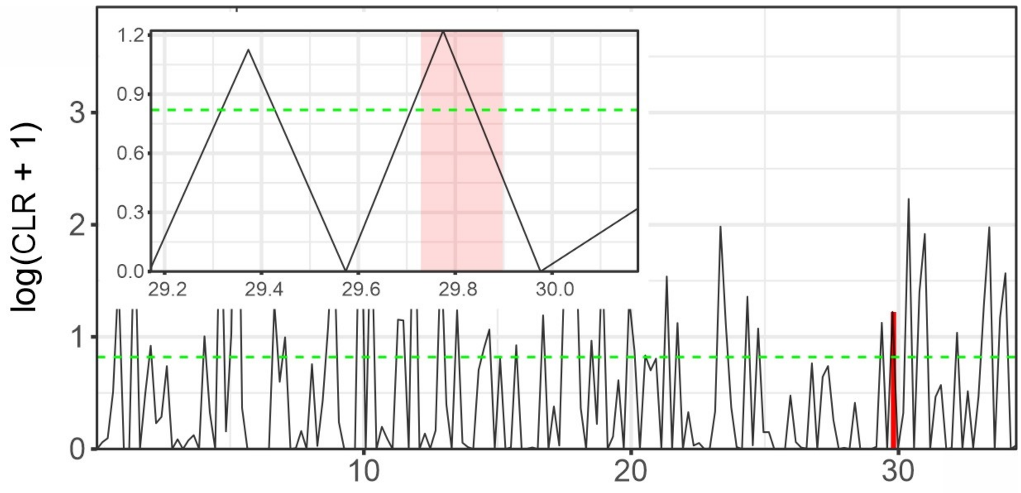

References:

Wang Long, Zhang Yanchun, Qin Chao, Tian Dacheng, Yang Sihai and Hurst Laurence D. 2016Mutation rate analysis via parent–progeny sequencing of the perennial peach. II. No evidence for recombination-associated mutation. Proc. R. Soc. B.283:20161785-20161785

Beth A Rowan, Darren Heavens, Tatiana R Feuerborn, Andrew J Tock, Ian R Henderson, Detlef Weigel, An Ultra High-Density Arabidopsis thaliana Crossover Map That Refines the Influences of Structural Variation and Epigenetic Features, Genetics, Volume 213, Issue 3, 1 November 2019, Pages 771–787

Reviewer #3:

Remarks to the Author:

I thank the authors for their detailed responses and efforts to improve the quality of the genome assembly and phasing as well as downstream population genetics and comparative genomics analyses. The manuscript is overall much improved, and the authors have addressed my previous comments/concerns.

Decision Letter, second revision:

Our ref: NG-A54825R1

25th Jun 2021

Dear Dr. Xia,

Thank you for submitting your revised manuscript "Two divergent haplotypes from a highly heterozygous lychee genome point to independent domestication events for early and late-maturing cultivars" (NG-A54825R1). It has now been seen by the original referees. The reviewers find that the paper has improved in revision, and therefore we'll be happy in principle to publish it in Nature Genetics, pending minor revisions to satisfy the referees' final requests and to comply with our editorial and formatting guidelines.

If the current version of your manuscript is in a PDF format, please email us a copy of the file in an editable format (Microsoft Word or LaTex)-- we can not proceed with PDFs at this stage.

We are now performing detailed checks on your paper and will send you a checklist detailing our editorial and formatting requirements in about a week. Please do not upload the final materials and make any revisions until you receive this additional information from us.

Thank you again for your interest in Nature Genetics Please do not hesitate to contact me if you have any questions.

Sincerely,

Wei Li, PhD
Senior Editor
Nature Genetics
One New York Plaza, 47th Fl.
New York, NY 10004, USA
www.nature.com/ng

Final Decision Letter:

In reply please quote: NG-A54825R2 Xia

19th Oct 2021

Dear Dr. Xia,

I am delighted to say that your manuscript "Two divergent haplotypes from a highly heterozygous lychee genome suggest independent domestication events for early and late-maturing cultivars" has

been accepted for publication in an upcoming issue of Nature Genetics.

Prior to setting your manuscript, we may make minor changes to enhance the lucidity of the text and with reference to our house style. We therefore ask that you examine the proofs most carefully to ensure that we have not inadvertently altered the sense of your text in any way.

Once your manuscript is typeset and you have completed the appropriate grant of rights, you will receive a link to your electronic proof via email with a request to make any corrections within 48 hours. If, when you receive your proof, you cannot meet this deadline, please inform us at rjsproduction@springernature.com immediately.

Your paper will be published online after we receive your corrections and will appear in print in the next available issue. You can find out your date of online publication by contacting the Nature Press Office (press@nature.com) after sending your e-proof corrections. Now is the time to inform your Public Relations or Press Office about your paper, as they might be interested in promoting its publication. This will allow them time to prepare an accurate and satisfactory press release. Include your manuscript tracking number (NG-A54825R2) and the name of the journal, which they will need when they contact our Press Office.

Before your paper is published online, we shall be distributing a press release to news organizations worldwide, which may very well include details of your work. We are happy for your institution or funding agency to prepare its own press release, but it must mention the embargo date and Nature Genetics. Our Press Office may contact you closer to the time of publication, but if you or your Press Office have any enquiries in the meantime, please contact press@nature.com.

Acceptance is conditional on the data in the manuscript not being published elsewhere, or announced in the print or electronic media, until the embargo/publication date. These restrictions are not intended to deter you from presenting your data at academic meetings and conferences, but any enquiries from the media about papers not yet scheduled for publication should be referred to us.

Please note that <i>Nature Genetics</i> is a Transformative Journal (TJ). Authors may publish their research with us through the traditional subscription access route or make their paper immediately open access through payment of an article-processing charge (APC). Authors will not be required to make a final decision about access to their article until it has been accepted. Find out more about Transformative Journals

<B>Authors may need to take specific actions to achieve compliance with funder and institutional open access mandates. For submissions from January 2021, if your research is supported by a funder that requires immediate open access (e.g. according to Plan S principles) then you should select the gold OA route, and we will direct you to the compliant route where possible. For authors selecting the subscription publication route our standard licensing terms will need to be accepted, including our self-archiving policies. Those standard licensing terms will supersede any other terms that the author or any third party may assert apply to any version of the manuscript.

Please note that Nature Research offers an immediate open access option only for papers that were first submitted after 1 January, 2021.

You will not receive your proofs until the publishing agreement has been received through our system.

If you have any questions about our publishing options, costs, Open Access requirements, or our legal forms, please contact ASJournals@springernature.com

If you have posted a preprint on any preprint server, please ensure that the preprint details are updated with a publication reference, including the DOI and a URL to the published version of the article on the journal website.

To assist our authors in disseminating their research to the broader community, our SharedIt initiative provides you with a unique shareable link that will allow anyone (with or without a subscription) to read the published article. Recipients of the link with a subscription will also be able to download and print the PDF.

As soon as your article is published, you will receive an automated email with your shareable link.

You can now use a single sign-on for all your accounts, view the status of all your manuscript submissions and reviews, access usage statistics for your published articles and download a record of your refereeing activity for the Nature journals.

An online order form for reprints of your paper is available at https://www.nature.com/reprints/author-reprints.html. Please let your coauthors and your institutions' public affairs office know that they are also welcome to order reprints by this method.

If you have not already done so, we invite you to upload the step-by-step protocols used in this manuscript to the Protocols Exchange, part of our on-line web resource, natureprotocols.com. If you complete the upload by the time you receive your manuscript proofs, we can insert links in your article that lead directly to the protocol details. Your protocol will be made freely available upon publication of your paper. By participating in natureprotocols.com, you are enabling researchers to more readily reproduce or adapt the methodology you use. Natureprotocols.com is fully searchable, providing your protocols and paper with increased utility and visibility. Please submit your protocol to https://protocolexchange.researchsquare.com/. After entering your nature.com username and password you will need to enter your manuscript number (NG-A54825R2). Further information can be found at https://www.nature.com/nprot/.

Sincerely,

Wei Li, PhD
Senior Editor
Nature Genetics

naturereseach

New York, NY 10004, USA
www.nature.com/ng
